# MAPK Signaling Pathway Alters Expression of Midgut ALP and ABCC Genes and Causes Resistance to *Bacillus thuringiensis* Cry1Ac Toxin in Diamondback Moth

**DOI:** 10.1371/journal.pgen.1005124

**Published:** 2015-04-13

**Authors:** Zhaojiang Guo, Shi Kang, Defeng Chen, Qingjun Wu, Shaoli Wang, Wen Xie, Xun Zhu, Simon W. Baxter, Xuguo Zhou, Juan Luis Jurat-Fuentes, Youjun Zhang

**Affiliations:** 1 Department of Plant Protection, Institute of Vegetables and Flowers, Chinese Academy of Agricultural Sciences, Beijing, China; 2 School of Biological Sciences, University of Adelaide, Adelaide, South Australia, Australia; 3 Department of Entomology, University of Kentucky, Lexington, Kentucky, United States of America; 4 Department of Entomology and Plant Pathology, University of Tennessee, Knoxville, Tennessee, United States of America; The University of North Carolina at Chapel Hill, UNITED STATES

## Abstract

Insecticidal crystal toxins derived from the soil bacterium *Bacillus thuringiensis* (Bt) are widely used as biopesticide sprays or expressed in transgenic crops to control insect pests. However, large-scale use of Bt has led to field-evolved resistance in several lepidopteran pests. Resistance to Bt Cry1Ac toxin in the diamondback moth, *Plutella xylostella* (L.), was previously mapped to a multigenic resistance locus (*BtR-1*). Here, we assembled the 3.15 Mb *BtR-1* locus and found high-level resistance to Cry1Ac and Bt biopesticide in four independent *P*. *xylostella* strains were all associated with differential expression of a midgut membrane-bound alkaline phosphatase (*ALP*) outside this locus and a suite of ATP-binding cassette transporter subfamily C (ABCC) genes inside this locus. The interplay between these resistance genes is controlled by a previously uncharacterized *trans*-regulatory mechanism via the mitogen-activated protein kinase (MAPK) signaling pathway. Molecular, biochemical, and functional analyses have established ALP as a functional Cry1Ac receptor. Phenotypic association experiments revealed that the recessive Cry1Ac resistance was tightly linked to down-regulation of *ALP*, *ABCC2* and *ABCC3*, whereas it was not linked to up-regulation of *ABCC1*. Silencing of *ABCC2* and *ABCC3* in susceptible larvae reduced their susceptibility to Cry1Ac but did not affect the expression of *ALP*, whereas suppression of *MAP4K4*, a constitutively transcriptionally-activated MAPK upstream gene within the *BtR-1* locus, led to a transient recovery of gene expression thereby restoring the susceptibility in resistant larvae. These results highlight a crucial role for *ALP* and ABCC genes in field-evolved resistance to Cry1Ac and reveal a novel *trans*-regulatory signaling mechanism responsible for modulating the expression of these pivotal genes in *P*. *xylostella*.

## Author Summary

Biopesticide and transgenic crops based on *Bacillus thuringiensis* (Bt) Cry toxins are widely used worldwide, yet the development of field resistance seriously threatens their sustainability. Unraveling these resistance mechanisms are of great importance for delaying insect field resistance evolution. The diamondback moth was the first insect to evolve field resistance to Bt biopesticides and it is an excellent model for the study of Bt resistance mechanisms. In this work, we present strong empirical evidence supporting that (1) field-evolved resistance to Bt in *P*. *xylostella* is tightly associated with differential expression of a membrane-bound alkaline phosphatase (*ALP*) and a suite of ATP-binding cassette transporter subfamily C (ABCC) genes, and (2) a constitutively transcriptionally-activated upstream gene (*MAP4K4*) in the MAPK signaling pathway is responsible for this *trans*-regulatory signaling mechanism. These findings identify key resistance genes and provide the first comprehensive mechanistic description responsible for the field-evolved Bt resistance in *P*. *xylostella*. Given that expression alterations of multiple receptor genes result in Bt resistance in many other insects, it can now be tested to determine whether the previously unidentified *trans*-regulatory mechanism characterized in this study is also involved in these cases.

## Introduction

The Gram-positive entomopathogen *Bacillus thuringiensis* (Bt) is the most widely used biopesticide due to its highly specific activity and environmental safety [1]. The insecticidal activity of Bt is largely attributed to diverse δ-endotoxins (Cry toxins) produced during sporulation [2]. Transgenic crops harboring Bt toxin genes (Bt crops) are the most successful insecticidal biotechnology, with >75 million hectares planted worldwide [3]. However, high adoption of Bt crops and concurrent use of Bt pesticides represent high selection pressure for insect resistance evolution. To date, cases of field-evolved resistance to Bt sprays or Bt crops have been reported in at least seven insect species [4,5]. The economic and environmental importance of Bt insecticides highlight the significance of clarifying the molecular mechanisms of insect field-evolved resistance to Bt.

The mode of action of Bt Cry toxins includes a critical binding step to receptors in the insect midgut, which is conducive to formation of a toxin pore on the enterocyte membrane that leads to osmotic cell death [6]. The importance of this binding step is further highlighted by high levels of resistance to Bt Cry toxins being almost exclusively associated with alterations in receptor genes [7]. While a number of insect midgut proteins have been proposed as functional receptors for diverse Cry toxins [8], high levels of resistance to Cry1 toxins due to reduced toxin binding have been genetically linked to mutations or expression alterations of receptor genes such as cadherin, aminopeptidase-N (*APN*) and alkaline phosphatase (*ALP*) [6]. Recently, mutations in ATP-binding cassette transporter subfamily C member 2 (*ABCC2*) gene [9–13] have been reported to be linked to high levels of resistance to Bt Cry toxins in diverse lepidopteran insects, and it has been proved to be a functional receptor for Bt Cry toxins in *Bombyx mori* [14]. Although expression alterations of ABCC genes have been reported to result in chemical insecticide resistance in many insects [15,16], whether the expression alterations of ABCC genes can be involved in insect Bt resistance is unclear. In addition, although altered *ALP* gene expression seems to be commonly associated with lepidopteran resistance to Cry1 toxins [17,18], there is currently no available functional or genetic evidence for these ALP proteins in Bt resistance.

The diamondback moth, *Plutella xylostella* (Lepidoptera: Plutellidae), is a global notorious pest that can rapidly evolve resistance to insecticides and cause US $4–5 billion in management costs annually [19]. Thus far, field-evolved resistance to Bt sprays has only been described in *P*. *xylostella* [20] and greenhouse populations of *Trichoplusia ni* [21]. In both cases, resistance was monogenic and transmitted as an autosomal recessive trait associated with reduced toxin binding to the midgut [22,23]. In *T*. *ni*, this reduced Cry1Ac toxin binding is associated with reduced expression of a midgut aminopeptidase gene (*APN1*) [24] possibly *trans*-regulated by an unidentified gene located in a resistance locus containing the *ABCC2* gene [10]. In *P*. *xylostella*, *cis*-acting mutations in putative toxin receptor genes are not linked to field-evolved resistance to Cry1Ac [25,26]. As in *T*. *ni*, resistance to Cry1Ac in *P*. *xylostella* also mapped to a multigenic resistance locus (*BtR-1*) containing the *ABCC2* gene [10]. However, the detailed genetic makeup of this resistance locus and the potential *trans*-acting regulatory effect on Cry toxin receptors by resident genes remain unknown.

High-level resistance phenotype to Bt Cry toxins in insects is often autosomal recessive and controlled by a single gene, however, the fact that many players responsible for resistance suggests there may be a common pathway that links all these receptor genes. Mitogen-activated protein kinase (MAPK) signaling pathway has been described to control immune defensive responses to Bt Cry toxins in nematodes [27,28] and insects [29,30]. The MAPK signaling pathway consists of a four-kinase cascade module (MAP4K, MAP3K, MAP2K and MAPK) that can positively or negatively regulate expression of diverse functional genes via different transcription factors [31,32]. Therefore, it is plausible that the MAPK signaling pathway may be the common pathway that can regulate the expression of diverse receptor genes to result in insect resistance to Bt Cry toxins.

In this study, we identify a novel major mechanistic pathway that the MAPK signaling cascade *trans*-regulating differential expression of *ALP* and ABCC genes confers high-level resistance to Cry1Ac in both field-evolved and laboratory-selected strains of *P*. *xylostella*. This discovery greatly advances our comprehensive understanding of insect resistance mechanisms to Bt Cry1Ac toxin and provides new insights into how insects evolve resistance to Bt entomopathogen.

## Results

### Reduced Cry1Ac toxin binding in four resistant *P*. *xylostella* strains

**Fig 1:**
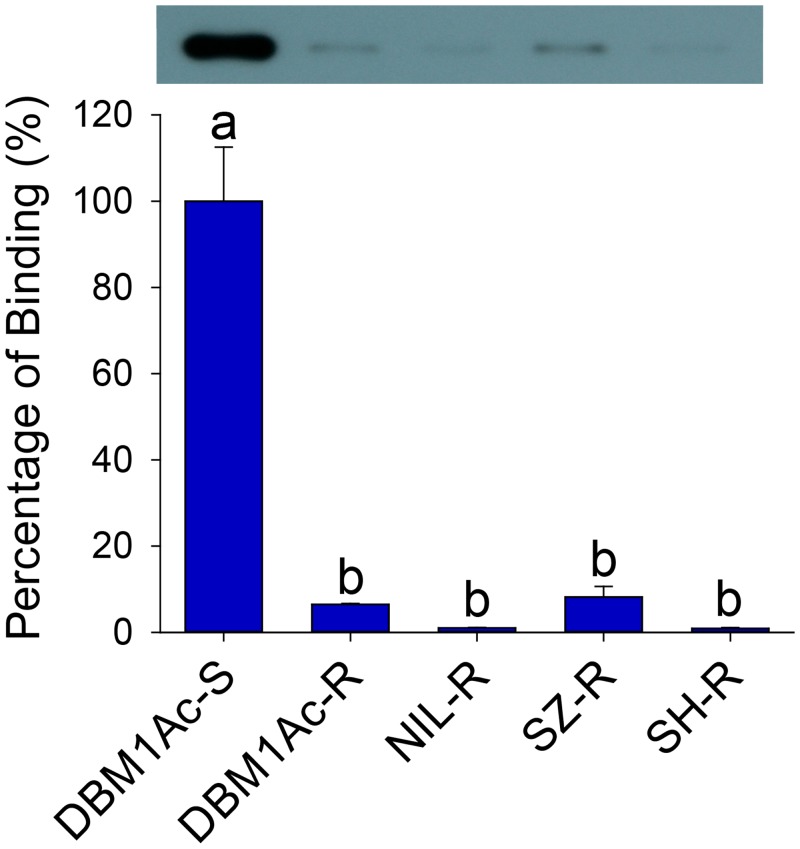
Reduced Cry1Ac binding in BBMV samples of larvae from resistant compared to susceptible *P*. *xylostella* strains. Both the detection of bound Cry1Ac by Western blotting (upper row) and quantitative estimation of binding by densitometry (graph) are presented. The percentage of binding is relative to data obtained from BBMV of the susceptible (DBM1Ac-S) strain. Error bars in each column represent standard error of the mean (SEM) from three biological replicates. Different letters on the bars indicate statistically significant differences in Cry1Ac binding among strains (P < 0.05; Holm-Sidak’s test; n = 3).

Bioassays confirmed high-level Bt resistance in diamondback moth strains originally collected from Florida (DBM1Ac-R, >3500-fold), Shenzhen (SZ-R, 458-fold Cry1Ac) and Shanghai (SH-R, 1890-fold, Bt var. *kurstaki*) (S1 Table). A fourth near-isogenic strain (NIL-R) was generated to control for variation in genetic backgrounds that may be observed between strains, and was highly resistant to both Cry1Ac (>3900-fold) and Btk (>2800-fold). Despite their diverse origins, reduced Cry1Ac toxin binding to midgut BBMV proteins was a common phenotype observed in all resistant samples compared to a Bt susceptible reference strain DBM1Ac-S (Fig 1), suggesting midgut receptor alterations as a likely resistance mechanism.

### Reduced alkaline phosphatase expression is associated with Cry1Ac and Btk resistance

**Fig 2:**
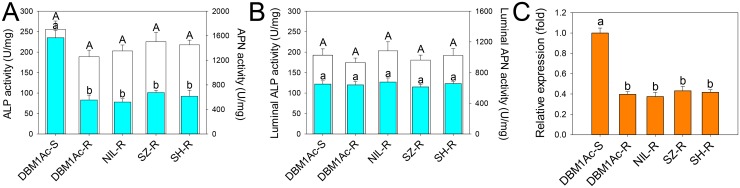
Reduced ALP levels in larval midgut samples from resistant compared to susceptible *P*. *xylostella* strains. Specific ALP (2A) and APN (2B) activity was measured in BBMV (blue bars) or midgut fluids (white bars) from the five *P*. *xylostella* strains. Error bar in each column represents standard error of the mean (SEM) from triplicate estimations using three biological replicates. Significant difference for ALP activity was detected between the susceptible strain and all resistant strains (P < 0.05; Holm-Sidak’s test; n = 3). (C) Relative expression levels of *PxmALP* as determined by qPCR in midguts of fourth-instar larvae from susceptible and resistant *P*. *xylostella* strains. Expression of the ribosomal protein *L32* gene was used as the internal reference gene to normalize datasets and calculate relative expression levels, which were calculated assigning a value of 1 to the expression levels in DBM1Ac-S samples. Data shown are the means and corresponding standard errors (SEM) from three biological replicates tested in four technical repeats. Different letters on the bars indicate statistically significant differences in gene expression among strains (P < 0.05; Holm-Sidak’s test; n = 3).

Multiple previously reported midgut receptors for Bt Cry toxins including cadherin, APN and ALP were first investigated. Recently, we have determined that the midgut cadherin is not involved in Cry1Ac resistance in all of our Cry1Ac/Btk resistant *P*. *xylsotella* strains [33]. In this study, we further detected that reduced Cry1Ac toxin binding was significantly associated with reduced ALP enzymatic activity in BBMV from larvae of all resistant strains (*P* < 0.05; Holm-Sidak’s test; n = 3), while APN activity did not differ (Fig 2A). In contrast, we did not detect significant differences in ALP or APN enzymatic activity when comparing gut luminal contents (Fig 2B), supporting a reduction of membrane-bound ALP (mALP) might be responsible for reduced toxin binding, but not soluble ALP (sALP).

As ALP activity was significantly reduced in resistant strains, we cloned the full length cDNA of a novel *mALP* gene (*PxmALP*, GenBank accession no. KC841472) from the DBM1Ac-S larval midgut tissue (S2 Table). The deduced PxmALP protein sequence displays typical structure features of a mALP protein (S1 Fig), and GenBank database search with the PxmALP protein sequence detected high identity to mALPs in diverse insect species. Phylogenetic analysis (S2 Fig) showed that PxmALP is clearly grouped into the same cluster of a clade containing many other lepidopteran mALPs reportedly involved in Bt resistance, suggesting the PxmALP may be a functional Cry1Ac toxin receptor as other lepidopteran mALPs [34,35].

Full length *PxmALP* sequencing using larval midgut cDNA from four resistant *P*. *xylostella* strains didn’t identify any constant non-synonymous substitutions or indels, suggesting *PxmALP* mutations are not linked with Cry1Ac resistance. However, qPCR analysis confirmed a significant reduction (>50%) in *PxmALP* expression in larvae from all the resistant strain (Fig 2C), which is also reflected by our RNA-Seq transcriptome profiling data [36] (S3 Table). Moreover, the reduced expression level of *PxmALP* gene is congruent with reduced ALP activity in the resistant strains.

### PxmALP is a functional Cry1Ac receptor in *P*. *xylostella* larvae

**Fig 3:**
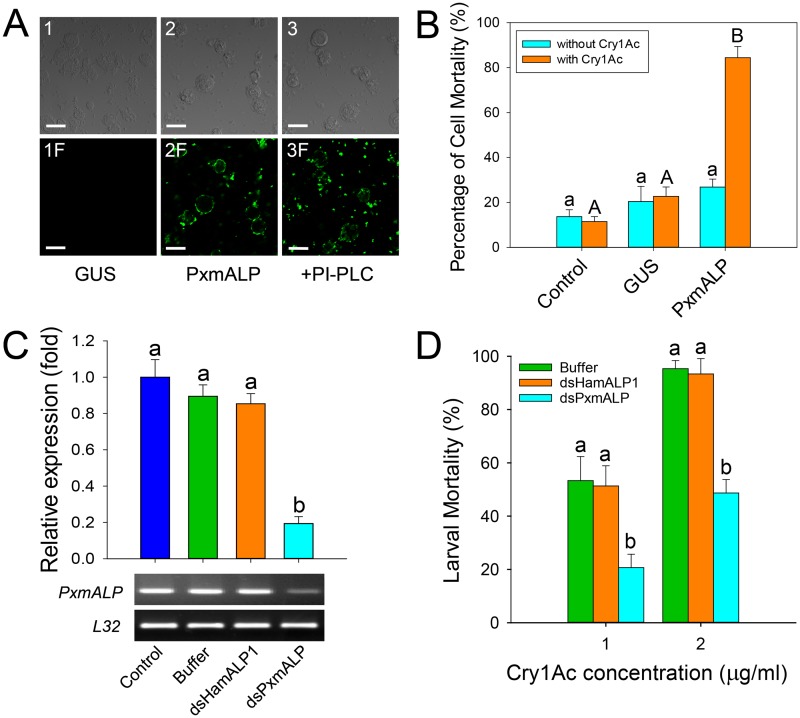
Heterologous expression and silencing of *PxmALP* have obvious effects on susceptibility to Cry1Ac. (A) Detection of Cry1Ac binding to Sf9 cells expressing PxmALP by immunolocalization. Panels 1 to 3 are differential interference contrast (DIC) microscopy views, while panels 1F to 3F represent the corresponding fluorescence microscopy images. Cells in 3 and 3F were PxmALP-expressing cells treated with PI-PLC before incubation with Cry1Ac. Bound Cry1Ac is detected as green fluorescence in panels 2F and 3F. The scale bar is 20 μm. (B) Susceptibility to Cry1Ac toxin (100 μg/ml) in control, GUS-expressing (GUS) and PxmALP-expressing (PxmALP) Sf9 cells. White bars represent mean mortality in the absence of Cry1Ac, gray bars present mortality observed after 3h incubation with Cry1Ac. (C) Effect of injection with buffer, dsHamALP1, or dsPxmALP on the relative expression of *PxmALP* compared to non-injected *P*. *xylostella* larvae (Control). Both quantitative and qualitative (lower, agarose gel of PCR products) are shown. (D) Susceptibility to two concentrations of Cry1Ac protoxin in *P*. *xylostella* larvae injected with buffer, or dsRNA targeting *HamALP1* (dsHamALP1) or *PxmALP* (dsPxmALP). Data presented in all the figures are the means and standard errors (SEM) from three biological replicates measured in triplicate. Different letters among the same treatment indicate significant differences (*P* < 0.05; Holm-Sidak’s test; n = 3).

To determine whether PxmALP can serve as a functional Cry1Ac receptor in *P*. *xylostella*, heterologous expression of recombinant PxmALP was conducted in *Spodoptera frugiperda* Sf9 cell culture, and we detected it by Western blotting and ALP activity assays using cells transfected with an empty bacmid or a bacmid containing the *Arabidopsis thaliana* β-glucuronidase (*GUS*) gene as controls (S3A Fig). Localization of PxmALP to the surface of transfected cells was demonstrated by releasing GPI-anchored proteins through cleavage with phosphatidylinositol-specific phospholipase C (PI-PLC) and detecting most of the recombinant ALP activity in the supernatant (S3B Fig). When expressed on the surface of the Sf9 cells, PxmALP bound Cry1Ac toxin as detected by confocal fluorescence microscopy (Fig 3A) and ELISA assays (S3C Fig), while no Cry1Ac toxin binding was detected in untransfected or cells expressing the *GUS* gene. As expected from the GPI-attachment to the membrane of the recombinant PxmALP, release of PxmALP from the surface of Sf9 cells by PI-PLC treatment resulted in Cry1Ac toxin binding being localized to aggregates in the media, probably containing the released PxmALP, rather than to the surface of the Sf9 cells (Fig 3A, compare panels 2F and 3F). Moreover, binding of Cry1Ac toxin to the Sf9 cells expressing PxmALP was conducive to cytotoxicity (Fig 3B), while cell viability was unaffected in untransfected and Sf9 cells expressing the *GUS* gene.

To further test PxmALP as functional Cry1Ac receptor, we silenced *PxmALP* gene expression and detected larval susceptibility to Cry1Ac protoxin. Both dsRNA concentration and timing of silencing were optimized in preliminary experiments (S4 Fig). As negative controls, we used non-injected or buffer-injected larvae, while to control for unintended off-target effects, we used larvae injected with dsRNA targeting the PxmALP ortholog in *Helicoverpa armigera* (HamALP1, GenBank accession no. EU729322.1). Sequence similarity in the dsRNA fragments targeting *PxmALP* or *HamALP1* reached to about 56%, but no consensus motifs were longer than 19 bp to avoid possible off-target effects [37]. Microinjection of dsRNA targeting an internal region of *PxmALP* (nucleotides 510 to 883) resulted in about 80% reduction in expression levels compared to controls 48 h post-injection, whereas no significant changes in expression were detected when injecting dsRNA targeting *HamALP1* (Fig 3C). Subsequent bioassays at 48 h post-injection for 72 h demonstrated that *PxmALP* silencing resulted in significantly decreased larval susceptibility to Cry1Ac protoxin (P < 0.05; Holm-Sidak’s test; n = 3) compared to controls (Fig 3D). Specifically, about 55% mortality was observed in control larvae treated with 1.0 μg/ml of Cry1Ac, while only 19% mortality was observed in larvae injected with dsPxmALP (mortality in non-injected larvae fed control diet was < 5%). When the toxin concentration was increased to 2.0 μg/ml, 95% mortality was observed in controls while only 48% mortality was detected in larvae injected with dsPxmALP.

### Assembly and candidate gene determination in the *BtR-1* resistance locus

**Fig 4:**
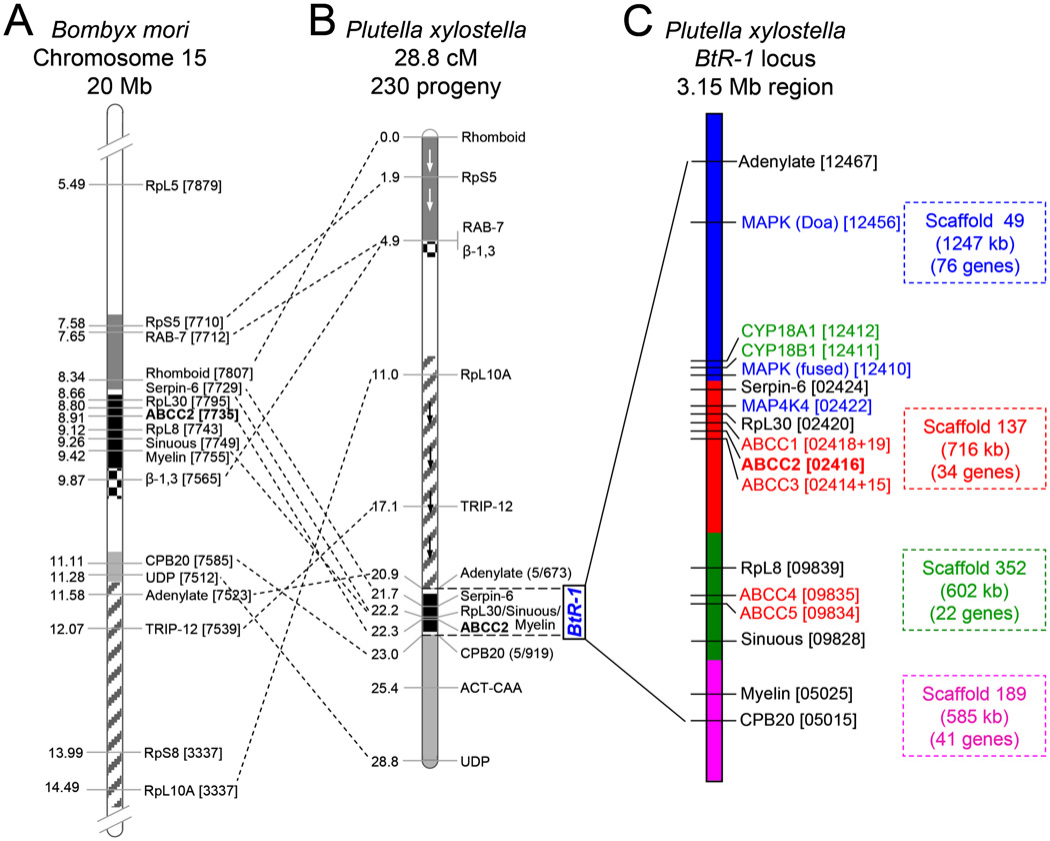
Assembly of the chromosomal region of the *BtR-1* resistance locus in *P*. *xylostella*. (A) Schematic diagram of partially sequenced chromosome 15 from *B*. *mori*. (B) *P*. *xylostella* linkage map according to Baxter et al. [10]. Blocks of common genes are shaded and arrows depict inverted orientation in *P*. *xylostella*. The dotted lines show multiple chromosomal rearrangements, while maintaining genetic synteny. (C) Detailed genetic makeup of the *BtR-1* resistance locus. The four *P*. *xylostella* genome scaffolds in *BtR-1* are distinguished by color and their information is also listed at the right side. The seven mapped genes were marked out in black (except *PxABCC2* in red), all the five *PxABCC* genes in *BtR-1* are denoted in red, three genes in the MAPK signaling pathway are labeled in blue, and two P450 genes are in green. *B*. *mori* and *P*. *xylostella* gene identifiers refer to the final four or five numbers (note underlining) of the Gene ID corresponding to each genome database (e.g., BGIBMGA007879-TA or Px012467).

While resistance to Cry1Ac in the DBM1Ac-R (previously called Cry1Ac-R) and NO-QA *P*. *xylostella* strains maps to the same *BtR-1* resistance locus [38], the *PxmALP* gene is located on a separate chromosome [26]. We assembled the approximately 3.15 Mb chromosome region representing *BtR-1* locus (Fig 4) assisted by linkage mapping data [10], genomic data of *B*. *mori* [39] and *P*. *xylostella* [40], and the genetic synteny between *P*. *xylostella* and *B*. *mori*. The *BtR-1* locus contains four *P*. *xylostella* genome scaffolds and more than 130 annotated genes (S4 Table). Presence of seven known genetic mapping marker genes [10] were confirmed and ten candidate resistance genes, including two P450 genes (*CYP*18A*1* and *CYP18B1*), five ABCC genes (ABCC1-5) and three genes involved in the MAPK signaling pathway (two MAPK genes and one MAP4K gene), were identified (Fig 4). Considering previous reports suggesting their involvement in Bt Cry toxins intoxication [9,29], we focused our subsequent work on potential alterations in the ABCC and MAPK genes in *BtR-1*.

### Resistance to Cry1Ac and alterations in five ABCC genes within the *BtR-1* resistance locus

Previous study showed that a 30-bp deletion in exon 20 of *ABCC2* gene is linked to Cry1Ac resistance in the NO-QAGE strain of *P*. *xylostella* [10], however, we did not detect any indels or constant non-synonymous substitutions in this region in our resistant strains, which led us to further study all the five ABCC genes in the *BtR-1* resistance locus. We assembled the full-length coding sequences of PxABCC1-5 by *in silico* analysis, PCR cloning and sequencing, and the bona fide full-length cDNA sequences of these five ABCC genes (GenBank accession nos. KM245560–KM245564) as they were incorrectly annotated in the draft *P*. *xylostella* genome. Cloning and comparison of the full-length PxABCC1-5 cDNA sequences from midgut pools of susceptible or resistant larvae detected sequence variations in the region encompassing exons 4 to 11 of PxABCC1-3, which did not result in changes in the number or size of amplified bands in PCR assays targeting *PxABCC1* (S5A Fig, left figure), but led to additional amplicons observed for *PxABCC2* and *PxABCC3* (S5B and S5C Fig, top figures). Sequencing of these amplicons from each strain allowed us to detect one *PxABCC1* isoform, eleven *PxABCC2* isoforms and four *PxABCC3* isoforms, probably resulting from alternative splicing of the PxABCC1-3 mRNA precursor (S6 Fig). Some of the identified alternative splicing isoforms contained premature stop codons in the transmembrane domain (TMD) or the subsequent nucleotide binding domain 1 (NBD1), which would result in truncated and possibly non-functional proteins. However, their relative distribution was similar (S5A Fig, right figure; S5B and S5C Fig, bottom figures) among untreated individual susceptible (DBM1Ac-S) larvae and larvae of the NIL-R resistant strain surviving exposure to an extremely high dose of Cry1Ac protoxin (10000 μg/ml, causes 100% mortality in DBM1Ac-S larvae), supporting no associations between ABCC isoforms in that region and resistance to Cry1Ac. The lack of large inversions or deletions was also tested and confirmed using one-step amplification of the full-length PxABCC1-5 cDNA followed by nested PCR with overlapping primer sets (S5–S9 Tables).

**Fig 5:**
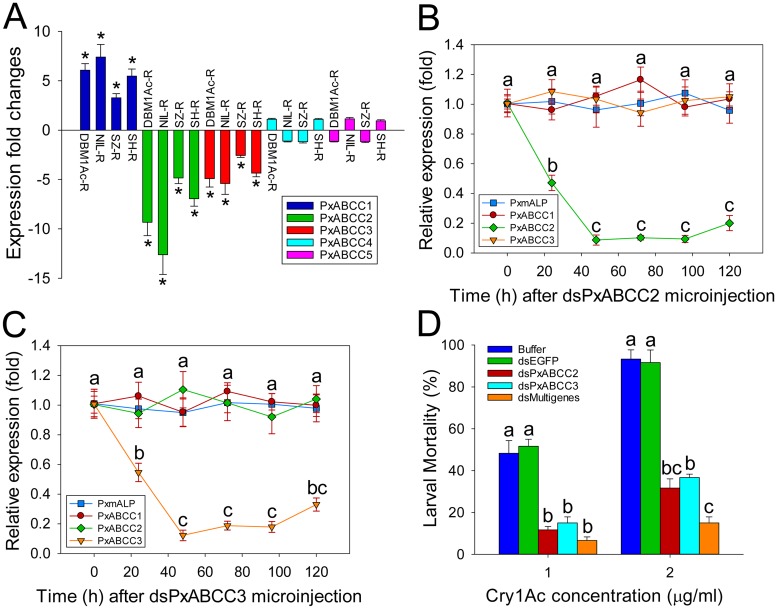
Functional analysis of *PxABCC* genes in the *BtR-1* resistance locus. (A) Gene expression difference (in fold) between larvae from a susceptible (DBM1Ac-S) and four resistant *P*. *xylostella* strains. Expression of the ribosomal protein *L32* gene was used as the internal standard, and relative expression levels for each gene were calculated assigning a value of 1 to the expression level for each gene in DBM1Ac-S. Positive and negative values indicate increased and decreased mRNA levels, respectively, in resistant compared to susceptible larvae. Data shown are the means and corresponding standard errors (SEM) from three biological replicates tested in four technical repeats. Asterisks indicate significant differences among samples (P < 0.05; Holm-Sidak’s test; n = 3). Silencing of *PxABCC2* (B) or *PxABCC3* (C) by microinjection of specific dsRNA (dsPxABCC2 or dsPxABCC3) is expressed relative to expression levels for each gene at time 0 h, which was assigned a relative value of 1. Data shown are the means and corresponding standard errors (SEM) from three biological replicates tested in four technical repeats. Different letters on the bars indicate statistically significant differences in gene expression among different time periods (P < 0.05; Holm-Sidak’s test; n = 3). (D) Susceptibility to two concentrations of Cry1Ac protoxin in susceptible *P*. *xylostella* larvae injected with buffer or dsRNA targeting *EGFP* (dsEGFP), *PxABCC2* (dsPxABCC2), *PxABCC3* (dsPxABCC3) or a multiple gene mixture (dsPxmALP+dsPxABCC2+dsPxABCC3). Data presented are the means and standard errors (SEM) from three biological replicates measured in triplicate. Different letters among the same treatment indicate significant differences (*P* < 0.05; Holm-Sidak’s test; n = 3).

Since no association between mutations in PxABCC genes and resistance was observed, we subsequent compared levels of expression for all five ABCC genes in the *BtR-1* locus in susceptible and resistant strains using qPCR (Fig 5A). These analyses revealed that three of the five ABCC genes (PxABCC1-3) showed significant differences in gene expression between susceptible and resistant strains, whereas no obvious expression alteration for *PxABCC4* or *PxABCC5* was found. Interestingly, while *PxABCC2* and *PxABCC3* were significantly down-regulated, *PxABCC1* was dramatically up-regulated in all the resistant compared to susceptible *P*. *xylostella* strains (P < 0.05; Holm-Sidak’s test; n = 3). These observations (except *PxABCC2* differences) were also detected after re-analyzing of our RNA-Seq transcriptome profiling data [36] (S3 Table).

The observed association between differential PxABCC gene expression and resistance led us to investigate the relationship among the three PxABCC genes. Protein sequence analysis showed that these genes share typical structural features of ABCC family members, including two transmembrane domains (TMDs) and two nucleotide binding domains (NBDs). The genomic structure showed that these three PxABCC genes display high protein sequence similarity (about 59%), extremely similar exon size and number, and the same intron phase (S10 Table; see also S7A Fig), which indicating they are paralogs derived from an ancient gene duplication event. Phylogenetic analysis showed that the *P*. *xylostella* ABCC1-3 genes share high sequence identity with homologs from *Spodoptera exigua* (S7B Fig), which have been recently proved to be involved in resistance to Cry1 toxins [12].

To determine the effect of *PxABCC2* and *PxABCC3* down-regulation on susceptibility to Cry1Ac toxin, we silenced their expression by RNAi and tested susceptibility (LC_50_ and LC_90_) in silenced larvae. Expression levels for both genes were significantly reduced at 24 h after dsRNA injection, with lowest expression levels detected after 48 h and lasting at least 72 h in both cases (Fig 5B and 5C). Silencing was specific to each ABCC gene and did not affect *PxmALP* expression levels (Fig 5B and 5C). Bioassays performed at 48 h post-injection for 72 h showed a marked decrease in susceptibility to Cry1Ac toxin in both dsPxABCC2- and dsPxABCC3-treated larvae compared to the buffer- or dsEGFP-injected larvae. Moreover, silencing of multiple genes simultaneously (combinational RNAi) by injection of a combination of dsRNAs targeting *PxmALP*, *PxABCC2* and *PxABCC3* (dsMultigenes) resulted in a comparatively higher reduction in susceptibility to Cry1Ac (P < 0.05; Holm-Sidak’s test; n = 3) (Fig 5D). Specifically, about 50% mortality was observed in control larvae treated with 1.0 μg/ml of Cry1Ac (LC_50_ value), while only 12%, 15% and 6% mortality was observed in larvae injected with dsPxABCC2, dsPxABCC3 and dsMultigenes, respectively (mortality in non-injected larvae fed control diet was <5%). When using 2.0 μg/ml (LC_90_ value), 92% mortality was observed in controls while only 32%, 37% and 15% mortality was detected in larvae injected with dsPxABCC2, dsPxABCC3 and dsMultigenes. All three silencing treatments had significant effects (LSD test; P < 0.05; n = 3) on fitness components, including decreased pupation rate, reduced pupal weight, shortened pupal time and lower eclosion rate when compared to control treatments, and no differences (LSD test; P > 0.05; n = 3) were detected among control treatments (S11 Table).

### Testing linkage of reduced *PxmALP*, *PxABCC2* and *PxABCC3* expression with resistance to Cry1Ac

We further performed genetic linkage analysis to test non-cosegregation of alternative ABCC1-3 gene splicing isoforms or cosegregation of differentially altered expression of the *PxmALP* and PxABCC1-3 genes with resistance to Cry1Ac toxin in the NIL-R strain. Reciprocal F2 backcross families from crossing the near-isogenic NIL-R (resistant) and DBM1Ac-S (susceptible) strains were generated and selected on cabbage with or without a lethal dose of Cry1Ac protoxin (S8 Fig). Comparison of distribution and sequencing of PxABCC1-3 cDNA isoforms among individual larvae from backcross families exposed or not to Cry1Ac toxin demonstrated no association between PxABCC1-3 isoforms and resistance to Cry1Ac (S9 Fig).

**Fig 6:**
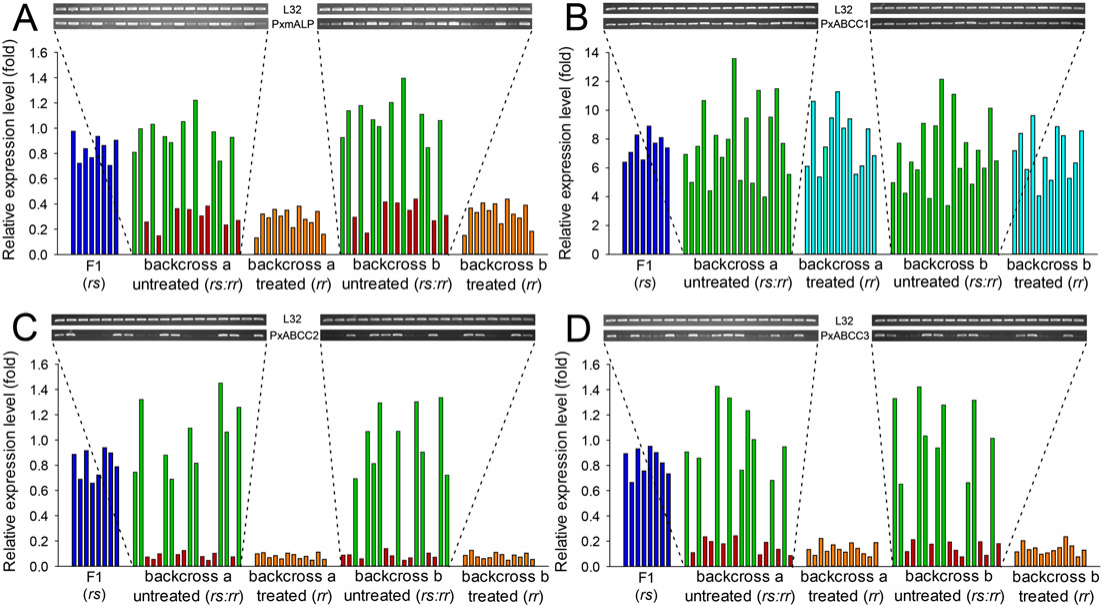
Linkage of resistance to Cry1Ac to reduced expression of *PxmALP*, *PxABCC1*, *PxABCC2* or *PxABCC3* genes in the NIL-R strain. Expression levels (in fold) for the F1, Cry1Ac-selected and non-selected backcross families are relative to levels in the susceptible (DBM1Ac-S) strain. Corresponding intensity of amplified transcript bands for the *PxmALP* (A), *PxABCC1* (B), *PxABCC2* (C), *PxABCC3* (D) and the internal standard ribosomal protein *L32* gene are shown in the upper pictures. Both results using larvae of the subfamily generated from backcrossing a female from F1 and a male from the NIL-R strain (backcross family a) and using larvae of the subfamily generated from backcrossing a male from F1 and a female from the NIL-R strain (backcross family b) are shown.

Quantification of *PxmALP* and PxABCC1-3 expression levels in individual larval midguts from backcross families not exposed to Cry1Ac selection showed two distinct groups (Fig 6). One group demonstrated significantly reduced expression levels of *PxmALP* (< 0.4-fold), *PxABCC2* (< 0.15-fold) and *PxABCC3* (< 0.25-fold), while the other group displayed expression levels similar to larvae from the susceptible parental strain (DBM1Ac-S) or the F1 generation from NIL-R × DBM1Ac-S crosses (Fig 6A, 6C and 6D). The ratio between the numbers of individuals in each group, 8:10, 9:9 and 9:9 in backcross family a and 9:9, 9:9 and 10:9 in backcross family b, for the *PxmALP*, *PxABCC2* and *PxABCC3* genes, respectively, were statistically validated to follow the 1:1 random assortment ratio (*P* > 0.10 or *P* = 1.0; χ^2^ test). Rearing of neonates from both backcross families on Cry1Ac resulted in about 50% mortality (55% backcross family a, 47.5% in family b), consistent with the expected Mendelian inheritance of the recessive resistance trait. All the surviving larvae in both backcross families had reduced *PxmALP* (< 0.4-fold), *PxABCC2* (< 0.15-fold) and *PxABCC3* (< 0.25-fold) expression levels compared to larvae from the DBM1Ac-S strain or the F1 generation, demonstrating tight linkage (cosegregation) with resistance to Cry1Ac in NIL-R (P < 0.001, χ^2^ test). However, the expression levels for the *PxABCC1* gene in both Cry1Ac-selected and non-selected larvae were similarly up-regulated (4- to 14-fold) compared to susceptible larvae (Fig 6B). Although the unselected backcross individuals exhibited two distinct groups with differing *PxmALP*, *PxABCC2* and *PxABCC3* expression levels, both groups of larvae had similar *PxABCC1* expression levels (t test, P > 0.10), supporting no correlation between down-regulation of *PxmALP*, *PxABCC2*, or *PxABCC3* and up-regulation of *PxABCC1* expression in the larvae.

### Silencing of *PxMAP4K4* gene in *BtR-1* locus recovers expression of *PxmALP* and three PxABCC genes thereby restoring Cry1Ac susceptibility in NIL-R larvae

Analysis of the *BtR-1* locus found three genes (two MAPK genes and one MAP4K gene) involved in MAPK signaling pathways (S4 Table; see also Fig 4). Unlike the two MAPK genes, the *PxMAP4K4* gene locates extremely close to the three PxABCC genes within the core *BtR-1* locus and shows perfect genetic synteny between *P*. *xylostella* and *B*. *mori* (S4 Table; see also S10 Fig). Using specific primers (S12 Table), we cloned and corrected the full-length cDNA sequence of the incorrectly annotated *PxMAP4K4* gene in the *P*. *xylostella* genome (DBM-DB, Gene ID Px002422), the bona fide full-length cDNA sequence has been deposited in the GenBank database (accession no. KM507871). Sequence alignment of the deduced amino acid sequences showed conserved N-terminal kinase (Serine/threonine kinase catalytic domain, STKc) and the C-terminal regulatory (citron/NIK homology domain, CNH) domains of *PxMAP4K4* gene in homologs from different species (S11 Fig).

**Fig 7:**
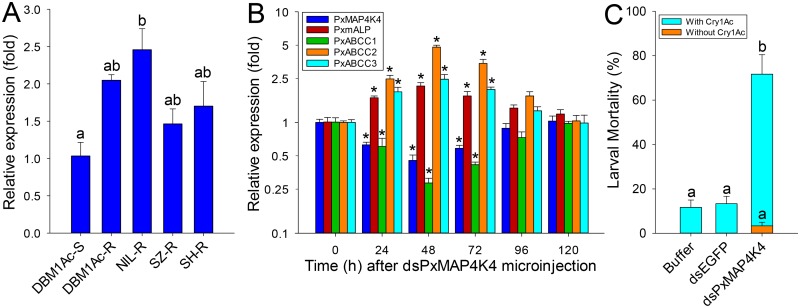
Detection of *PxMAP4K4* gene expression and effect of its silencing on expression of *PxmALP* and PxABCC genes and susceptibility to Cry1Ac. (A) Relative expression levels of *PxMAP4K4* as determined by qPCR in midguts of fourth-instar larvae from susceptible (DBM1Ac-S) and unselected resistant *P*. *xylostella* strains. Expression of the ribosomal protein *L32* gene was used as internal reference to normalize datasets and calculate relative expression levels, which were calculated assigning a value of 1 to the expression levels in DBM1Ac-S samples. Data shown are the means and corresponding standard errors (SEM) from three biological replicates tested in four technical repeats. Different letters on the bars indicate statistically significant differences in gene expression among strains (P < 0.05; Holm-Sidak’s test; n = 3). (B) Silencing of the *PxMAP4K4* gene by microinjection of specific dsRNA (dsPxMAP4K4) *in vivo* and detecting the effect on resistance genes including *PxmALP*, *PxABCC1*, *PxABCC2* and *PxABCC3* at different periods. The expression level of each gene at 0 h after dsPxMAP4K4 injection was assigned a value of 1 for comparison. Data shown are the means and corresponding standard errors (SEM) from three biological replicates tested in four technical repeats. Asterisks (*) indicate significant difference among periods for each gene (P < 0.05; Holm-Sidak’s test; n = 3). (C) Susceptibility to Cry1Ac protoxin (LC_10_, 1000 μg/ml) in resistant NIL-R larvae injected with buffer or dsRNA targeting *EGFP* (dsEGFP) or *PxMAP4K4* (dsPxMAP4K4). Data presented are the means and standard errors (SEM) from three biological replicates measured in triplicate. Different letters indicate significant differences (*P* < 0.05; Holm-Sidak’s test; n = 3).

Comparisons of *PxMAP4K4* expression levels between susceptible and resistant strains by qPCR showed that this gene was constitutively up-regulated in larvae from all resistant strains compared to the susceptible strain (Fig 7A). Moreover, toxin induction assays showed that the expression level of *PxMAP4K4* was significantly increased (P < 0.05; Holm-Sidak’s test; n = 3) in the susceptible strain DBM1Ac-S but didn’t alter (P > 0.05; Holm-Sidak’s test; n = 3) in the resistant strain NIL-R when treated with respective median lethal concentration of Cry1Ac in both strains, suggesting that the high expression levels of *PxMAP4K*4 in resistant strain was constitutive rather than induced (S12 Fig).

To confirm the significance of this observation, we silenced *PxMAP4K4* expression by RNAi in resistant NIL-R larvae and tested larval susceptibility to Cry1Ac post-RNAi. Microinjection of dsRNA targeting the CNH domain region of the *PxMAP4K4* mRNA resulted in about 55% reduction (0.45-fold) in expression levels at 48 h post-injection, with expression returning to control levels at 120 h post-injection (Fig 7B). Correspondingly, the expression levels of *PxmALP*, *PxABCC2* and *PxABCC3* were significantly increased by 2.1-, 4.8- and 2.5-fold at 48 h post-injection, whereas the expression level of *PxABCC1* was dramatically reduced 0.28-fold (77% reduction) at this time point (Fig 7B). Subsequent bioassays performed at 48 h post-injection for 72 h demonstrated that silencing of *PxMAP4K4* gene expression resulted in a significant increase in larval susceptibility to Cry1Ac protoxin (P < 0.05; Holm-Sidak’s test; n = 3) when compared to the buffer- or dsEGFP-injected larvae (Fig 7C). Specifically, about 3% and 12% mortality was observed in respective control larvae untreated or treated with 1000 μg/ml of Cry1Ac (LC_10_ value), while approximately 72% mortality was observed in larvae injected with dsPxMAP4K4, and mortality in dsPxMAP4K4-treated larvae not exposed to toxin was < 4%.

## Discussion

**Fig 8:**
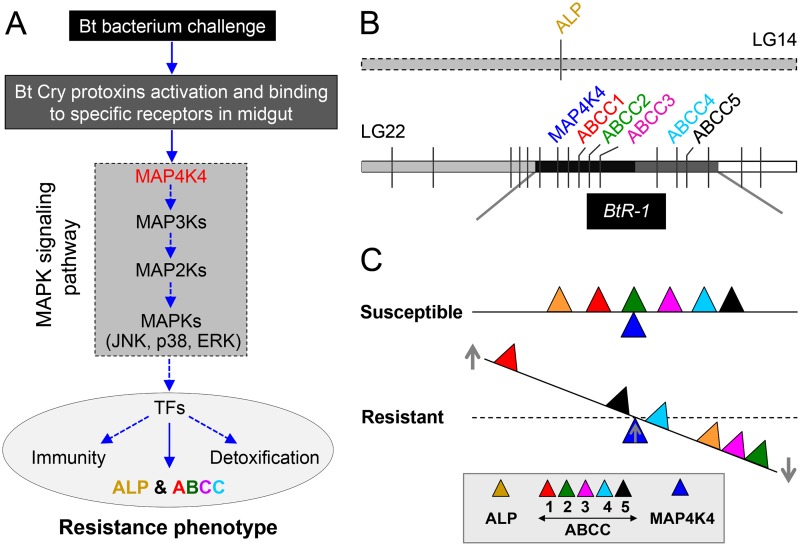
Schematic drawing depicting a novel Cry1Ac resistance mechanism in *P*. *xylostella*. (A) Cry protoxins produced by Bt bacterium are activated by midgut proteases and then bind to specific midgut receptors to form ionic pores leading to insect death. In resistant insects, over-activation of the MAPK signaling pathway (highlighted in the dashed box, *MAP4K4* is an important upstream regulator) serves to alter the expression of different genes associated with the resistance phenotype, including receptor genes involved in Bt resistance probably through the regulation of diverse transcription factors (TFs). Blue arrows indicate recruitment, activation, or production. (B) Diagram of the physical location of the genes involved in Cry1Ac resistance in *P*. *xylostella*. *ALP* is located in the linkage group 14 and outside the *BtR-1* resistance locus, whereas other genes are located within the *BtR-1* locus in the linkage group 22 [26]. (C) *MAP4K4*-mediated interplay between *ALP* and *ABCC* genes among *P*. *xylostella* strains with different susceptibility to Cry1Ac toxins. Based on this study, we propose that *MAP4K4* serves as a general switch modulating the differential expression of *ALP* and multiple ABCC genes in *P*. *xylostella*. While it is not linked to Cry1Ac resistance, up-regulation of *ABCC1* in resistant *P*. *xylostella* larvae may represent potential compensatory effects that can help reduce fitness costs due to *ABCC2* and *ABCC3* down-regulation in resistant larvae. However, the data here does not strongly support the conclusion that expression levels of *ABCC4* and *ABCC5* are significantly regulated by *MAP4K4* and changes in their expression obviously affect the development of Cry1Ac resistance. Up- and downward arrows in the figure represent the up- and down-regulation of genes, respectively, in Cry1Ac-resistant and susceptible *P*. *xylostella* strains. The distance remove from the fulcrum point reflects the qualitative magnitude of gene regulations.

Field insect populations can develop resistance to entomopathogens used as biopesticides, such as *B*. *thuringiensis* (Bt), limiting their potential efficacy for pest management. Multiple Cry1A midgut receptors have been reported in Lepidoptera, which should theoretically make resistance evolution difficult, however, genetic analysis has commonly shown resistance to be a single autosomal locus [41]. Data in this study provides a comprehensive mechanistic description of resistance to Cry1Ac and a Btk biopesticide in larvae from diverse *P*. *xylostella* strains. Although previous reports supported that mutations in the *PxABCC2* gene localized to the *BtR-1* locus are responsible for resistance to Cry1Ac in *P*. *xylostella* [10], we did not detect any mutations in the *PxABCC2* or other PxABCC genes in *BtR-1* associated with resistance in any of our tested strains. In contrast, our findings clearly support that differential expression of a midgut membrane-bound alkaline phosphatase (*PxmALP*) gene and a suite of PxABCC genes (including *PxABCC2*) is associated with high levels of resistance to Cry1Ac and Btk in *P*. *xylostella*. This is the first report showing that expression alterations, not gene mutations, of *ABCC2* and other ABCC genes can be involved in insect Bt resistance. More importantly, for the first time, we identify a transcriptionally-activated upstream gene in the MAPK signaling pathway (*PxMAP4K4*) within the *BtR-1* locus can *trans*-regulate differential altered expression of the *PxmALP* and PxABCC genes in *BtR-1* to result in Cry1Ac resistance. This novel molecular mechanism of Cry1Ac resistance in *P*. *xylostella* is summarized in Fig 8.

Resistance to Cry1Ac in our field-evolved strain DBM1Ac-R [42] and the near isogenic strain NIL-R [43] fits the “Mode 1” type characterized by high levels of resistance to at least one Cry1A toxin, recessive inheritance, reduced binding of at least one Cry1A toxin, and lack of cross-resistance to Cry1C toxin [44]. Reduction in Cry1Ac binding is associated with “Mode 1” type resistance in nearly all cases of field-evolved *P*. *xylostella* resistance [7]. This observation, coupled with the Cry toxin binding site model developed for *P*. *xylostella* [45] and our Cry1Ac toxin binding data, clearly suggested that “Mode 1” resistance in our strains was due to alterations in at least one Cry1A toxin receptor. While a number of putative Cry receptors have been proposed [8], only alterations in cadherin, APN, ALP and ABCC2 genes have been found to associate with high resistance to Cry1Ac in Lepidoptera [6]. Dramatically reduced ALP enzymatic activity in BBMV samples of resistant *P*. *xylostella* larvae prompted us to clone the full-length cDNA of this gene for further functional studies. Sequence analysis showed that the cloned *PxmALP* is identical to the partial *ALP1* gene sequence (GenBank accession no. EF579960, a partial genomic sequence including an intron). In agreement with Cry1Ac resistance not genetically mapping to *ALP1* [26], we did not detect any mutations in *PxmALP* associated with resistance among our *P*. *xylostella* strains, however, the *PxmALP* expression levels were significantly reduced. This observation is also supported by our prior reports demonstrating down-regulation of *ALP*1 in the DBM1Ac-R (Cry1Ac-R in that report) strain [46] and a more detailed analysis of our recent RNA-Seq survey (S3 Table) [36]. In agreement with these observations, current data support ALPs as relevant Cry1A toxin binding proteins [47,48]. Importantly, altered ALP levels were described as associated with “Mode 1” type resistance to Cry1Ac in lepidopteran hosts [17,18], yet no mechanistic or linkage evidence were provided. Based on the functional and linkage data here, we propose a model for “Mode 1” resistance in *P*. *xylostella* in which PxmALP serves as a “lethal receptor” for Cry1Ac toxin. Down-regulation of *PxmALP* expression in resistant larvae results in reduced toxin binding to the midgut cells and survival. Our definition of PxmALP as a “lethal receptor” is based on its effectiveness as Cry1Ac receptor in cell assays, and the similar susceptibility in heterozygous and homozygous susceptible larvae, as previously suggested in Cry1Ac-resistant *H*. *virescens* [49].

Previous complementation tests have suggested that the same resistance locus (*BtR-1*) containing the mutant *PxABCC2* gene [10] is responsible for resistance to Cry1Ac in *P*. *xylostella* strains from the continental US (PEN from Pennsylvania, SC1 from South Carolina and DBM1Ac-R originally collected from Florida), Hawaii (NO-QA and NO-QAGE), and China (SZBT) [22,25,38]. Since that the *PxmALP* gene is not located in the *BtR-1* locus [26], our first hypothesis to reconcile the available data on *P*. *xylostella* resistance was that mutations or altered expression of *PxABCC2* or other PxABCC genes in *BtR-1* could result directly or indirectly in reduced expression of *PxmALP*. In support of this observation, resistance to a Bt pesticide in *S*. *exigua* [12,50] and to Cry1Ac in *T*. *ni* [10,24] was linked to mutations in *ABCC2* and a concomitant down-regulation of an *APN* gene. However, unlike previous reports [10], we did not detect any mutations in *PxABCC2* associated with resistance. Instead, we detected alternative splicing of ABCC subfamily genes, as previously reported in other insects [51–53]. Some of the splicing isoforms can lead to truncated ABCC proteins, which in heterozygotes would be masked by the susceptible allele, as previously proposed for cadherin gene [49]. Thus, these alternative splicing isoforms may represent a natural system to generate recessive gene mutation pools for Bt resistance selection. Intriguingly, alternative ABCC splicing was limited to the exon 4–11 region, suggesting that this cDNA region is more prone to allow mutations. This mechanism would explain rapid appearance of field-evolved resistance to Bt sprays in *P*. *xylostella*.

Although gene mutations of PxABCC genes were excluded, our further study corroborated differential expression alterations of these PxABCC genes were associated with Cry1Ac intoxication in *P*. *xylsotella*. However, our RNAi results showed that silencing of *PxABCC2* or *PxABCC3* genes did not affect *PxmALP* expression, which suggests PxABCC genes can’t regulate the expression level of *PxmALP* gene. Then, an independent role of PxABCC and PxmALP genes in Cry1Ac susceptibility is suggested as our second hypothesis. In support of this observation, similar effects on Cry1Ac susceptibility were detected when silencing PxABCC or *PxmALP* genes separately, and the comparatively higher reduction in Cry1Ac susceptibility was observed after performing combinatorial RNAi to simultaneous silencing of *PxmALP*, *PxABCC2* and *PxABCC3* genes. Therefore, it is plausible to postulate that an uncharacterized *trans*-regulatory gene in the *BtR-1* locus could potentially control differential altered expression of both PxABCC and *PxmALP* resistance genes. Not incidentally, a similar hypothesis was proposed to explain allelic expression alteration of *ABCC2* and *APN3* genes in *B*. *mori* [54], and APN down-regulation in Cry1Ac-resistant *T*. *ni* [24] and Cry1Ab-resistant *Ostrinia nubilalis* [55].

While genes modulating ALP and ABCC expression in insects have not been reported, genes in the MAPK signaling pathway have been shown to modulate mammalian ALP [56–58] and human ABCC gene expression [59–62]. The MAPK signaling pathway can be activated as a defensive response to Bt Cry toxins [27–30]. Consequently, it is plausible that altered *PxmALP* and PxABCC gene expression in resistant *P*. *xylostella* may result from a primary enhanced defensive response involving activation of a *trans*-acting gene in the MAPK signaling pathway located in the *BtR-1* locus. As expected, we found three MAPK genes in *BtR-1* and identified the *PxMAP4K4* gene in proximity to the three ABCC genes (S10 Fig). Of particular note, homologs of this *PxMAP4K4* gene in mammals, *Caenorhabditis elegans*, and *Drosophila* are all upstream components of the MAPK signaling pathway and play important physiological roles in these species [63–65]. Accordingly, we found that the expression level of this gene can be induced in the susceptible strain when challenged by low concentration of Cry1Ac toxin, and functional data in this study demonstrated that this gene is constitutively transcriptionally-activated in resistant larvae to *trans*-regulate *PxmALP* and PxABCC expression levels thereby dramatically affecting Cry1Ac susceptibility, which finally attests to the involvement of the MAPK signaling pathway in *P*. *xylostella* Cry1Ac resistance. Since that cadherin can mediate the intracellular MAPK signaling pathway in mammal or insect cells [32,66,67], and considering that alteration of cadherin gene expression is associated with resistance to Cry toxins in several other lepidopteran insects [68,69], it will be very interesting to examine the possible feedback regulation of MAPK signaling in cadherin gene expression regulation. Moreover, since diverse upstream cytokines in the MAPK signaling pathway can regulate expression of APN genes in mammals [70,71], and considering that alteration of APN gene expression is associated with resistance to Cry toxins in several other lepidopteran insects [24,50,54,55,72], it will be very interesting to examine the possible involvement of MAPK signaling in APN gene expression regulation. Recently, we have found that down-regulation of a novel ABC transporter gene (*PxABCG1* or *Pxwhite*) possibly *trans*-regulated by the MAPK signaling pathway can also be involved in *P*. *xylostella* Cry1Ac resistance, suggesting the MAPK signaling pathway may *trans*-regulate numerous ABC transporters from different subfamilies [73]. Therefore, it is plausible that this novel *trans*-regulatory mechanism might be a common regulation event of diverse Bt receptor genes in all of these cases.

Duplication or amplification of functional genes is thought to be a major driving force for adaptive evolution of insect response to environmental stress [74] and development of insecticide resistance [75,76]. Whole genomic analyses support that the ABC transporter superfamily has undergone apparent gene duplication in the *P*. *xylostella* genome [40], and this duplication may allow for functional redundancy. Based on their extremely similar genomic structure and high sequence similarity, it is highly possible that the PxABCC1-3 genes in *BtR-1* locus may have been generated through gene duplication and share similar functions. Considering that *PxABCC1* gene up-regulation was not linked to Cry1Ac resistance, we speculate that *PxABCC1* may have lost the ability to bind Cry1Ac toxin but retained substrate transport function to functionally rescue the reduced *PxABCC2* and *PxABCC3* phenotype in resistant larvae. Likewise, transcriptionally-activated MAPK signaling induced by Cry5B intoxication in *C*. *elegans* up-regulated a target cation efflux transporter gene (*ttm-1*) possibly involved in removing cytotoxic cations generated by toxin-induced pore formation [27]. This phenomenon would also resemble up-regulation of *APN*6 in Cry1Ac-resistant *T*. *ni* with down-regulated *APN1* expression [24]. Since silencing of *PxABCC2* and *PxABCC3* genes result in obvious fitness costs in *P*. *xylostella* larvae, rescue of ABCC gene function in resistant *P*. *xylostella* by *PxABCC1* would also help explain lack of fitness costs in the DBM1Ac-R strain [77]. These data suggest that functional redundancy in ABCC genes can reduce fitness costs and thus increase the probability of resistance evolution in the field, which may threaten the continued effectiveness of Bt sprays/Bt crops and the currently adopted refuge strategy. In this case, we should attach great importance to this observation and perform continuous field monitor of insect resistance in such form.

In summary, the present study shows that alteration in expression of multiple putative Cry1Ac receptors is linked to “Mode 1” type resistance in *P*. *xylostella*, and that this altered gene expression is *trans*-regulated by the MAPK signaling pathway. Although the data presented do not directly address the participation of additional elements and the full repertoire of the MAPK signaling pathway, they do provide strong evidence for an important role of this signaling pathway in insect susceptibility to Cry1Ac toxin. Further work is needed to identify additional toxin receptor genes (e.g. cadherin and APN) that may also be controlled by this pathway and other MAPK genes or downstream transcription factors involved in Bt resistance in insects. The present data deepens our understanding of how insect target cells counter Cry intoxication through functionally sophisticated intracellular responses to result in Bt resistance. Moreover, the identified pivotal genes and their expression regulation mechanism responsible for resistance to Cry toxins in this study are critical for sensitive and efficient monitoring and management practices to delay field-evolved insect resistance to Bt pesticides and Bt crops.

## Materials and Methods

### Insect strains

The susceptible DBM1Ac-S and resistant DBM1Ac-R (previously referred to as Cry1Ac-R) strains of *P*. *xylostella* were originally provided by Drs. J. Z. Zhao and A. Shelton (Cornell University, USA) in 2003. The DBM1Ac-R strain originated from insects with field-evolved resistance to Javelin (Bt var. *kurstaki*) from Loxahatchee (Florida, USA) [78] that were crossed with the DBM1Ac-S (Geneva 88) strain (originated from Geneva, NY, USA) and further selected with Cry1Ac-expressing broccoli [79]. Resistance to Cry1Ac in DBM1Ac-R is autosomal, incompletely recessive and mostly monogenic [77]. The SZ-R (previously referred to as T2-R) and SH-R strains were originated from moths collected in China at Shenzhen (2003) and Shanghai (2005), respectively. The SZ-R strain was generated by selection in the laboratory with Cry1Ac while the SH-R strain was selected with a Bt var. *kurstaki* (Btk) formulation (WP with potency of 16000 IU/mg, provided by Bt Research and Development Centre, Agriculture Science Academy of Hubei Province, China). The DBM1Ac-S strain was kept unselected while the DBM1Ac-R and SZ-R strains have been kept under constant selection with a Cry1Ac protoxin solution killing 50–70% of the larvae sprayed on cabbage leaves. The near-isogenic NIL-R strain has been generated at the time this study was carried out and has been described elsewhere [43]. For this work, all strains were reared on JingFeng No.1 cabbage (*Brassica oleracea* var. *capitata*) without exposure to any Bt toxins or chemical pesticides at 25°C, 65% RH and 16D:8L photoperiod. Adults were fed with a 10% sucrose solution.

### Cry1Ac toxin preparation and bioassay

The Cry1Ac protoxin was extracted and purified from Bt var. *kurstaki* strain HD-73 as previously described [80]. Both purified Cry1Ac protoxin and trypsin-activated toxin were quantified by densitometry as described elsewhere [81].

Toxicity of Cry1Ac toxin or Btk formulation in 72 h bioassays with larvae from five different strains of *P*. *xylostella* using a leaf-dip method as described elsewhere [33]. Ten third instar *P*. *xylostella* larvae were tested for each of seven toxin concentrations and bioassays replicated four times. Mortality data were corrected using Abbott’s formula [82] and experiments with control mortality exceeding 10% were discarded and repeated. The LC_50_ values were calculated by Probit analysis [83].

### Midgut dissection and sample preparation

Fourth-instar larval midguts (about 2000) from each *P*. *xylostella* strain were dissected in cold MET buffer [17 mM Tris–HCl (pH 7.5), 5 mM EGTA, 300 mM mannitol] plus protease inhibitors (1mM PMSF). Midgut brush border membrane vesicles (BBMV) were prepared as described elsewhere [84]. Purified BBMV proteins were quantified using the method of Bradford [85] with bovine serum albumin (BSA) as standard, and then flash frozen and kept in aliquots at -80°C until used. Between 5–8 fold enrichment in specific APN activity using L-leucine-*p*-nitroanilide (Sigma) was detected when comparing to initial midgut homogenates.

Midgut luminal contents were obtained as described elsewhere [18] by homogenization of pools of dissected midguts of actively feeding fourth-instar *P*. *xylostella* larvae with an electric pestle in a 1.5 ml centrifuge tube containing 100 μl of phosphate-buffered saline (PBS) buffer (137 mM NaCl, 2.7 mM KCl, 10 mM Na_2_HPO_4_, 2.0 mM KH_2_PO_4_, pH 7.4). Homogenates were vigorously vortexed and centrifuged at 4°C (10 min at 16000×*g*). Supernatants were used for subsequent enzymatic assay measurements or flash frozen and kept in aliquots at -80°C until used.

### Enzymatic activity assays

Specific APN and ALP activity assays were performed using L-leucine-*p*-nitroanilide and *p*-nitrophenyl phosphate disodium (*p*NPP) as substrates, respectively, as described elsewhere [86]. Enzymatic activity was detected as the changes in optical density (OD) at 405 nm for 5 min at room temperature in a SpectraMax M2^*e*^ (Molecular Devices) microplate reader. One enzymatic unit was defined as the amount of enzyme that would catalyze production of the chromogenic product from specific substrate per min and mg of BBMV protein at 37°C. Data shown are the means from triplicate measurements from three independent BBMV preparations and were analyzed for significance with a one-way ANOVA using Holm-Sidak’s tests (overall significance level = 0.05) with the SPSS Statistics (ver. 17.0) software (SPSS Inc.).

### *BtR-1* resistance locus assembly

The seven mapped genes in Baxter et al. [10] were used as markers to find *P*. *xylostella* genome scaffolds within the chromosome region of the *BtR-1* resistance locus in the Diamondback moth Genome Database (DBM-DB, http://iae.fafu.edu.cn/DBM), and their locations determined the final jointed pattern of all *P*. *xylostella* genome scaffolds. Homologs of the genes within this locus between *P*. *xylostella* and *B*. *mori* were identified through Blastp searches in each genome database. The detailed genetic makeup of the *BtR-1* resistance locus is listed in S4 Table.

### RNA extraction and cDNA synthesis

Fourth-instar larvae from different *P*. *xylostella* strains were anesthetized on ice and the midgut tissues were immediately dissected in RNase-free water containing 0.7% NaCl. Total RNA was extracted from single or pool of these dissected midguts using TRIzol reagent (Invitrogen) according to different experiments. Integrity of the RNA was determined using 1% TBE agarose gel electrophoresis, and then quantified by a NanoDrop 2000c spectrophotometer (Thermo Fisher Scientific Inc.). For gene cloning, the first-strand cDNA was prepared using 5 μg of total RNA with the PrimeScript Ⅱ 1st strand cDNA Synthesis Kit (TaKaRa) following manufacturer’s recommendations. For qPCR analysis, the first-strand cDNA was prepared using 1 μg of total RNA with the PrimeScript RT kit (containing gDNA Eraser, Perfect Real Time) (TaKaRa) following manufacturer’s recommendations. The synthesized first-strand cDNA was immediately stored at -20°C until used.

### Gene cloning and sequencing

For *PxmALP* cloning, degenerate primers (S2 Table) were designed to target two highly conserved regions among selected lepidopteran mALP sequences (*Bombyx mori*, BAB62745; *Helicoverpa armigera*, ACF40806; *Heliothis virescens*, ACP39712; and *Ostrinia furnacalis*, AEM43806). The PCR parameters were as follows: one cycle of 94°C for 3 min; 35 cycles of 94°C for 30 s, 59°C for 45 s and 72°C for 1 min; a final cycle of 72°C for 10 min. The generated 536 bp midgut cDNA fragment was sequenced and used in Rapid Amplification of cDNA Ends (RACE) to obtain the full length ALP cDNA with 3′-Full RACE Core Set Ver.2.0 (TaKaRa) and SMARTer RACE cDNA Amplification (Clontech) kits following manufacturer’s protocols. Once a full-length cDNA for *PxmALP* was obtained the full coding sequence was validated by PCR amplification. Large-scale sequencing and comparing of the full-length midgut *PxmALP* was performed among all the susceptible and resistant *P*. *xylostella* strains. The full length midgut *PxmALP* cDNA sequence has been deposited in the GenBank (accession no. KC841472).

To first detect the reported *PxABCC2* mutation (30 bp deletion in Exon 20) correlated with Bt resistance in *P*. *xylostella* strain NO-QAGE [10], one specific primer pair covering this region was designed based on the published partial *PxABCC2* cDNA sequence (GenBank accession no. JN030490) (S6 Table). The PCR amplification of all the cDNA sequences of five ABCC genes in the *BtR-1* resistance locus was performed using the strategy described elsewhere [87]. We first *in silico* assembled and corrected the full-length sequences of ABCC1-5 based on their putative coding sequences in the DBM-DB database (http://iae.fafu.edu.cn/DBM, Gene ID: Px002418+19, Px002416, Px002414+15, Px009835 and Px009834) and their unigenes in our *P*. *xylostella* transcriptome database [88] (S3 Table), then their cDNA was amplified by designing full-length primer pairs with four or five overlapping fragments (S5–S9 Table). The full length ABCC1-5 cDNA sequences have been deposited in GenBank (accession nos. KM245560–KM245564). In addition, the occurrence of large inversions or deletions was tested in a second amplification strategy by amplifying the whole cDNA sequence with specific full-length primers (S5–S9 Table) in a first step, followed by nested PCR amplification using the same full-length primer pairs with four or five overlapping fragments as described above. Amplicons were sequenced and the distribution of alternatively spliced transcripts of ABCC1-3 was compared among samples from untreated and larvae surviving Cry1Ac exposure. Using the same strategy as for ABCC genes, we cloned and obtained the full-length cDNA sequence of the *PxMAP4K4* gene (GenBank accession no. KM507871).

The PCR reactions (25 μl total volume) contained 18.5 μl of double-distilled H_2_O (ddH_2_O), 2.5 μl of 10×LA Taq or Ex Taq Buffer, 2 μl of dNTP Mix, 5 μM of each specific primer, 1 μl of first-strand cDNA template, and 0.25 μl LA Taq HS or Ex Taq HS polymerase (TaKaRa). Reactions (35 cycles) were then performed in an S1000 or C1000 Thermal Cycler PCR system (BioRad) with the following parameters: one cycle of 94°C for 6 min; 35 cycles of 94°C for 30 s, 50–59°C for 45 s and 72°C for 5 min; a final cycle of 72°C for 15 min. The nested PCR parameters were as follows: one cycle of 94°C for 6 min; 35 cycles of 94°C for 30 s, 59°C (*PxABCC2* and *PxMAP4K4*)/54°C (other four PxABCC genes) for 45 s and 72°C for 2 min; a final cycle of 72°C for 10 min.

All the cloning primers for each gene were designed in the Primer Premier 5.0 software (Premier Biosoft). Amplicons of the expected size were excised from 1.5–2.5% agarose gels, purified using the Gel Mini Purification Kit (Generay), and subcloned into the pEASY-T1 (Transgen) or pMD18-T vectors (TaKaRa) before transformation into *Escherichia coli* TOP10 competent cells (Transgen) for sequencing.

### *In silico* gene sequence analysis and phylogenetic tree construction

Gene sequence assembling, multiple sequence alignment and exon-intron analysis were carried out with DNAMAN 7.0 (Lynnon BioSoft). The open reading frame of the target nucleotide sequence is found by the ORF Finder tool at NCBI website (http://www.ncbi.nlm.nih.gov/gorf/gorf.html). The nucleotide sequence-similarity analyses were performed through BLAST tool at NCBI website (http://blast.ncbi.nlm.nih.gov/). The deduced protein sequence was obtained by an ExPASy translate tool Translate (http://web.expasy.org/translate/) from the Swiss Institute of Bioinformatics. The N-terminal signal peptide was determined using the SignalP 4.0 server (http://www.cbs.dtu.dk/services/SignalP/). The transmembrane region and membrane topology was analyzed by the TOPCONS online software (http://topcons.cbr.su.se/). Protein specific motif was searched and analyzed using the Myhits software (http://myhits.isbsib.ch/cgi-bin/motif_scan), the Prosite software (http://www.expasy.ch/prosite/) and CDD (conserved domain database) at NCBI. Two GPI modification site prediction servers (big-PI Predictor: http://mendel.imp.ac.at/sat/gpi/gpi_server.html and GPI-SOM: http://gpi.unibe.ch/) were used to predict the GPI-anchor signal sequence and GPI anchoring site. Presence of N- and O-glycosylation sites on the predicted protein sequence were tested using the NetNGlyc 1.0 (http://www.cbs.dtu.dk/services/NetNGlyc/) and NetOGlyc 4.0 server (http://www.cbs.dtu.dk/services/NetOGlyc/), respectively.

Protein sequences of the ALP and ABCC genes used for phylogenetic analyses were extracted from different databases: GenBank (http://www.ncbi.nlm.nih.gov/), SilkDB (http://silkworm.genomics.org.cn/), DBM-DB (http://iae.fafu.edu.cn/DBM) and Manduca Base (http://agripestbase.org/manduca/), and sequence alignment was carried out after eliminating vast redundant ALP or ABCC sequences. All the selected insect ALP and ABCC amino acid sequences were subjected to analysis through Clustal W alignment using Molecular Evolutionary Genetic Analysis software version 5.0 (MEGA 5) [89], then the phylogenetic tree was constructed using the neighbor-joining (NJ) method with “p-distance” as amino acid substitution model, “pairwise deletion” as gaps/missing data treatment and 1000 bootstrap replications.

### Quantitative PCR (qPCR) analysis

Gene-specific primers to the *PxmALP* gene were selected and used in PCR reactions (25 μl) containing 11.95 μl of ddH_2_O, 11.25 μl of 2.5×SYBR Green MasterMix (TIANGEN), 4 μM of each specific primer, and 1 μl of first-strand cDNA template. The qPCR program included an initial denaturation for 6 min at 94°C followed by 40 cycles of denaturation at 94°C for 30 s, annealing for 30 s at 61°C, and extension for 35 s at 72°C. Gene-specific primers for *PxABCC* and *PxMAP4K4* genes were designed in the cDNA regions without alternative splicing and used in PCR reactions (25 μl) containing 9.5 μl of ddH_2_O, 12.5 μl of 2×SuperReal PreMix Plus (TIANGEN), 7.5 μM of each specific primer, 1 μl of first-strand cDNA template and 0.5 μl 50×ROX Reference Dye (TIANGEN). The qPCR program included an initial denaturation for 15 min at 95°C followed by 40 cycles of denaturation at 95°C for 15 s, annealing for 30 s at 53°C (*PxABCC2*)/55°C (other four PxABCC genes)/63°C (*PxMAP4K4*), and extension for 32 s at 72°C. For melting curve analysis, an automatic dissociation step cycle was added. Reactions were performed in an ABI 7500 Real-Time PCR system (Applied Biosystems) with data collection at stage 2, step 3 in each cycle of the PCR reaction. Amplification efficiencies were calculated from the dissociation curve of quadruplicate replicates using five 2-fold serial dilutions (1:1, 1:2, 1:4, 1:8, and 1:16). Only results with single peaks in melting curve analyses, 95–100% primer amplification efficiencies, and >0.95 correlation coefficients were used for subsequent data analysis. Negative control reactions included ddH_2_O instead of cDNA template, which resulted in no amplified products. The amplified fragments were sequenced to confirm that potential expression differences were not due to sequence mutations in the targeted genes. Relative quantification was performed using the 2^-ΔΔCt^ method [90] and normalized to the ribosomal protein *L32* gene (GenBank accession no. AB180441) as validated elsewhere [40,91]. Four technical replicates and three biological replicates were used for each treatment. One-way ANOVA with Holm-Sidak’s tests (overall significance level = 0.05) were used to determine the significant statistical difference between treatments.

### Heterologous expression of PxmALP in Sf9 cell cultures

The Bac-to-Bac Baculovirus Expression System (Invitrogen) was used to express the recombinant PxmALP protein in *Spodoptera frugiperda* Sf9 cell cultures. The full-length *PxmALP* cDNA was cloned and amplified by high fidelity PCR with specific primers (S2 Table). Amplicons were purified, subcloned and sequenced as described above. Recombinant plasmids with correct insertion were verified by endonuclease digestion, PCR and sequencing. The verified positive clone was digested with *Eco*RI and *Xba*I for 3 h and then ligated into the pFastBac TH B donor plasmid vector to generate the recombinant pFastBac HT B-PxmALP bacmid. The recombinant plasmids (pFastBac HT B-PxmALP) were then transformed into DH10Bac competent cells (Invitrogen) and positive recombinant bacmid DNAs were detected by antibiotic selection and confirmed by PCR amplification.

For heterologous expression, transfections were performed in sterile six-well plates (Costar). Briefly, Sf9 cultures (8×10^5^ cells/well) with >97% viability were cultured in Grace’s insect medium supplemented with 10% fetal bovine serum and transfected with 1 μg of pFastBac HT B-PxmALP in Cellfectin II Reagent (Invitrogen) following manufacturer’s instructions. Cells were incubated at 27°C until the viral infection was clear (3 days post-infection) and then the P1 viral stock was harvested by centrifugation at 480×*g* for 5 min at room temperature. The viral titer was determined using absolute quantification with standard curve by qPCR. The optimized viral stock with multiplicity of infection (MOI) of 0.1 was used to infect 2.0×10^6^ Sf9 cells/well, and the supernatant at 72 h post-infection representing the P2 viral stock was collected and used to infect Sf9 cells (2.0×10^6^ cells/well) at an optimized high MOI value (3–5). Non-infected cells and Sf9 cells infected with either an empty bacmid or a bacmid containing the *Arabidopsis thaliana* β-glucuronidase (*GUS*) gene (pFastBac-GUS) were used as controls.

Transfected Sf9 cell pellets were harvested 3 days post-infection, washed three times with PBS buffer (pH 7.4) and lysed using the I-PER Insect Cell Protein Extraction Reagent (Thermo Fisher Scientific Inc.) plus 1 μg/ml aprotinin (Sigma) with gentle agitation at 4°C for 10 min. After centrifugation at 15000×*g* at 4°C for 15 min, the supernatants containing recombinant proteins were quantified as described above, and stored at -80°C until used.

### Cry1Ac toxin binding assays

Binding of Cry1Ac to BBMV proteins was tested as described elsewhere [92] in 100 μl (final volume) reactions containing 10 nM Cry1Ac toxin and 10 μg *P*. *xylostella* BBMV in PBS binding buffer (PBS, pH 7.4 containing 0.1% BSA and 0.1% Tween-20). After electrophoresis with a constant current of 300 mA at 4°C for 1 h and then incubation with blocking buffer (PBS, 0.1% Tween-20, 3% BSA) for 1 h with constant shaking, bound toxin was detected with rabbit anti-Cry1Ac polyclonal antisera (1:100000 dilution) followed by goat anti-rabbit secondary antibody conjugated to horseradish peroxidase (HRP) (1:5000 dilution, CWBIO). The bound Cry1Ac was visualized using the SuperSignal West Pico (Pierce) reagent. Relative Cry1Ac binding was quantified using densitometry with the ImageJ v.1.47 software (http://rsbweb.nih.gov/ij/) with intensity in the DBM1Ac-S BBMV sample considered 100% binding. Data presented are the means and standard errors from assays using three independent BBMV experiments per strain.

Immunolocalization of Cry1Ac toxin binding to Sf9 cells expressing PxmALP was tested as previously [93] with slight modifications. Transfected cell cultures were incubated in 300 μl of PBS (pH 7.4) alone or with 1 U of phosphatidylinositol-specific phospholipase C (PI-PLC) (Invitrogen) at 4°C for 2 h with gentle agitation, and then washed thrice with PBS and incubated with Cry1Ac toxin (100 μg/ml) at 27°C for 2 h. Cultures were washed and then fixed in ice-cold 4% paraformaldehyde for 15 min. After washing and blocking with 1% BSA for 1 h at room temperature, the cells were probed sequentially with primary rabbit polyclonal anti-Cry1Ac antibody and FITC-conjugated goat anti-rabbit secondary antibody, each with 1:100 dilution and incubation for 1 h at 27°C. Finally, the cells were pipetted onto glass slides, mounted with coverslips and examined immediately under a LSM 700 confocal laser scanning microscope (Carl Zeiss) using excitation at 488 nm and 20× objective with additional zooming. Image acquisition of the controls (Non-infected Sf9 cells and GUS-infected Sf9 cells) and data processing were performed under the same conditions.

Enzyme Linked Immunosorbent Assays (ELISA) were performed as described elsewhere [47,94]. To test for Cry1Ac binding to PxmALP, 10 nM trypsin-activated Cry1Ac toxin was fixed into ELISA plates (Costar) overnight at 4°C, followed by five washes with 200 μl PBST buffer (PBS, pH 7.4; 0.05% Tween-20). The plates were then blocked by incubating with 100 μl 1% BSA at 37°C for 1.5 h, and washed five times with 200 μl PBST. After incubating with 0.5 μg of solubilized Sf9 cell culture proteins transfected with empty bacmid, expressing the GUS protein or PxmALP, bound PxmALP to Cry1Ac was detected using a 1:5000 dilution of anti-His antibody coupled to horseradish peroxidase (HRP) and subsequent 1:5000 dilution of anti-mouse antibody (CWBIO). Finally, the plates were incubated with 150 μl TMB (3,3′,5,5′-tetramethylbenzidine) Horseradish Peroxidase Color Development Solution (Beyotime), and the enzymatic reaction was stopped with 50 μl 2M H_2_SO_4_ and absorbance values (OD values) were read at 450 nm in microplate reader. As controls, wells coated with Cry1Ac but incubated without any of the three expressed proteins and revealed with the same antibodies above. The OD values of controls were all below 0.2 and subtracted from the experimental OD values. The experiments were repeated for three times using protein samples from independent batches and each with three replications.

### Cytotoxicity assays

Susceptibility to Cry1Ac in transfected Sf9 cell cultures was assessed by counting cells stained by trypan blue using an IX-71 Inverted Microscope (Olympus). Cells at 3-day post-infection were washed twice with PBS and then incubated with 100 μg/ml of Cry1Ac toxin in Sf9 cell medium. After 3 h at 27°C with gentle agitation, cells were washed once with 1 ml of PBS and then resuspended in 1 ml of a 0.4% trypan blue solution in PBS. The numbers of live (unstained) and dead (stained blue) cells in three replicates for each cell type and from three independent transfections were counted in a hemocytometer. Relative percentage mortalities were calculated using the total cell number in each replicate. Mortality data from diverse treatments were tested for significant differences using two-way analysis of variance (ANOVA) and Holm-Sidak’s multiple pairwise comparison tests (overall significance level = 0.05).

### Toxin induction assays

Toxin induction assays of the *PxMAP4K4* gene were performed using third instar larvae from the susceptible strain DBM1Ac-S and the near-isogenic resistant strain NIL-R. We selected the DBM1Ac-S or NIL-R larvae with 1 or 3500 μg/ml Cry1Ac protoxin (respective LC_50_ value in each strain) for 72h as the leaf-dip method used in bioassay, the unselected larvae from both strains were used as control groups. After 72h, larval midguts were dissected from survivors, and subsequent total RNA extraction, cDNA synthesis, qPCR analysis of the *PxMAP4K4* gene expression were as described above. Three independent experiments were conducted, and one-way ANOVA with Holm-Sidak’s tests (overall significance level = 0.05) were used to determine the significant statistical difference between control and treatment groups.

### dsRNA synthesis and RNAi assays

The expression of *PxmALP*, *PxABCC2*, *PxABCC3* and *PxMAP4K4* genes was silenced using injection of dsRNA in early 3rd instar *P*. *xylostella* larvae. Specific primers containing a T7 promoter sequence at the 5′ end to generate dsRNA targeting *PxmALP* (GenBank accession no. KC841472) and *mALP1* from *H*. *armigera* (GenBank accession no. EU729322.1), or EGFP (GenBank accession no. KC896843) were designed using the SnapDragon tool (http://www.flyrnai.org/cgi-bin/RNAi_find_primers.pl). Primers to generate dsRNA to *PxABCC2* (GenBank accession no. KM245561) and *PxABCC3* (GenBank accession no. KM245562) were designed to the specific transmembrane region lacking alternative splicing and not in the intergenic conserved nucleotide binding domain (NBD) to avoid potential off-target effects. Primers for dsRNA of *PxMAP4K4* (GenBank accession no. KM507871) were designed to the constant C-terminal CNH domain region lacking alternative splicing (S11 Fig). After amplification from *P*. *xylostella* or *H*. *armigera* total larval midgut RNA and confirmation by sequencing, the amplicons (438 bp for dsPxmALP, 538 bp for dsHamALP1, 469 bp for dsEGFP, 603 bp for dsPxABCC2, 531 bp for dsPxABCC3, and 582 bp for dsPxMAP4K4) were used as template for *in vitro* transcription reactions to generate dsRNAs using the T7 Ribomax Express RNAi System (Promega). The synthesized dsRNAs were suspended in injection buffer (10 mM Tris–HCl, pH 7.0; 1 mM EDTA), and then they were subjected to 1% agarose gel electrophoresis and quantified spectrophotometrically prior to microinjection. To increase dsRNA stability and facilitate dsRNA delivery, injection was carried out with a 1:1 volume ratio of Metafectene PRO transfection reagent (Biontex) after incubation for 20 min at 25°C. A combinatorial RNAi approach involving simultaneous knockdown of *PxmALP*, *PxABCC2* and *PxABCC3* genes was performed by mixing equal amounts (300 ng each) of the corresponding dsRNAs for microinjection of larvae from the susceptible DBM1Ac-S strain. In contrast, silencing of *PxMAP4K4* was performed in the near-isogenic Cry1Ac resistant strain NIL-R displaying increased *PxMAP4K4* expression. None of the larvae were exposed to Cry1Ac toxin before dsRNA microinjection to avoid detection of transcriptome changes due to exposure to the toxin. Optimal time to detect silencing and dsRNA amounts were optimized for *PxmALP* in preliminary experiments (S4 Fig).

Microinjection was carried out under a SZX10 microscope (Olympus). The volume of sample microinjected into each larvae was determined to result in <20% larval mortality 5 days post-injection. The Nanoliter 2000 microinjection system (World Precision Instruments Inc.) with sterilized fine glass capillary microinjection needles pulled by P-97 micropipette puller (Sutter Instrument) were used to deliver 70 nanoliters of injection buffer (containing Metafectene PRO solution) or dsRNAs (300 ng) into the hemocoel of early 3rd instar DBM1Ac-S or NIL-R *P*. *xylostella* larvae.

Larvae were starved for 6 h and anesthetized for 30 min on ice before microinjection. More than twenty or fifty larvae were injected for each treatment and three independent experiments performed. Injected larvae were allowed to recover for about 3 h at room temperature and then returned to normal rearing conditions for the subsequent qPCR assays to determine gene silencing and bioassays.

Effectiveness of RNAi was tested by qPCR 0–120 h post-injection using cDNA prepared from isolated total midgut RNA as described above. Leaf-dip bioassays were performed for 72 h using larvae at 48 h after dsRNA injection and Cry1Ac protoxin concentrations representing approximately the LC_50_ (1 μg**/**ml) and LC_90_ (2 μg**/**ml) values for non-injected DBM1Ac-S larvae and LC_10_ (1000 μg**/**ml) values for non-injected NIL-R larvae. Bioassays were performed with forty larvae per RNAi treatment and toxin concentration, and each bioassay replicated three times. Mortality in control treatments was below 5% and bioassay data processing was as described above. One-way or two-way ANOVA with Holm-Sidak’s tests (overall significance level = 0.05) were used to determine the significant statistical difference between qPCR and bioassay treatments, respectively.

Effects of RNAi on fitness costs were analyzed by comparing biological parameters, including pupation percentage, pupal weight, pupation duration and eclosion percentage. Larvae injected with buffer containing Metafectene PRO transfection reagent were used as a negative control. All the larvae used in the test were fed on fresh cabbage leaves without exposure to Cry1Ac toxin. Each treatment was replicated three times with ten larvae per replicate. Least squared difference (LSD) tests (overall significance level = 0.05) were used to determine statistical significance of differences in biological parameters between control and treated groups.

### Genetic linkage analysis

The near-isogenic NIL-R (resistant) and DBM1Ac-S (susceptible) strains were used for genetic linkage analysis as described elsewhere [24]. A single-pair cross was prepared between a male from the NIL-R and a female from the DBM1Ac-S strain to generate an F1 progeny. A diagnostic Cry1Ac toxin dose killing 100% of the F1 (heterozygous) larvae was determined in bioassays as described above. Reciprocal crosses between an F1 and NIL-R moths were made to generate two backcross families (S8 Fig). The progenies from each backcross family (total of 40 larvae per family) were reared on control (cabbage) or experimental (cabbage with 20 μg/ml of Cry1Ac toxin) diets.

Purified RNA from single backcross family individuals surviving no treatment or exposure to Cry1Ac was used for cDNA synthesis. Linkage between the existence of multiple PxABCC gene isoforms or differential alteration of *PxmALP* and PxABCC gene expression and resistance to Cry1Ac was tested using PCR amplification and qPCR conditions as described above.

## Supporting Information

S1 Fig Nucleotide and deduced amino acid sequence of *PxmALP* gene. Numbers on the right indicate the nucleotide (upper) and amino acid (lower) position of *PxmALP* gene (GenBank accession no. KC841472). The start codon (ATG), stop codon (TAG) and putative polyadenylation signal (AATAAA) of the cDNA sequence are highlighted in green. The signal peptide is underlined in blue. The thirteen specific amino acid residues involved in substrate or metal ligand binding are enclosed by brown diamonds. The GPI- anchoring site is marked by a blue triangle. The three predicted N-glycosylation sites are inside green square boxes, and the seven putative O-glycosylation sites are red-circled. The active phosphatase site and functional residues are marked with a black character or shaded in light blue, respectively. The fragment conserved in the *ALP1* gene (GenBank accession no. EF579960) exon is shown underlined in pink.
(TIF)

S2 Fig Phylogenetic relationship of alkaline phosphatase genes in five insect orders. A neighbor-joining (NJ) consensus tree was generated by ClustalW alignment of the ALP amino acid sequences from different insect species available in the GenBank or Diamondback moth Genome (http://iae.fafu.edu.cn/DBM/index.php) databases using MEGA 5.0 software [89]. The bootstrap values expressed as percentages of 1000 replications are shown at branch points, values lower than 10% were hidden in the tree. GenBank accession numbers or Gene ID are displayed within the tree and indicated in parentheses. The PxmALP protein is marked by a blue solid diamond. The ALP genes from different orders are shown in different colors. Abbreviations: **1. Lepidoptera** (**Bmo**, *Bombyx mori*; **Bma**, *Bombyx mandarina*; **Ha**, *Helicoverpa armigera*; **Hv**, *Heliothis virescens*; **Of**, *Ostrinia furnacalis*; **Tn**, *Trichoplusia ni*; **Se**, *Spodoptera exigua*; **Dp**, *Danaus plexippus*; **Ds**, *Diatraea saccharalis*; **Sl**, *Spodoptera litura*; **Px**, *Plutella xylostella*); **2. Diptera** (**Dm**, *Drosophila melanogaster*; **Aa**, *Aedes aegypti*; **Aga**, *Anopheles gambiae*; **Cq**, *Culex quinquefasciatus*); **3. Coleoptera** (**Tc**, *Tribolium castaneum*); **4. Hemiptera** (**Agl**, *Aphis glycines*; **Nl**, *Nilaparvata lugens*; **Ap**, *Acyrthosiphon pisum*); **5. Hymenoptera** (**Pp**, *Pteromalus puparum*; **Nv**, *Nasonia vitripennis*; **Am**, *Apis mellifera*; **Ae**, *Acromyrmex echinatior*; **Hs**, *Harpegnathos saltator*; **Cf**, *Camponotus floridanus*; **Mr**, *Megachile rotundata*; **Af**, *Apis florae*; **Bi**, *Bombus impatiens*; **Bt**, *Bombus terrestris*).
(TIF)

S3 Fig Detection of heterologous expression of *PxmALP* in Sf9 cells and Cry1Ac binding to the recombinant protein. (A) Detection of *PxmALP* expression in Sf9 cells by Western blotting with antisera to a 6×His tag (Anti-His) at the N-terminal end of the recombinant PxmALP protein and by colorimetric detection of ALP activity (ALP activity) as described elsewhere [86]. In all panels, lanes 1 are cells transfected with empty vector; lanes 2, cells transfected to produce the GUS protein; lanes 3, cells transfected to express *PxmALP*. Left panel (Total protein) is a Coomassie blue-stained gel to demonstrate equal lane loading. A lane from a gel containing BBMV proteins (20 μg) of *P*. *xylostella* (Px BBMV) is shown as reference of PxmALP in larval midgut. The loading order of the lanes in the ALP activity gel has been altered to facilitate comparison between panels. (B) Confirmation that recombinant PxmALP is GPI-anchored to the Sf9 cell surface. Cultures of Sf9 cells expressing PxmALP were treated with buffer (-PI-PLC) or PI-PLC (+PI-PLC) as described in Materials and Methods, and then solubilized proteins recovered in supernatants after centrifugation. Specific ALP activities in cell pellets (PE) and solubilized proteins present in supernatants (SU) are shown. Data shown are the means and standard errors (SEM) from triplicate determinations using three independent biological samples (P < 0.05, Holm-Sidak’s test; n = 3). Different letters within a treatment denote significant differences between samples. (C) ELISA assay testing binding of Cry1Ac toxin to recombinant PxmALP expressed in Sf9 cell cultures. Binding of recombinant protein from non-transfected cells (Control), cells expressing the *GUS* gene (GUS), or cells expressing PxmALP (PxmALP) to Cry1Ac on the ELISA plate was detected using anti-His antibody. Bars denote the mean and standard error (SEM) values from three biological replicates, each tested at least in triplicate. Different letters denote significant differences (P < 0.05, Holm-Sidak’s test; n = 3).
(TIF)

S4 Fig Determination of dsRNA concentration and time post-injection for optimum *PxmALP* silencing by RNAi. (A) Silencing of the target *PxmALP* gene by injection of *P*. *xylostella* larvae with 300 ng of dsRNA (dsPxmALP) was detected at different times post-injection by qPCR. As a control, larvae were injected with buffer or dsRNA targeting the *mALP1* gene from *H*. *armigera* (dsHamALP1). Quantification of *PxmALP* expression levels in reference to the ribosomal protein *L32* gene for larvae injected with dsHamALP1 or dsPxmALP is shown, and the template for each reaction was cDNA prepared from pools of 10 larvae. Each point represents the mean and standard error (SEM) from three biological replicates performed in quadruplicate. Different letters represent significant differences in expression levels between treatments (*P* < 0.05; Holm-Sidak’s test; n = 3). (B) Quantification by qPCR of *PxmALP* expression levels at 48 h post-injection when larvae were injected with increasing dsRNA concentrations (70 nl final volume). For each qPCR reaction pools of cDNA from 10 larvae were used. Expression levels for ribosomal protein *L32* gene were used as reference gene and to confirm the integrity of the cDNA. Expression levels for *PxmALP* in non-injected larvae (control) were used as the maximum relative expression levels for comparisons among treatments. Bars represent the means and standard errors (SEM) from three biological replicates performed in quadruplicate, with different letters indicating significant differences (*P* < 0.05; Holm-Sidak’s test; n = 3).
(TIF)

S5 Fig Sequence variations in the *PxABCC1*, *PxABCC2* and *PxABCC3* cDNA regions within exons 4–11 among susceptible and resistant strains of *P*. *xylostella*. In (A), (B) and (C), lane 1: DBM1Ac-S; lane 2: DBM1Ac-R; lane 3: NIL-R; lane 4: SZ-R; lane 5: SH-R; lane M: molecular size markers. (A) Detection of an expected 916 bp amplicon based on the GenBank sequence of *PxABCC1* (accession no. KM245560) in midgut samples from susceptible and resistant strains (left figure) and single midgut cDNA samples from untreated 4th instar larvae of DBM1Ac-S (lanes 1–6) and larvae of the NIL-R strain surviving exposure to 10000 μg**/**ml of Cry1Ac protoxin (right figure, lanes 7–12). Although the amplicons from the alternatively spliced isoform in this case appears with the same size as the wild type, sequencing results confirmed that amplicons in lanes 2, 4, 5, 7, 9, 10, 11 represent alternative splicing isoforms. (B) Detection of an expected 915 bp amplicon (top left figure) or three additional amplicons corresponding to *PxABCC2* mutant isoforms ranging from 600–1000 bp in size (top right figure) based on the GenBank sequence (accession no. KM245562) in midgut samples from susceptible and resistant strains. Also shown is detection of *PxABCC2* isoforms by PCR using single midgut cDNA samples from untreated 4th instar larvae of DBM1Ac-S (lanes 1–6) and larvae of the NIL-R strain surviving exposure to 10000 μg**/**ml of Cry1Ac protoxin (bottom figure, lanes 7–12). (C) Detection of an expected 885 bp amplicon (top left figure) or additional amplicons corresponding to *PxABCC3* mutant isoforms ranging from 500–1000 bp in size (top right figure) based on the GenBank sequence (accession no. KM245562) in midgut samples from susceptible and resistant strains. Also shown is detection of *PxABCC3* isoforms by PCR using single midgut cDNA samples from untreated 4th instar larvae of DBM1Ac-S (lanes 1–6) and larvae of the NIL-R strain surviving exposure to 10000 μg**/**ml of Cry1Ac protoxin (bottom figure, lanes 7–12). Amplicons in (A) and (B) were separated by 2.5% agarose gel electrophoresis, while amplicons in (C) were subjected to 1.5% agarose gel electrophoresis.
(TIF)

S6 Fig Detection of cDNA isoforms in two domains of *PxABCC1* (A), *PxABCC2* (B) and *PxABCC3* (C) genes in susceptible and resistant *P*. *xylostella* larvae. The number and size of predicted (wild type) and alternatively spliced transcripts of each ABCC gene, as observed in PCR assays with midgut cDNA from larvae of each of the strains, are summarized. Transcript sizes are shown in base pairs (bp) and different isoforms were numbered from I to IX based on relative transcript size. Black triangles indicate approximate location of premature stop codons, inverted green triangles indicate the location of deletions (length in bp shown inside the triangles), and the inverted gray triangles indicate the location of the splice variant alleles with termination point mutation (1 bp). The blue exons indicate that these exons are relatively conserved with no deletion or alternative exon usage detected, while red exons represent the detection of deletions or alternative exon usage. The number of detected clones for each isoform among a similar number of total clones sequenced for each *P*. *xylostella* strains is summarized in the Table.
(TIF)

S7 Fig Genomic structure and phylogenetic tree of *ABCC1*, *ABCC2* and *ABCC3* genes. (A) Genomic structure of the *PxABCC1*, *PxABCC2* and *PxABCC3* genes. Putative exons are numbered and shown as vertical bars in the schematic diagram of the gDNA structure of each gene. All the exon and intron sizes are showed in scale. The featured big first intron (about 15 kb) and two alternative exons (Exon 7a and 7b) of *PxABCC1* are all shown in this figure. (B) Phylogenetic relationship between *PxABCC1*, *PxABCC2* and *PxABCC3* genes and ABCC genes from Lepidoptera. The neighbor-joining (NJ) consensus tree was generated by ClustalW alignment of the deduced amino acid sequences of ABCC genes from insect species available in the GenBank, DBM-DB, SilkDB and Manduca Base using MEGA 5.0 software [89]. Bootstrap values expressed as percentages of 1000 replications are shown at branch points. GenBank accession numbers or Gene ID are displayed within the tree and indicated in parentheses. The three *P*. *xylostella* ABCC genes discussed in this study are marked by black solid diamonds and different ABCC gene clusters are shown with different colors. Abbreviations: **Px**, *Plutella xylostella*. **Se**, *Spodoptera exigua*; **Bmo**, *Bombyx mori*; **Bma**, *Bombyx mandarina*; **Hv**, *Heliothis virescens*; **Os**, *Ostrinia scapulalis*; **Dp**, *Danaus plexippus*; **Hs**, *Heliothis subflexa*; **Ha**, *Helicoverpa armigera*; **Ms**, *Manduca sexta*.
(TIF)

S8 Fig Experimental design for analysis of linkage between resistance to Cry1Ac and differential expression of *PxmALP* and three PxABCC genes in the NIL-R strain of *P*. *xylostella*. (TIF)

S9 Fig Analysis of linkage between *PxABCC1*, *PxABCC2* and *PxABCC3* isoforms with resistance to Cry1Ac in the NIL-R strain of *P*. *xylostella*. Individual midguts from larvae in backcross family groups described in S9 Fig were used in PCR assays with primers detecting isoforms of *PxABCC1* (A), *PxABCC2* (B) and *PxABCC3* (C) as described in S4–S6 Tables and Materials and Methods. To detect the multiple-band or one-band isoform patterns for each gene, the PCR products were resolved by 1.5% agarose gel electrophoresis and then subcloned and sequenced as described in Materials and Methods.
(TIF)

S10 Fig The location of three PxABCC and *PxMAP4K4* genes in the *P*. *xylostella* genome. The GBrowse tool in the DBM-DB database was used to display the location in the *BtR-1* locus of the four genes discussed in this study (boxed in the figure).
(TIF)

S11 Fig Amino acid sequence alignment of representative MAP4K4 homologs. These genes including *NIK* (GenBank accession no. XP_005264126) from *Homo sapiens*, *mig-15* (GenBank accession no. NP_001024974) from *Caenorhabditis elegans*, *msn* (GenBank accession no. NP_995971) from *Drosophila melanogaster*, *TcasGA2* (GenBank accession no. EFA04278) from *Tribolium castaneum*, *BmMAP4K4* (SilkDB Gene ID: BGIBMGA007730) from *Bombyx mori* and *PxMAP4K4* (GenBank accession no. KM507871) from *Plutella xylostella*. Identical amino acids are shown in different colors based on their sequence identity. The N-terminal kinase domain (STKc domain) and the C-terminal regulatory domain (CNH domain) show a high degree of identity between the five proteins.
(TIF)

S12 Fig Detection of Cry1Ac toxin-induced expression of *PxMAP4K4* gene in the susceptible and resistant *P*. *xylostella* strains. Relative expression levels of *PxMAP4K4* as determined by qPCR in midguts of unselected or Cry1Ac-selected *P*. *xylostella* larvae from both susceptible DBM1Ac-S and resistant NIL-R strains. Expression of the ribosomal protein *L32* gene was used as internal reference to normalize datasets and calculate relative expression levels, which were calculated assigning a value of 1 to the expression levels in DBM1Ac-S samples. Data shown are the means and corresponding standard errors (SEM) from three biological replicates tested in four technical repeats. Different letters on the bars indicate statistically significant differences in gene expression among strains (P < 0.05; Holm-Sidak’s test; n = 3).
(TIF)

S1 Table Susceptibility to Cry1Ac toxin or a *B*. *thuringiensis* var. *kurstaki* (Btk) formulation in larvae from five strains of *Plutella xylostella*. (DOC)

S2 Table List of primers used for PxmALP study. (DOC)

S3 Table Unigenes with high identity to PxmALP, five different PxABCC, and PxMAP4K4 genes identified in an RNA-seq experiment comparing gene expression in the DBM1Ac-S (MM), DBM1Ac-R (MK), and GZ-R (GK) strains of *P*. *xylostella*. (DOC)

S4 Table Genes in the *BtR-1* resistance locus of *P*. *xylostella*. (XLS)

S5 Table List of primers used for PxABCC1 study. (DOC)

S6 Table List of primers used for PxABCC2 study. (DOC)

S7 Table List of primers used for PxABCC3 study. (DOC)

S8 Table List of primers used for PxABCC4 study. (DOC)

S9 Table List of primers used for PxABCC5 study. (DOC)

S10 Table Genomic structure of three *P*. *xylostella* ABCC genes in the *BtR-1* locus. (DOC)

S11 Table Effect of silencing PxABCC2, PxABCC3 and multigenes on biological parameters of *P*. *xylostella* strain DBM1Ac-S. (DOC)

S12 Table List of primers used for PxMAP4K4 study. (DOC)

## References

[1] Sanchis V. From microbial sprays to insect-resistant transgenic plants: history of the biopesticide *Bacillus thuringiensis*. A review. Agron Sustain Dev. 2011;31: 217–231.

[2] Crickmore N, Baum J, Bravo A, Lereclus D, Narva K, Sampson K, et al. *Bacillus thuringiensis* toxin nomenclature. 2014. http://wwwlifescisussexacuk/home/Neil_Crickmore/Bt/.

[3] James C. Global status of commercialized biotech/GM crops: 2013. ISAAA Brief No. 46. ISAAA: Ithaca, NY. 2013.

[4] Tabashnik BE, Huang F, Ghimire MN, Leonard BR, Siegfried BD, Rangasamy M, et al Efficacy of genetically modified Bt toxins against insects with different genetic mechanisms of resistance. Nat Biotechnol. 2011;29: 1128–1131. 10.1038/nbt.1988 21983521

[5] Tabashnik BE, Brévault T, Carrière Y. Insect resistance to Bt crops: lessons from the first billion acres. Nat Biotechnol. 2013;31: 510–521. 10.1038/nbt.2597 23752438

[6] Adang MJ, Crickmore N, Jurat-Fuentes JL. Diversity of *Bacillus thuringiensis* crystal toxins and mechanism of action. Adv Insect Physiol. 2014;47: 39–87.

[7] Ferré J, Van Rie J. Biochemistry and genetics of insect resistance to *Bacillus thuringiensis* . Annu Rev Entomol. 2002;47: 501–533. 11729083 10.1146/annurev.ento.47.091201.145234

[8] Pigott CR, Ellar DJ. Role of receptors in *Bacillus thuringiensis* crystal toxin activity. Microbiol Mol Biol Rev. 2007;71: 255–281. 17554045 10.1128/MMBR.00034-06 PMC1899880

[9] Gahan LJ, Pauchet Y, Vogel H, Heckel DG. An ABC transporter mutation is correlated with insect resistance to *Bacillus thuringiensis* Cry1Ac toxin. PLoS Genet. 2010;6: e1001248 10.1371/journal.pgen.1001248 21187898 PMC3002984

[10] Baxter SW, Badenes-Pérez FR, Morrison A, Vogel H, Crickmore N, Kain W, et al Parallel evolution of *Bacillus thuringiensis* toxin resistance in Lepidoptera. Genetics. 2011;189: 675–679. 10.1534/genetics.111.130971 21840855 PMC3189815

[11] Atsumi S, Miyamoto K, Yamamoto K, Narukawa J, Kawai S, Sezutsu H, et al Single amino acid mutation in an ATP-binding cassette transporter gene causes resistance to Bt toxin Cry1Ab in the silkworm, *Bombyx mori* . Proc Natl Acad Sci USA. 2012;109: E1591–E1598. 10.1073/pnas.1120698109 22635270 PMC3382473

[12] Park Y, Gonzalez-Martinez RM, Navarro-Cerrillo G, Chakroun M, Kim Y, Ziarsolo P, et al ABCC transporters mediate insect resistance to multiple Bt toxins revealed by bulk segregant analysis. BMC Biol. 2014;12: 46 10.1186/1741-7007-12-46 24912445 PMC4071345

[13] Xiao Y, Zhang T, Liu C, Heckel DG, Li X, Tabashnik BE, et al Mis-splicing of the ABCC2 gene linked with Bt toxin resistance in *Helicoverpa armigera* . Sci Rep. 2014;4: 6184 10.1038/srep06184 25154974 PMC4143771

[14] Tanaka S, Miyamoto K, Noda H, Jurat-Fuentes JL, Yoshizawa Y, Endo H, et al The ATP-binding cassette transporter subfamily C member 2 in *Bombyx mori* larvae is a functional receptor for Cry toxins from *Bacillus thuringiensis* . FEBS J. 2013;280: 1782–1794. 10.1111/febs.12200 23432933

[15] Dermauw W, Van Leeuwen T. The ABC gene family in arthropods: comparative genomics and role in insecticide transport and resistance. Insect Biochem Mol Biol. 2014;45: 89–110. 10.1016/j.ibmb.2013.11.001 24291285

[16] Merzendorfer H. ABC transporters and their role in protecting insects from pesticides and their metabolites. Adv Insect Physiol. 2014;46: 1–72.

[17] Jurat-Fuentes JL, Karumbaiah L, Jakka SR, Ning C, Liu C, Wu K, et al Reduced levels of membrane-bound alkaline phosphatase are common to lepidopteran strains resistant to Cry toxins from *Bacillus thuringiensis* . PLoS ONE. 2011;6: e17606 10.1371/journal.pone.0017606 21390253 PMC3046977

[18] Caccia S, Moar WJ, Chandrashekhar J, Oppert C, Anilkumar KJ, Jurat-Fuentes JL, et al Association of Cry1Ac toxin resistance in *Helicoverpa zea* (Boddie) with increased alkaline phosphatase levels in the midgut lumen. Appl Environ Microbiol. 2012;78: 5690–5698. 10.1128/AEM.00523-12 22685140 PMC3406154

[19] Furlong MJ, Wright DJ, Dosdall LM. Diamondback moth ecology and management: problems, progress and prospects. Annu Rev Entomol. 2013;58: 517–541. 10.1146/annurev-ento-120811-153605 23020617

[20] Tabashnik BE, Cushing NL, Finson N, Johnson MW. Field development of resistance to *Bacillus thuringiensis* in diamondback moth (Lepidoptera: Plutellidae). J Econ Entomol. 1990;83: 1671–1676.

[21] Janmaat AF, Myers J. Rapid evolution and the cost of resistance to *Bacillus thuringiensis* in greenhouse populations of cabbage loopers, *Trichoplusia ni* . Proc R Soc Lond B. 2003;270: 2263–2270. 14613613 10.1098/rspb.2003.2497 PMC1691497

[22] Tabashnik BE, Liu YB, Malvar T, Heckel DG, Masson L, Ballester V, et al Global variation in the genetic and biochemical basis of diamondback moth resistance to *Bacillus thuringiensis* . Proc Natl Acad Sci USA. 1997;94: 12780–12785. 9371752 10.1073/pnas.94.24.12780 PMC24215

[23] Wang P, Zhao JZ, Rodrigo-Simón A, Kain W, Janmaat AF, Shelton AM, et al Mechanism of resistance to *Bacillus thuringiensis* toxin Cry1Ac in a greenhouse population of the cabbage looper, *Trichoplusia ni* . Appl Environ Microbiol. 2007;73: 1199–1207. 17189446 10.1128/AEM.01834-06 PMC1828666

[24] Tiewsiri K, Wang P. Differential alteration of two aminopeptidases N associated with resistance to *Bacillus thuringiensis* toxin Cry1Ac in cabbage looper. Proc Natl Acad Sci USA. 2011;108: 14037–14042. 10.1073/pnas.1102555108 21844358 PMC3161562

[25] Baxter SW, Zhao JZ, Gahan LJ, Shelton AM, Tabashnik BE, Heckel DG. Novel genetic basis of field-evolved resistance to Bt toxins in *Plutella xylostella* . Insect Mol Biol. 2005;14: 327–334. 15926902 10.1111/j.1365-2583.2005.00563.x

[26] Baxter SW, Zhao JZ, Shelton AM, Vogel H, Heckel DG. Genetic mapping of Bt-toxin binding proteins in a Cry1A-toxin resistant strain of diamondback moth *Plutella xylostella* . Insect Biochem Mol Biol. 2008;38: 125–135. 10.1016/j.ibmb.2007.09.014 18207074

[27] Huffman DL, Abrami L, Sasik R, Corbeil J, van der Goot FG, Aroian RV. Mitogen-activated protein kinase pathways defend against bacterial pore-forming toxins. Proc Natl Acad Sci USA. 2004;101: 10995–11000. 15256590 10.1073/pnas.0404073101 PMC503732

[28] Kao CY, Los FCO, Huffman DL, Wachi S, Kloft N, Husmann M, et al Global functional analyses of cellular responses to pore-forming toxins. PLoS Pathog. 2011;7: e1001314 10.1371/journal.ppat.1001314 21408619 PMC3048360

[29] Cancino-Rodezno A, Alexander C, Villaseñor R, Pacheco S, Porta H, Pauchet Y, et al The mitogen-activated protein kinase p38 is involved in insect defense against Cry toxins from *Bacillus thuringiensis* . Insect Biochem Mol Biol. 2010;40: 58–63. 10.1016/j.ibmb.2009.12.010 20040372 PMC2827608

[30] Porta H, Cancino-Rodezno A, Soberón M, Bravo A. Role of MAPK p38 in the cellular responses to pore-forming toxins. Peptides. 2011;32: 601–606. 10.1016/j.peptides.2010.06.012 20599578 PMC2994946

[31] Krishna M, Narang H. The complexity of mitogen-activated protein kinases (MAPKs) made simple. Cell Mol Life Sci. 2008;65: 3525–3344. 10.1007/s00018-008-8170-7 18668205 PMC11131782

[32] Horton A, Wang B, Camp L, Price M, Arshi A, Nagy M, et al The mitogen-activated protein kinome from *Anopheles gambiae*: identification, phylogeny and functional characterization of the ERK, JNK and p38 MAP kinases. BMC Genomics. 2011;12: 574 10.1186/1471-2164-12-574 22111877 PMC3233564

[33] Guo Z, Kang S, Zhu X, Wu Q, Wang S, Xie W, et al The midgut cadherin-like gene is not associated with resistance to *Bacillus thuringiensis* toxin Cry1Ac in *Plutella xylostella* (L.). J Invertebr Pathol. 2015;126: 21–30. 10.1016/j.jip.2015.01.004 25595643

[34] Perera OP, Willis JD, Adang MJ, Jurat-Fuentes JL. Cloning and characterization of the Cry1Ac-binding alkaline phosphatase (HvALP) from *Heliothis virescens* . Insect Biochem Mol Biol. 2009;39: 294–302. 10.1016/j.ibmb.2009.01.006 19552892

[35] Ning C, Wu K, Liu C, Gao Y, Jurat-Fuentes JL, Gao X. Characterization of a Cry1Ac toxin-binding alkaline phosphatase in the midgut from *Helicoverpa armigera* (Hübner) larvae. J Insect Physiol. 2010;56: 666–672. 10.1016/j.jinsphys.2010.02.003 20170658

[36] Lei Y, Zhu X, Xie W, Wu Q, Wang S, Guo Z, et al Midgut transcriptome response to a Cry toxin in the diamondback moth, *Plutella xylostella* (Lepidoptera: Plutellidae). Gene. 2014;533: 180–187. 10.1016/j.gene.2013.09.091 24120626

[37] Kulkarni MM, Booker M, Silver SJ, Friedman A, Hong P, Perrimon N, et al Evidence of off-target effects associated with long dsRNAs in *Drosophila melanogaster* cell-based assays. Nat Methods. 2006;3: 833–838. 16964256 10.1038/nmeth935

[38] Gong Y, Wang C, Yang Y, Wu S, Wu Y. Characterization of resistance to *Bacillus thuringiensis* toxin Cry1Ac in *Plutella xylostella* from China. J Invertebr Pathol. 2010;104: 90–96. 10.1016/j.jip.2010.02.003 20167218

[39] Xia Q, Zhou Z, Lu C, Cheng D, Dai F, Li B, et al A draft sequence for the genome of the domesticated silkworm (*Bombyx mori*). Science. 2004;306: 1937–1940. 15591204 10.1126/science.1102210

[40] You M, Yue Z, He W, Yang X, Yang G, Xie M, et al A heterozygous moth genome provides insights into herbivory and detoxification. Nat Genet. 2013;45: 220–225. 10.1038/ng.2524 23313953

[41] Tabashnik BE, Mota-Sanchez D, Whalon ME, Hollingworth RM, Carrière Y. Defining terms for proactive management of resistance to Bt crops and pesticides. J Econ Entomol. 2014;107: 496–507. 24772527 10.1603/ec13458

[42] Tang JD, Shelton AM, Van Rie J, de Roeck S, Moar WJ, Roush RT, et al Toxicity of *Bacillus thuringiensis* spore and crystal protein to resistant diamondback moth (*Plutella xylostella*). Appl Environ Microbiol. 1996;62: 564–569. 16535241 10.1128/aem.62.2.564-569.1996 PMC1388779

[43] Zhu X, Lei Y, Yang Y, Baxter SW, Li J, Wu Q, et al Construction and characterisation of near-isogenic *Plutella xylostella* (Lepidoptera: Plutellidae) strains resistant to Cry1Ac toxin. Pest Manag Sci. 2015;71: 225–233. 10.1002/ps.3785 24687616

[44] Tabashnik BE, Liu Y-B, Malvar T, Heckel DG, Masson L, Ferré J. Insect resistance to *Bacillus thuringiensis*: uniform or diverse? Phil Trans R Soc Lond B. 1998;353: 1751–1756.

[45] Ballester V, Granero F, Tabashnik BE, Malvar T, Ferré J. Integrative model for binding of *Bacillus thuringiensis* toxins in susceptible and resistant larvae of the diamondback moth (*Plutella xylostella*). Appl Environ Microbiol. 1999;65: 1413–1419. 10103230 10.1128/aem.65.4.1413-1419.1999 PMC91200

[46] Yang ZX, Wu QJ, Wang SL, Chang XL, Wang JH, Guo ZJ, et al Expression of cadherin, aminopeptidase N and alkaline phosphatase genes in Cry1Ac-susceptible and Cry1Ac-resistant strains of *Plutella xylostella* (L.). J Appl Entomol. 2012;136: 539–548.

[47] Arenas I, Bravo A, Soberón M, Gómez I. Role of alkaline phosphatase from *Manduca sexta* in the mechanism of action of *Bacillus thuringiensis* Cry1Ab toxin. J Biol Chem. 2010;285: 12497–12503. 10.1074/jbc.M109.085266 20177063 PMC2857145

[48] Flores-Escobar B, Rodríguez-Magadan H, Bravo A, Soberón M, Gómez I. Differential role of *Manduca sexta* aminopeptidase-N and alkaline phosphatase in the mode of action of Cry1Aa, Cry1Ab, and Cry1Ac toxins from *Bacillus thuringiensis* . Appl Environ Microbiol. 2013;79: 4543–4550. 10.1128/AEM.01062-13 23686267 PMC3719532

[49] Gahan LJ, Gould F, Heckel DG. Identification of a gene associated with Bt resistance in *Heliothis virescens* . Science. 2001;293: 857–860. 11486086 10.1126/science.1060949

[50] Herrero S, Gechev T, Bakker PL, Moar WJ, de Maagd RA. *Bacillus thuringiensis* Cry1Ca-resistant *Spodoptera exigua* lacks expression of one of four Aminopeptidase N genes. BMC Genomics. 2005;6: 96 15978131 10.1186/1471-2164-6-96 PMC1184072

[51] Grailles M, Brey PT, Roth CW. The *Drosophila melanogaster* multidrug-resistance protein 1 (MRP1) homolog has a novel gene structure containing two variable internal exons. Gene. 2003;307: 41–50. 12706887 10.1016/s0378-1119(03)00455-4

[52] Roth CW, Holm I, Graille M, Dehoux P, Rzhetsky A, Wincker P, et al Identification of the *Anopheles gambiae* ATP-binding cassette transporter superfamily genes. Mol Cells. 2003;15: 150–158. 12803476

[53] Labbé R, Caveney S, Donly C. Genetic analysis of the xenobiotic resistance-associated ABC gene subfamilies of the Lepidoptera. Insect Mol Biol. 2011;20: 243–256. 10.1111/j.1365-2583.2010.01064.x 21199020

[54] Chen Y, Li M, Islam I, You L, Wang Y, Li Z, et al Allelic-specific expression in relation to *Bombyx mori* resistance to Bt toxin. Insect Biochem Mol Biol. 2014;54: 53–60. 10.1016/j.ibmb.2014.07.007 25123097

[55] Coates BS, Sumerford DV, Siegfried BD, Hellmich RL, Abel CA. Unlinked genetic loci control the reduced transcription of aminopeptidase N 1 and 3 in the European corn borer and determine tolerance to *Bacillus thuringiensis* Cry1Ab toxin. Insect Biochem Mol Biol. 2013;43: 1152–1160. 10.1016/j.ibmb.2013.09.003 24121099

[56] Suzuki A, Guicheux J, Palmer G, Miura Y, Oiso Y, Bonjour JP, et al Evidence for a role of p38 MAP kinase in expression of alkaline phosphatase during osteoblastic cell differentiation. Bone. 2002;30: 91–98. 11792570 10.1016/s8756-3282(01)00660-3

[57] Sowa H, Kaji H, Yamaguchi T, Sugimoto T, Chihara K. Activations of ERK1/2 and JNK by transforming growth factor β negatively regulate Smad3-induced alkaline phosphatase activity and mineralization in mouse osteoblastic cells. J Biol Chem. 2002;277: 36024–36031. 12130649 10.1074/jbc.M206030200

[58] Hager S, Lampert FM, Orimo H, Stark GB, Finkenzeller G. Up-regulation of alkaline phosphatase expression in human primary osteoblasts by cocultivation with primary endothelial cells is mediated by p38 mitogen-activated protein kinase-dependent mRNA stabilization. Tissue Eng Part A. 2009;15: 3437–3447. 10.1089/ten.TEA.2009.0133 19409035

[59] Sukhai M, Piquette-Miller M. Regulation of the multidrug resistance genes by stress signals. J Pharm Pharm Sci. 2000;3: 268–280. 10994039

[60] Kim SH, Bark H, Choi CH. Mercury induces multidrug resistance-associated protein gene through p38 mitogen-activated protein kinase. Toxicol Lett. 2005;155: 143–150. 15585369 10.1016/j.toxlet.2004.09.007

[61] Tan KPH, Ito S. Coordinate induction of ATP-binding cassette (ABC) proteins by bile acids via Nrf2 is mediated by mitogen activated protein kinases (MAPK). FASEB J. 2008;22: 921.919.

[62] El Azreq MA, Naci D, Aoudjit F. Collagen/β1 integrin signaling up-regulates the ABCC1/MRP-1 transporter in an ERK/MAPK-dependent manner. Mol Biol Cell. 2012;23: 3473–3484. 10.1091/mbc.E12-02-0132 22787275 PMC3431945

[63] Su YC, Treisman JE, Skolnik EY. The *Drosophila* Ste20-related kinase *misshapen* is required for embryonic dorsal closure and acts through a JNK MAPK module on an evolutionarily conserved signaling pathway. Genes Dev. 1998;12: 2371–2380. 9694801 10.1101/gad.12.15.2371 PMC317054

[64] Chapman JO, Li H, Lundquist EA. The MIG-15 NIK kinase acts cell-autonomously in neuroblast polarization and migration in *C*. *elegans* . Dev Biol. 2008;324: 245–257. 10.1016/j.ydbio.2008.09.014 18840424 PMC2642615

[65] Collins CS, Hong J, Sapinoso L, Zhou Y, Liu Z, Micklash K, et al A small interfering RNA screen for modulators of tumor cell motility identifies MAP4K4 as a promigratory kinase. Proc Natl Acad Sci USA. 2006;103: 3775–3780. 16537454 10.1073/pnas.0600040103 PMC1383649

[66] Pece S, Gutkind JS. Signaling from E-cadherins to the MAPK pathway by the recruitment and activation of epidermal growth factor receptors upon cell-cell contact formation. J Biol Chem. 2000;275: 41227–41233. 10969083 10.1074/jbc.M006578200

[67] Zhang X, Candas M, Griko NB, Taussig R, Bulla LA Jr. A mechanism of cell death involving an adenylyl cyclase/PKA signaling pathway is induced by the Cry1Ab toxin of *Bacillus thuringiensis* . Proc Natl Acad Sci USA. 2006;103: 9897–9902. 16788061 10.1073/pnas.0604017103 PMC1502550

[68] Yang Y, Zhu YC, Ottea J, Husseneder C, Leonard BR, Abel C, et al Down regulation of a gene for cadherin, but not alkaline phosphatase, associated with Cry1Ab resistance in the sugarcane borer *Diatraea saccharalis* . PLoS ONE. 2011;6: e25783 10.1371/journal.pone.0025783 21991350 PMC3185034

[69] Jin T, Chang X, Gatehouse AM, Wang Z, Edwards MG, He K. Downregulation and mutation of a cadherin gene associated with Cry1Ac resistance in the Asian corn borer, *Ostrinia furnacalis* (Guenée). Toxins. 2014;6: 2676–2693. 10.3390/toxins6092676 25216082 PMC4179154

[70] Sorrell JM, Brinon L, Baber MA, Caplan AI. Cytokines and glucocorticoids differentially regulate APN/CD13 and DPPIV/CD26 enzyme activities in cultured human dermal fibroblasts. Arch Dermatol Res. 2003;295: 160–168. 12861419 10.1007/s00403-003-0417-4

[71] Kehlen A, Geisler M, Olsen JV, Sablotzki A, Langner J, Riemann D. IL-10 and TGF-β differ in their regulation of aminopeptidase N/CD13 expression in monocytes. Int J Mol Med. 2004;13: 877–882. 15138629 10.3892/ijmm.13.6.877

[72] Yang Y, Zhu YC, Ottea J, Husseneder C, Leonard BR, Abel C, et al Molecular characterization and RNA interference of three midgut aminopeptidase N isozymes from *Bacillus thuringiensis*-susceptible and-resistant strains of sugarcane borer, *Diatraea saccharalis* . Insect Biochem Mol Biol. 2010;40: 592–603. 10.1016/j.ibmb.2010.05.006 20685334

[73] Guo Z, Kang S, Zhu X, Xia J, Wu Q, Wang S, et al Down-regulation of a novel ABC transporter gene (*Pxwhite*) is associated with Cry1Ac resistance in the diamondback moth, *Plutella xylostella* (L.). Insect Biochem Mol Biol. 2015;59: 30–40. 10.1016/j.ibmb.2015.01.009 25636859

[74] Kondrashov FA, Rogozin IB, Wolf YI, Koonin EV. Selection in the evolution of gene duplications. Genome Biol. 2002;3: research0008.0001–0008.0009. 11864370 10.1186/gb-2002-3-2-research0008 PMC65685

[75] Li X, Schuler MA, Berenbaum MR. Molecular mechanisms of metabolic resistance to synthetic and natural xenobiotics. Annu Rev Entomol. 2007;52: 231–253. 16925478 10.1146/annurev.ento.51.110104.151104

[76] Bass C, Field LM. Gene amplification and insecticide resistance. Pest Manag Sci. 2011;67: 886–890. 10.1002/ps.2189 21538802

[77] Tang JD, Gilboa S, Roush RT, Shelton AM. Inheritance, stability, and lack-of-fitness costs of field-selected resistance to *Bacillus thuringiensis* in diamondback moth (Lepidoptera: Plutellidae) from Florida. J Econ Entomol. 1997;90: 732–741.

[78] Shelton AM, Robertson JL, Tang JD, Perez C, Eigenbrode SD, Preisler HK, et al Resistance of diamondback moth (Lepidoptera: Plutellidae) to *Bacillus thuringiensis* subspecies in the field. J Econ Entomol. 1993;86: 697–705.

[79] Metz TD, Roush RT, Tang JD, Shelton AM, Earle ED. Transgenic broccoli expressing a *Bacillus thuringiensis* insecticidal crystal protein: implications for pest resistance management strategies. Mol Breeding. 1995;1: 309–317.

[80] Luo K, Banks D, Adang MJ. Toxicity, binding, and permeability analyses of four *Bacillus thuringiensis* Cry1 δ-endotoxins using brush border membrane vesicles of *Spodoptera exigua* and *Spodoptera frugiperda* . Appl Environ Microbiol. 1999;65: 457–464. 9925568 10.1128/aem.65.2.457-464.1999 PMC91047

[81] Crespo ALB, Spencer TA, Nekl E, Pusztai-Carey M, Moar WJ, Siegfried BD. Comparison and validation of methods to quantify Cry1Ab toxin from *Bacillus thuringiensis* for standardization of insect bioassays. Appl Environ Microbiol. 2008;74: 130–135. 17981939 10.1128/AEM.01855-07 PMC2223200

[82] Abbott WS. A method of computing the effectiveness of an insecticide. J Econ Entomol. 1925;18: 265–267.

[83] Finney DJ. Probit Analysis. 3rd ed London: Cambridge University Press; 1971.

[84] Wolfersberger M, Luethy P, Maurer A, Parenti P, Sacchi FV, Giordana B, et al Preparation and partial characterization of amino acid transporting brush border membrane vesicles from the larval midgut of the cabbage butterfly (*Pieris brassicae*). Comp Biochem Physiol. 1987;86A: 301–308.

[85] Bradford MM. A rapid and sensitive method for the quantitation of microgram quantities of protein utilizing the principle of protein-dye binding. Anal Biochem. 1976;72: 248–254. 942051 10.1016/0003-2697(76)90527-3

[86] Jurat-Fuentes JL, Adang MJ. Characterization of a Cry1Ac-receptor alkaline phosphatase in susceptible and resistant *Heliothis virescens* larvae. Eur J Biochem. 2004;271: 3127–3135. 15265032 10.1111/j.1432-1033.2004.04238.x

[87] Bel Y, Siqueira HAA, Siegfried BD, Ferré J, Escriche B. Variability in the cadherin gene in an *Ostrinia nubilalis* strain selected for Cry1Ab resistance. Insect Biochem Mol Biol. 2009;39: 218–223. 10.1016/j.ibmb.2008.11.005 19114103

[88] Xie W, Lei Y, Fu W, Yang Z, Zhu X, Guo Z, et al Tissue-specific transcriptome profiling of *Plutella xylostella* third instar larval midgut. Int J Biol Sci. 2012;8: 1142–1155. 10.7150/ijbs.4588 23091412 PMC3477684

[89] Tamura K, Peterson D, Peterson N, Stecher G, Nei M, Kumar S. MEGA5: molecular evolutionary genetics analysis using maximum likelihood, evolutionary distance, and maximum parsimony methods. Mol Biol Evol. 2011;28: 2731–2739. 10.1093/molbev/msr121 21546353 PMC3203626

[90] Livak KJ, Schmittgen TD. Analysis of relative gene expression data using real-time quantitative PCR and the 2^-ΔΔCT^ method. Methods. 2001;25: 402–408. 11846609 10.1006/meth.2001.1262

[91] Bautista MA, Miyata T, Miura K, Tanaka T. RNA interference-mediated knockdown of a cytochrome P450, *CYP6BG1*, from the diamondback moth, *Plutella xylostella*, reduces larval resistance to permethrin. Insect Biochem Mol Biol. 2009;39: 38–46. 10.1016/j.ibmb.2008.09.005 18957322

[92] Jurat-Fuentes JL, Gould FL, Adang MJ. Dual resistance to *Bacillus thuringiensis* Cry1Ac and Cry2Aa toxins in *Heliothis virescens* suggests multiple mechanisms of resistance. Appl Environ Microbiol. 2003;69: 5898–5906. 14532042 10.1128/AEM.69.10.5898-5906.2003 PMC201244

[93] Dechklar M, Tiewsiri K, Angsuthanasombat C, Pootanakit K. Functional expression in insect cells of glycosylphosphatidylinositol-linked alkaline phosphatase from *Aedes aegypti* larval midgut: a *Bacillus thuringiensis* Cry4Ba toxin receptor. Insect Biochem Mol Biol. 2011;41: 159–166. 10.1016/j.ibmb.2010.11.006 21146607

[94] Jiménez AI, Reyes EZ, Cancino-Rodezno A, Bedoya-Pérez LP, Caballero-Flores GG, Muriel-Millan LF, et al *Aedes aegypti* alkaline phosphatase ALP1 is a functional receptor of *Bacillus thuringiensis* Cry4Ba and Cry11Aa toxins. Insect Biochem Mol Biol. 2012;42: 683–689. 10.1016/j.ibmb.2012.06.001 22728570 PMC3416946

