# Supplementary figures and images for: MAPK Signaling Pathway Alters Expression of Midgut ALP and ABCC Genes and Causes Resistance to Bacillus thuringiensis Cry1Ac Toxin in Diamondback Moth
Source: PLoS Genet. 2015 Apr 13;11(4):e1005124. doi: 10.1371/journal.pgen.1005124 (PMC4395465; doi:10.1371/journal.pgen.1005124)

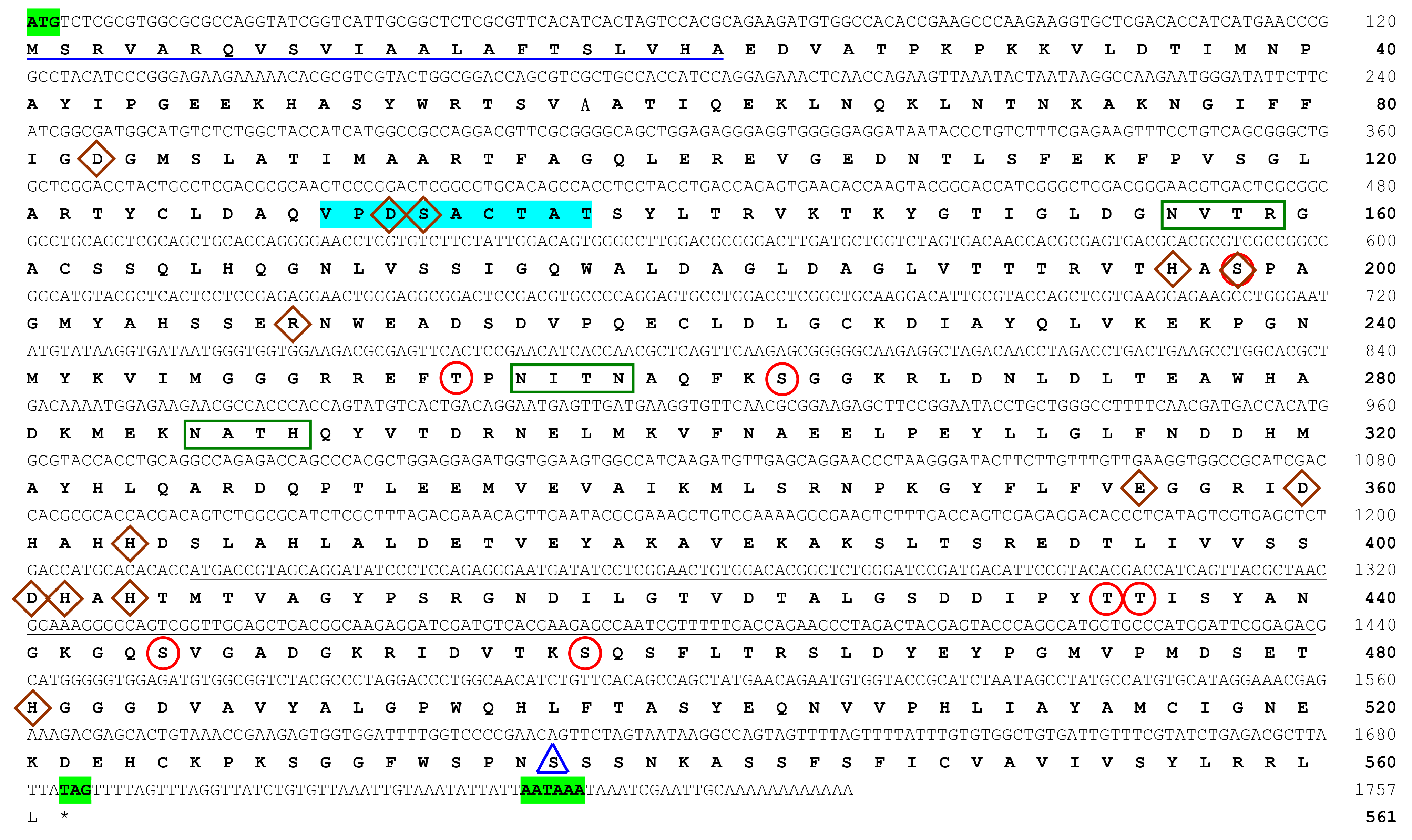

Supplement: S1 Fig — Numbers on the right indicate the nucleotide (upper) and amino acid (lower) position of PxmALP gene (GenBank accession no. KC841472). The start codon (ATG), stop codon (TAG) and putative polyadenylation signal (AATAAA) of the cDNA sequence are highlighted in green. The signal peptide is underlined in blue. The thirteen specific amino acid residues involved in substrate or metal ligand binding are enclosed by brown diamonds. The GPI- anchoring site is marked by a blue triangle. The three predicted N-glycosylation sites are inside green square boxes, and the seven putative O-glycosylation sites are red-circled. The active phosphatase site and functional residues are marked with a black character or shaded in light blue, respectively. The fragment conserved in the ALP1 gene (GenBank accession no. EF579960) exon is shown underlined in pink. (TIF) [file pgen.1005124.s001.tif]

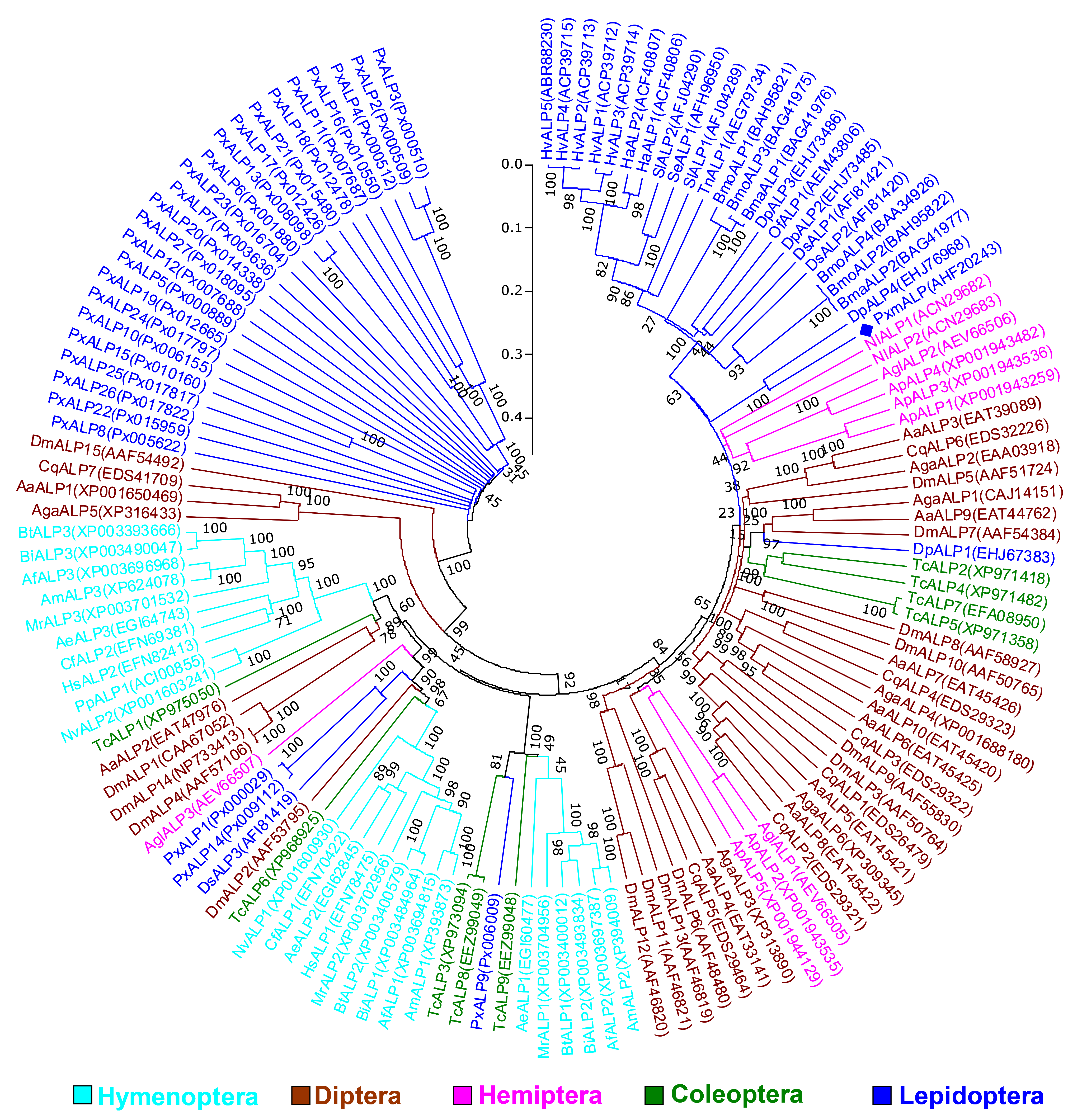

Supplement: S2 Fig — A neighbor-joining (NJ) consensus tree was generated by ClustalW alignment of the ALP amino acid sequences from different insect species available in the GenBank or Diamondback moth Genome (http://iae.fafu.edu.cn/DBM/index.php) databases using MEGA 5.0 software [89]. The bootstrap values expressed as percentages of 1000 replications are shown at branch points, values lower than 10% were hidden in the tree. GenBank accession numbers or Gene ID are displayed within the tree and indicated in parentheses. The PxmALP protein is marked by a blue solid diamond. The ALP genes from different orders are shown in different colors. Abbreviations: 1. Lepidoptera (Bmo, Bombyx mori; Bma, Bombyx mandarina; Ha, Helicoverpa armigera; Hv, Heliothis virescens; Of, Ostrinia furnacalis; Tn, Trichoplusia ni; Se, Spodoptera exigua; Dp, Danaus plexippus; Ds, Diatraea saccharalis; Sl, Spodoptera litura; Px, Plutella xylostella); 2. Diptera (Dm, Drosophila melanogaster; Aa, Aedes aegypti; Aga, Anopheles gambiae; Cq, Culex quinquefasciatus); 3. Coleoptera (Tc, Tribolium castaneum); 4. Hemiptera (Agl, Aphis glycines; Nl, Nilaparvata lugens; Ap, Acyrthosiphon pisum); 5. Hymenoptera (Pp, Pteromalus puparum; Nv, Nasonia vitripennis; Am, Apis mellifera; Ae, Acromyrmex echinatior; Hs, Harpegnathos saltator; Cf, Camponotus floridanus; Mr, Megachile rotundata; Af, Apis florae; Bi, Bombus impatiens; Bt, Bombus terrestris). (TIF) [file pgen.1005124.s002.tif]

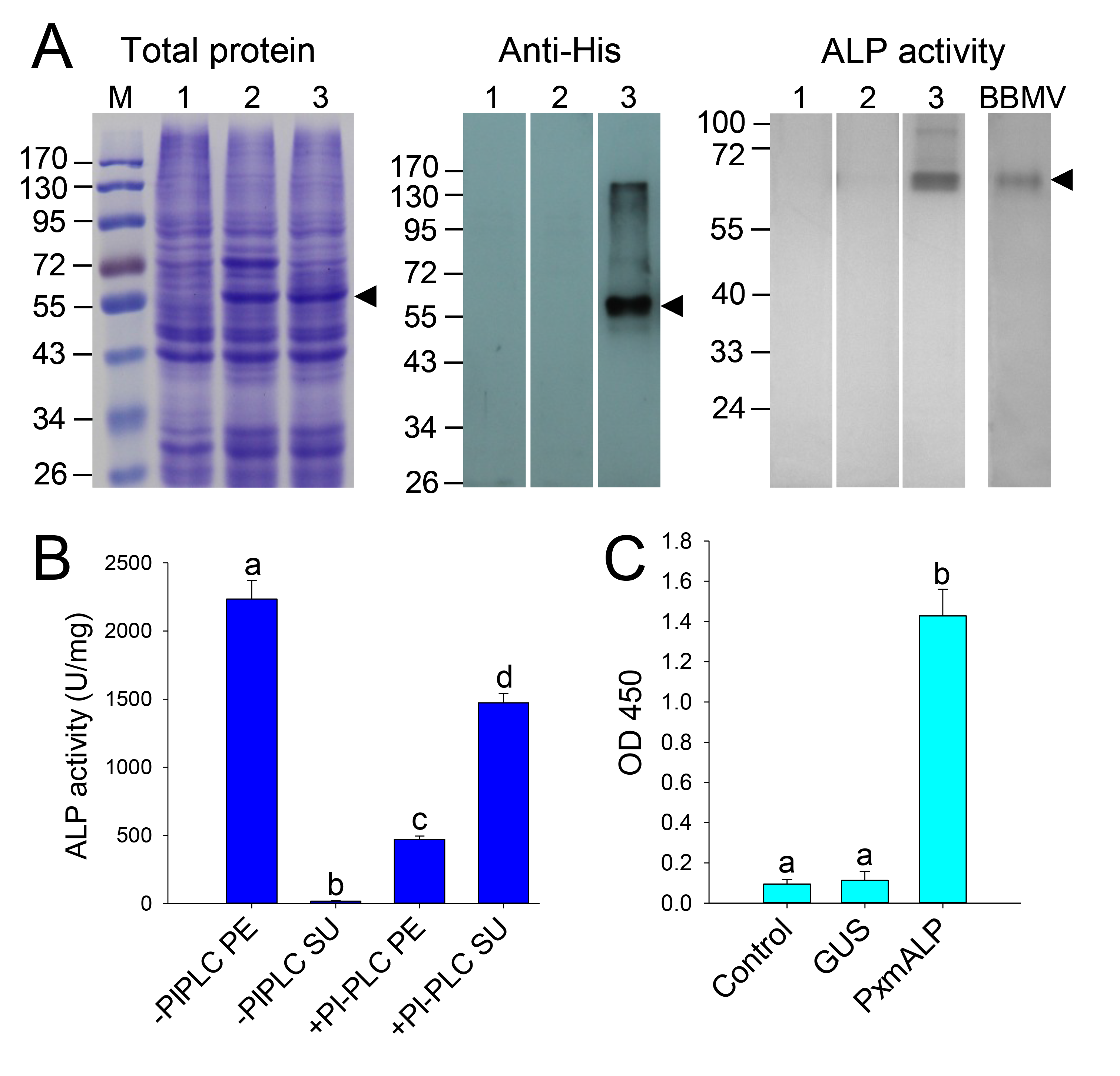

Supplement: S3 Fig — (A) Detection of PxmALP expression in Sf9 cells by Western blotting with antisera to a 6×His tag (Anti-His) at the N-terminal end of the recombinant PxmALP protein and by colorimetric detection of ALP activity (ALP activity) as described elsewhere [86]. In all panels, lanes 1 are cells transfected with empty vector; lanes 2, cells transfected to produce the GUS protein; lanes 3, cells transfected to express PxmALP. Left panel (Total protein) is a Coomassie blue-stained gel to demonstrate equal lane loading. A lane from a gel containing BBMV proteins (20 μg) of P. xylostella (Px BBMV) is shown as reference of PxmALP in larval midgut. The loading order of the lanes in the ALP activity gel has been altered to facilitate comparison between panels. (B) Confirmation that recombinant PxmALP is GPI-anchored to the Sf9 cell surface. Cultures of Sf9 cells expressing PxmALP were treated with buffer (-PI-PLC) or PI-PLC (+PI-PLC) as described in Materials and Methods, and then solubilized proteins recovered in supernatants after centrifugation. Specific ALP activities in cell pellets (PE) and solubilized proteins present in supernatants (SU) are shown. Data shown are the means and standard errors (SEM) from triplicate determinations using three independent biological samples (P < 0.05, Holm-Sidak’s test; n = 3). Different letters within a treatment denote significant differences between samples. (C) ELISA assay testing binding of Cry1Ac toxin to recombinant PxmALP expressed in Sf9 cell cultures. Binding of recombinant protein from non-transfected cells (Control), cells expressing the GUS gene (GUS), or cells expressing PxmALP (PxmALP) to Cry1Ac on the ELISA plate was detected using anti-His antibody. Bars denote the mean and standard error (SEM) values from three biological replicates, each tested at least in triplicate. Different letters denote significant differences (P < 0.05, Holm-Sidak’s test; n = 3). (TIF) [file pgen.1005124.s003.tif]

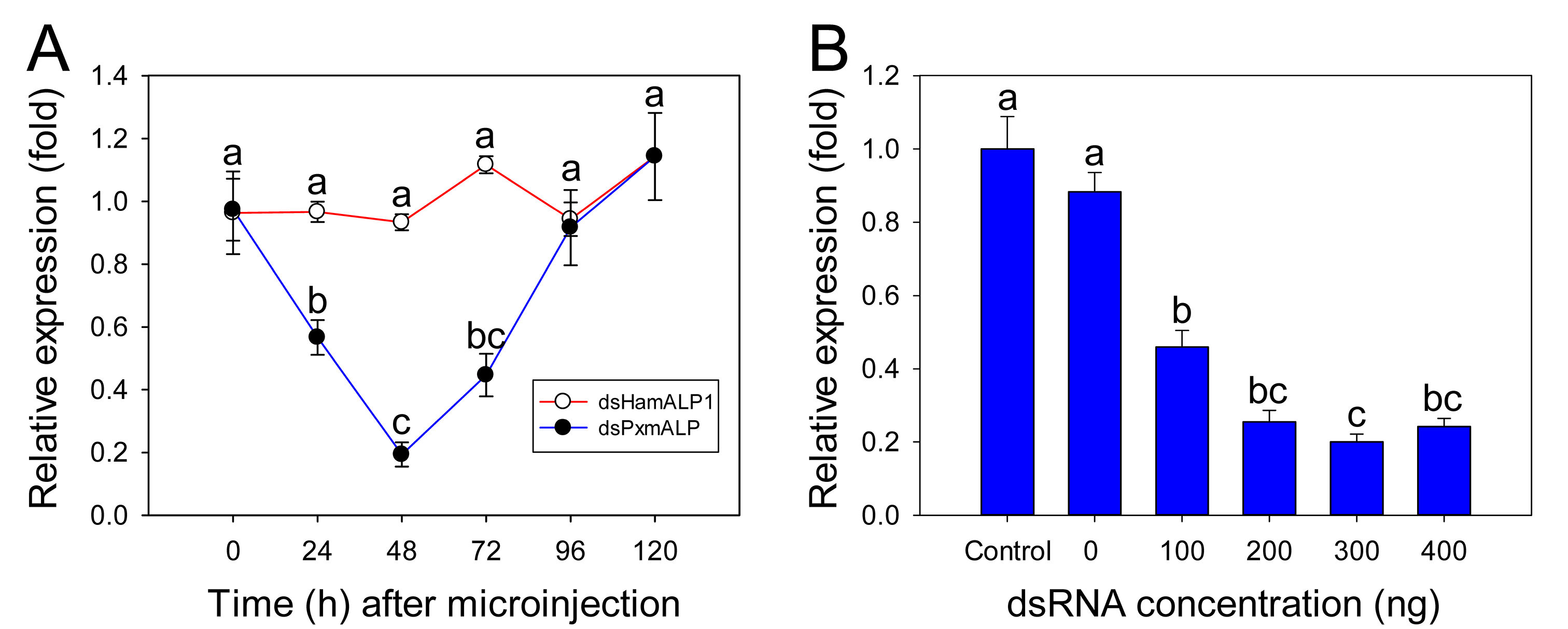

Supplement: S4 Fig — (A) Silencing of the target PxmALP gene by injection of P. xylostella larvae with 300 ng of dsRNA (dsPxmALP) was detected at different times post-injection by qPCR. As a control, larvae were injected with buffer or dsRNA targeting the mALP1 gene from H. armigera (dsHamALP1). Quantification of PxmALP expression levels in reference to the ribosomal protein L32 gene for larvae injected with dsHamALP1 or dsPxmALP is shown, and the template for each reaction was cDNA prepared from pools of 10 larvae. Each point represents the mean and standard error (SEM) from three biological replicates performed in quadruplicate. Different letters represent significant differences in expression levels between treatments (P < 0.05; Holm-Sidak’s test; n = 3). (B) Quantification by qPCR of PxmALP expression levels at 48 h post-injection when larvae were injected with increasing dsRNA concentrations (70 nl final volume). For each qPCR reaction pools of cDNA from 10 larvae were used. Expression levels for ribosomal protein L32 gene were used as reference gene and to confirm the integrity of the cDNA. Expression levels for PxmALP in non-injected larvae (control) were used as the maximum relative expression levels for comparisons among treatments. Bars represent the means and standard errors (SEM) from three biological replicates performed in quadruplicate, with different letters indicating significant differences (P < 0.05; Holm-Sidak’s test; n = 3). (TIF) [file pgen.1005124.s004.tif]

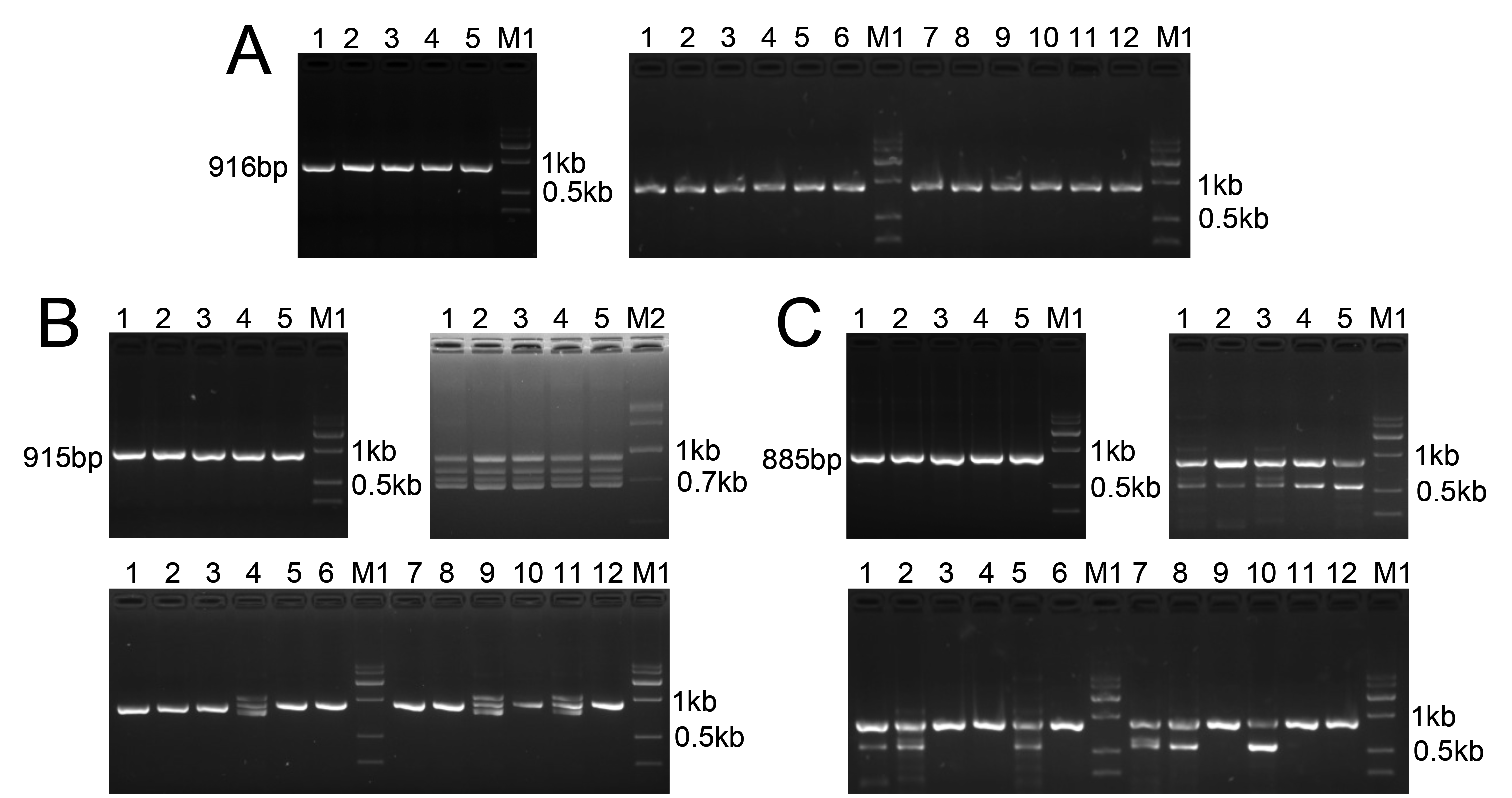

Supplement: S5 Fig — In (A), (B) and (C), lane 1: DBM1Ac-S; lane 2: DBM1Ac-R; lane 3: NIL-R; lane 4: SZ-R; lane 5: SH-R; lane M: molecular size markers. (A) Detection of an expected 916 bp amplicon based on the GenBank sequence of PxABCC1 (accession no. KM245560) in midgut samples from susceptible and resistant strains (left figure) and single midgut cDNA samples from untreated 4th instar larvae of DBM1Ac-S (lanes 1–6) and larvae of the NIL-R strain surviving exposure to 10000 μg/ml of Cry1Ac protoxin (right figure, lanes 7–12). Although the amplicons from the alternatively spliced isoform in this case appears with the same size as the wild type, sequencing results confirmed that amplicons in lanes 2, 4, 5, 7, 9, 10, 11 represent alternative splicing isoforms. (B) Detection of an expected 915 bp amplicon (top left figure) or three additional amplicons corresponding to PxABCC2 mutant isoforms ranging from 600–1000 bp in size (top right figure) based on the GenBank sequence (accession no. KM245562) in midgut samples from susceptible and resistant strains. Also shown is detection of PxABCC2 isoforms by PCR using single midgut cDNA samples from untreated 4th instar larvae of DBM1Ac-S (lanes 1–6) and larvae of the NIL-R strain surviving exposure to 10000 μg/ml of Cry1Ac protoxin (bottom figure, lanes 7–12). (C) Detection of an expected 885 bp amplicon (top left figure) or additional amplicons corresponding to PxABCC3 mutant isoforms ranging from 500–1000 bp in size (top right figure) based on the GenBank sequence (accession no. KM245562) in midgut samples from susceptible and resistant strains. Also shown is detection of PxABCC3 isoforms by PCR using single midgut cDNA samples from untreated 4th instar larvae of DBM1Ac-S (lanes 1–6) and larvae of the NIL-R strain surviving exposure to 10000 μg/ml of Cry1Ac protoxin (bottom figure, lanes 7–12). Amplicons in (A) and (B) were separated by 2.5% agarose gel electrophoresis, while amplicons in (C) were subjected to 1.5% agarose gel electrophoresis [file pgen.1005124.s005.tif]

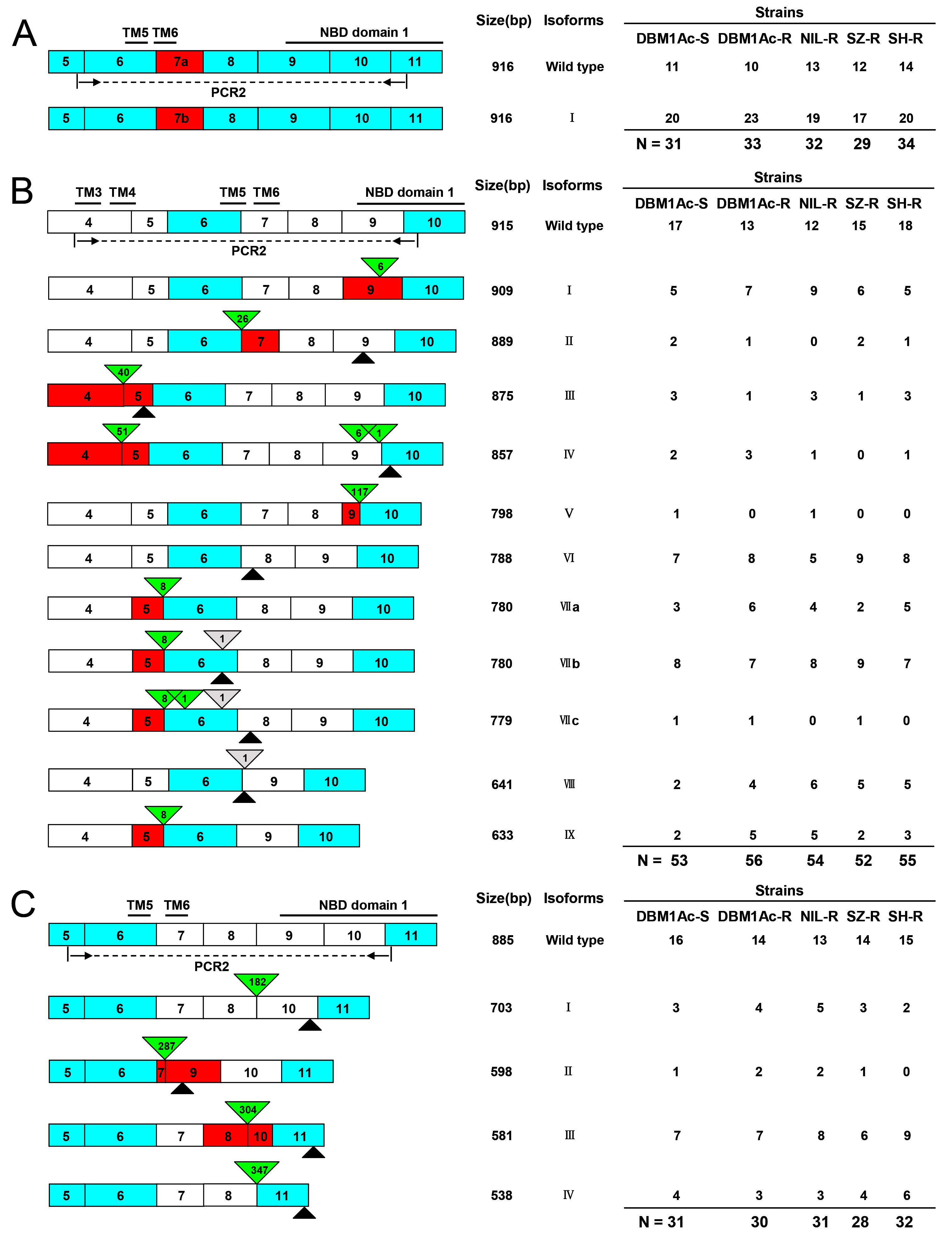

Supplement: S6 Fig — The number and size of predicted (wild type) and alternatively spliced transcripts of each ABCC gene, as observed in PCR assays with midgut cDNA from larvae of each of the strains, are summarized. Transcript sizes are shown in base pairs (bp) and different isoforms were numbered from I to IX based on relative transcript size. Black triangles indicate approximate location of premature stop codons, inverted green triangles indicate the location of deletions (length in bp shown inside the triangles), and the inverted gray triangles indicate the location of the splice variant alleles with termination point mutation (1 bp). The blue exons indicate that these exons are relatively conserved with no deletion or alternative exon usage detected, while red exons represent the detection of deletions or alternative exon usage. The number of detected clones for each isoform among a similar number of total clones sequenced for each P. xylostella strains is summarized in the Table. (TIF) [file pgen.1005124.s006.tif]

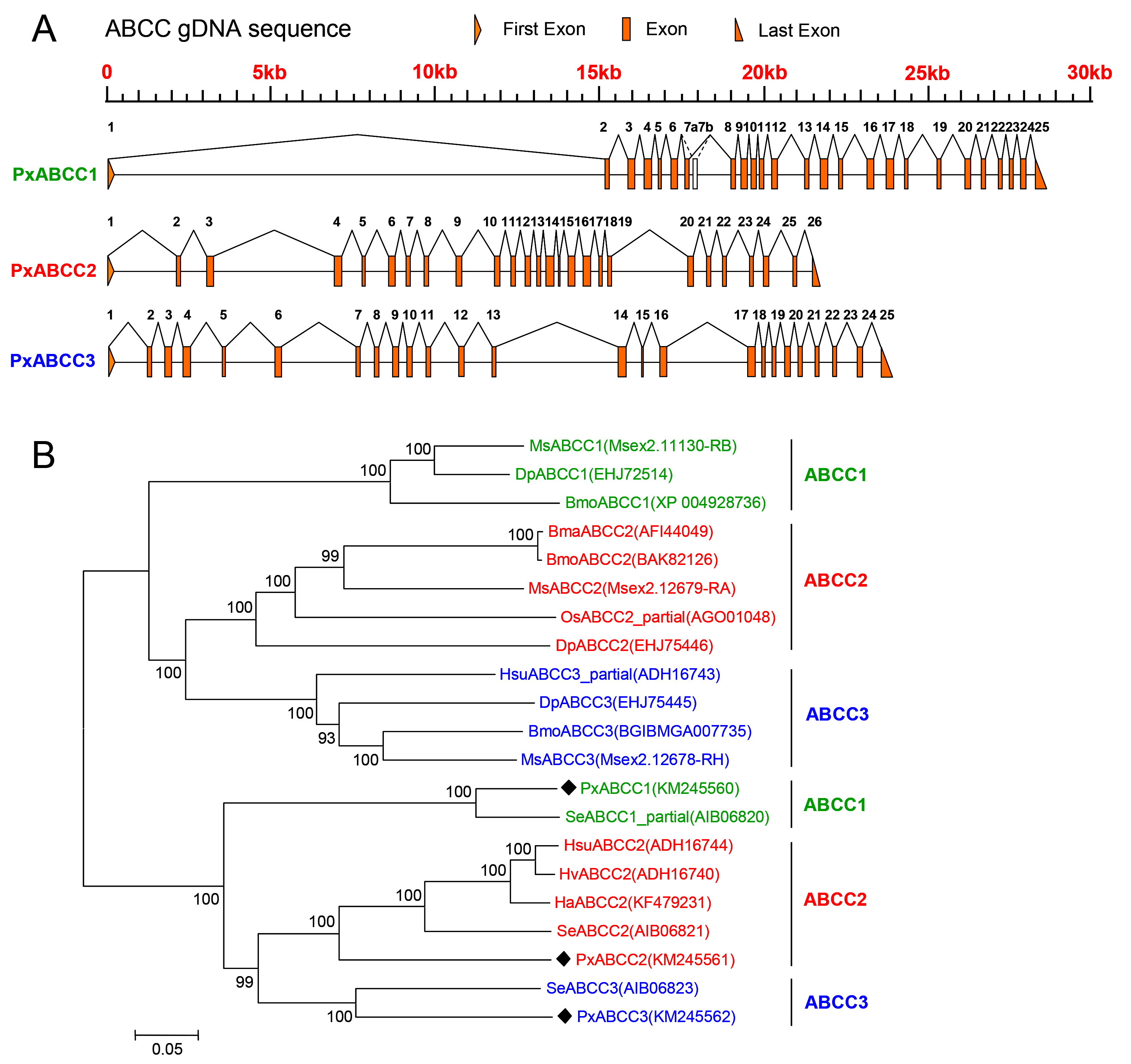

Supplement: S7 Fig — (A) Genomic structure of the PxABCC1, PxABCC2 and PxABCC3 genes. Putative exons are numbered and shown as vertical bars in the schematic diagram of the gDNA structure of each gene. All the exon and intron sizes are showed in scale. The featured big first intron (about 15 kb) and two alternative exons (Exon 7a and 7b) of PxABCC1 are all shown in this figure. (B) Phylogenetic relationship between PxABCC1, PxABCC2 and PxABCC3 genes and ABCC genes from Lepidoptera. The neighbor-joining (NJ) consensus tree was generated by ClustalW alignment of the deduced amino acid sequences of ABCC genes from insect species available in the GenBank, DBM-DB, SilkDB and Manduca Base using MEGA 5.0 software [89]. Bootstrap values expressed as percentages of 1000 replications are shown at branch points. GenBank accession numbers or Gene ID are displayed within the tree and indicated in parentheses. The three P. xylostella ABCC genes discussed in this study are marked by black solid diamonds and different ABCC gene clusters are shown with different colors. Abbreviations: Px, Plutella xylostella. Se, Spodoptera exigua; Bmo, Bombyx mori; Bma, Bombyx mandarina; Hv, Heliothis virescens; Os, Ostrinia scapulalis; Dp, Danaus plexippus; Hs, Heliothis subflexa; Ha, Helicoverpa armigera; Ms, Manduca sexta. (TIF) [file pgen.1005124.s007.tif]

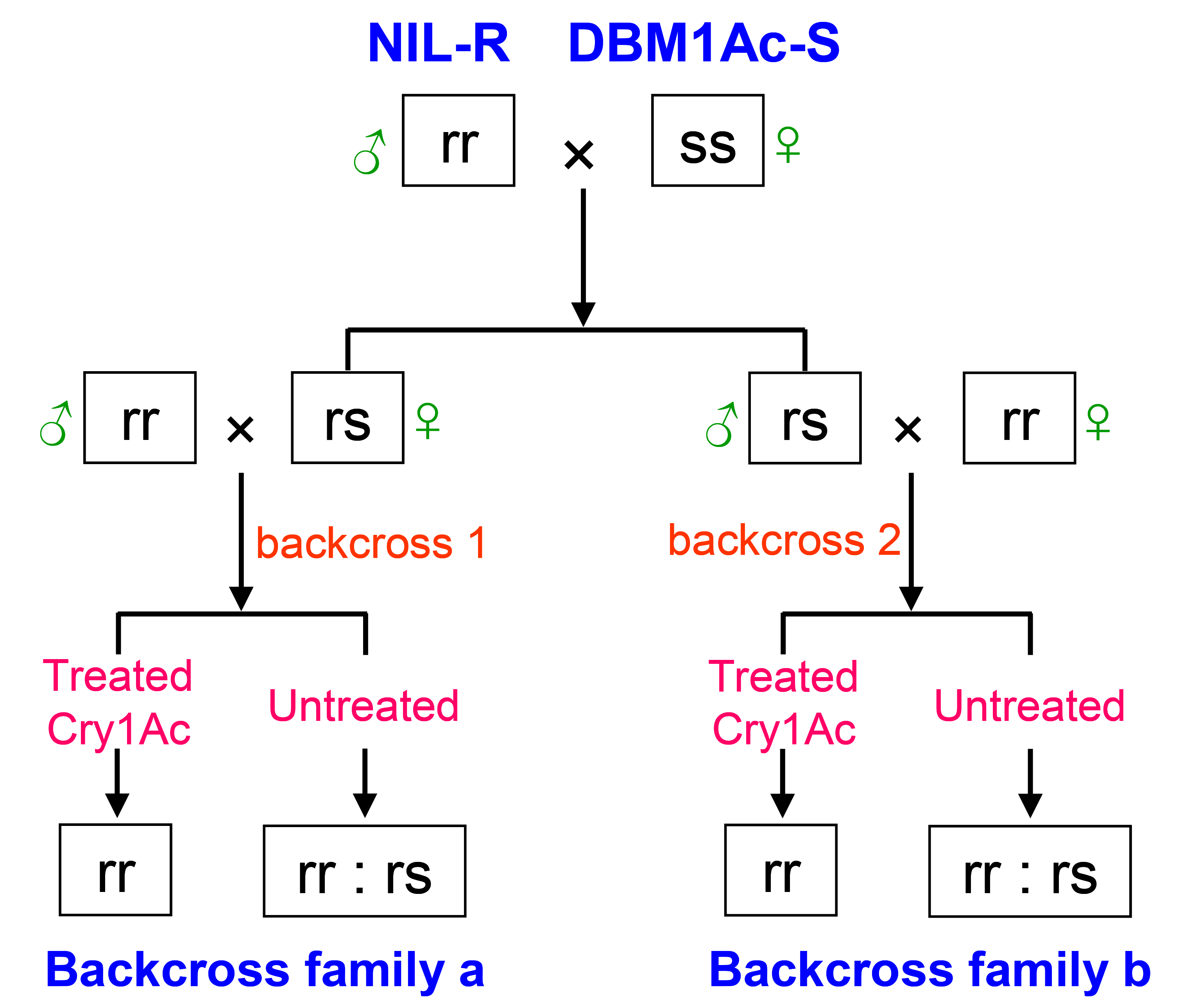

Supplement: S8 Fig — (TIF) [file pgen.1005124.s008.tif]

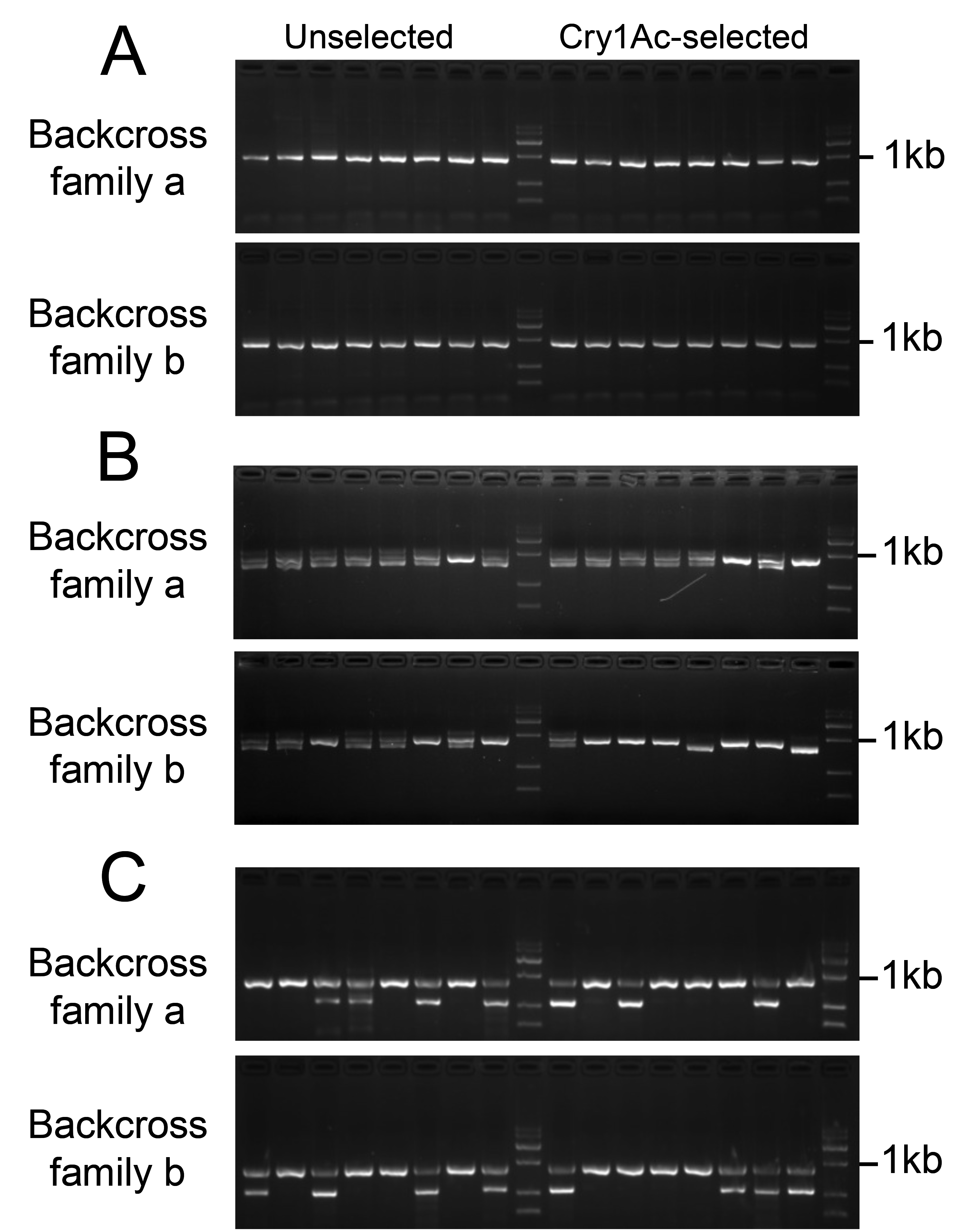

Supplement: S9 Fig — Individual midguts from larvae in backcross family groups described in S9 Fig were used in PCR assays with primers detecting isoforms of PxABCC1 (A), PxABCC2 (B) and PxABCC3 (C) as described in S4–S6 Tables and Materials and Methods. To detect the multiple-band or one-band isoform patterns for each gene, the PCR products were resolved by 1.5% agarose gel electrophoresis and then subcloned and sequenced as described in Materials and Methods. (TIF) [file pgen.1005124.s009.tif]

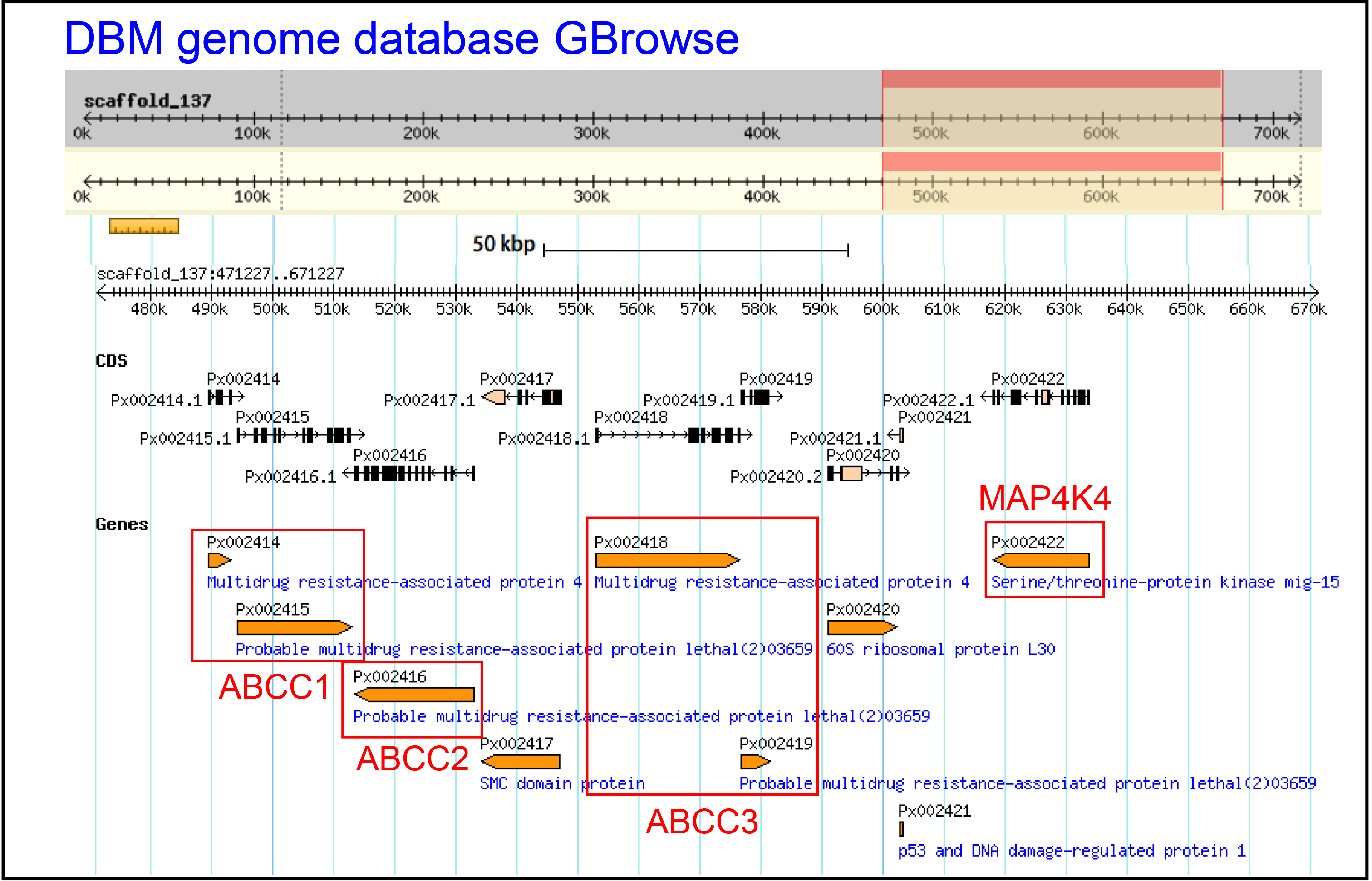

Supplement: S10 Fig — The GBrowse tool in the DBM-DB database was used to display the location in the BtR-1 locus of the four genes discussed in this study (boxed in the figure). (TIF) [file pgen.1005124.s010.tif]

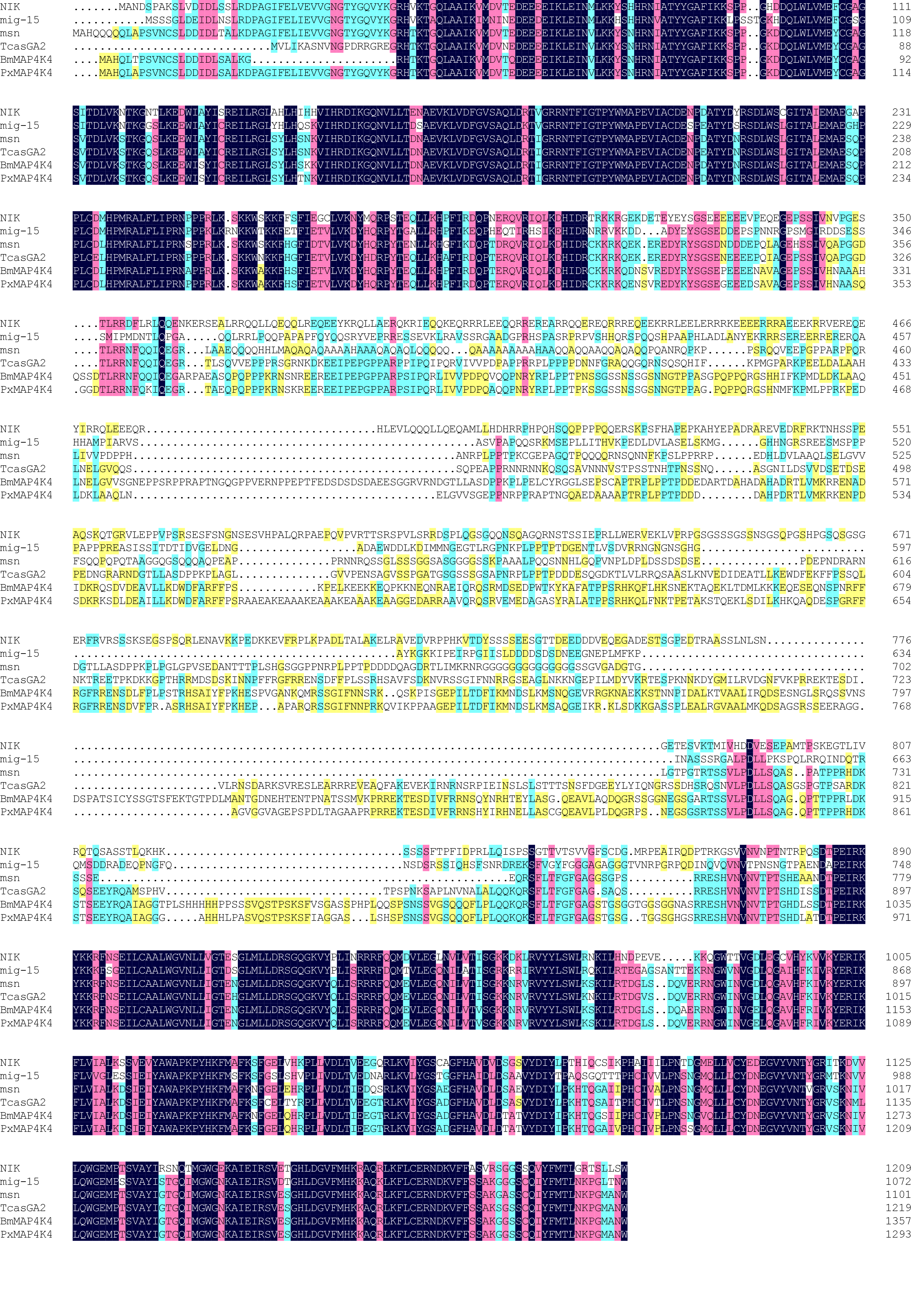

Supplement: S11 Fig — These genes including NIK (GenBank accession no. XP_005264126) from Homo sapiens, mig-15 (GenBank accession no. NP_001024974) from Caenorhabditis elegans, msn (GenBank accession no. NP_995971) from Drosophila melanogaster, TcasGA2 (GenBank accession no. EFA04278) from Tribolium castaneum, BmMAP4K4 (SilkDB Gene ID: BGIBMGA007730) from Bombyx mori and PxMAP4K4 (GenBank accession no. KM507871) from Plutella xylostella. Identical amino acids are shown in different colors based on their sequence identity. The N-terminal kinase domain (STKc domain) and the C-terminal regulatory domain (CNH domain) show a high degree of identity between the five proteins. (TIF) [file pgen.1005124.s011.tif]

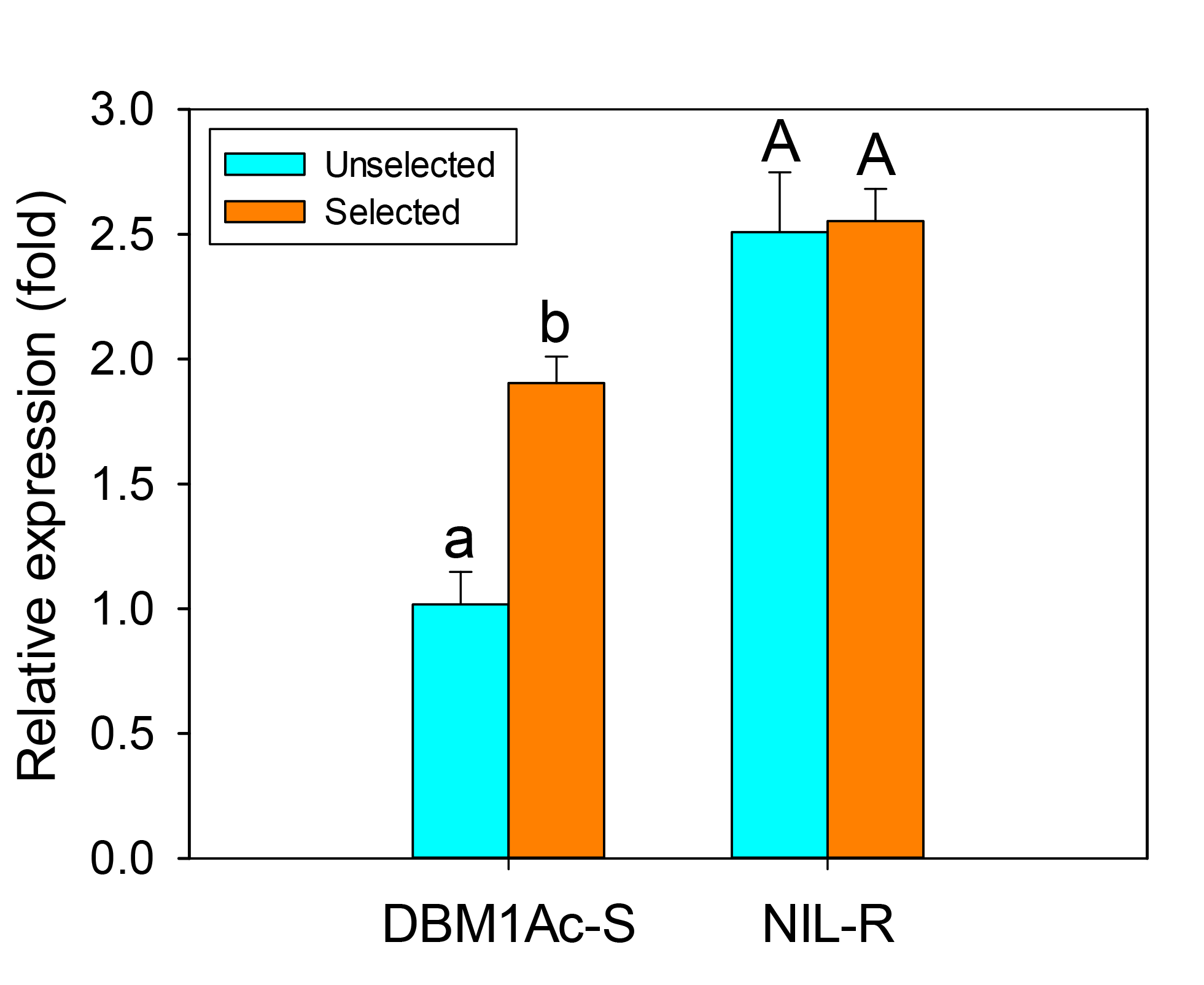

Supplement: S12 Fig — Relative expression levels of PxMAP4K4 as determined by qPCR in midguts of unselected or Cry1Ac-selected P. xylostella larvae from both susceptible DBM1Ac-S and resistant NIL-R strains. Expression of the ribosomal protein L32 gene was used as internal reference to normalize datasets and calculate relative expression levels, which were calculated assigning a value of 1 to the expression levels in DBM1Ac-S samples. Data shown are the means and corresponding standard errors (SEM) from three biological replicates tested in four technical repeats. Different letters on the bars indicate statistically significant differences in gene expression among strains (P < 0.05; Holm-Sidak’s test; n = 3). (TIF) [file pgen.1005124.s012.tif]
