# Supplementary material for: MAPK Signaling Pathway Alters Expression of Midgut ALP and ABCC Genes and Causes Resistance to Bacillus thuringiensis Cry1Ac Toxin in Diamondback Moth
Source: PLoS Genet. 2015 Apr 13;11(4):e1005124. doi: 10.1371/journal.pgen.1005124 (PMC4395465; doi:10.1371/journal.pgen.1005124)
Supplement: S1 Table — (DOC) [file pgen.1005124.s013.doc]

**S1 Table. Susceptibility to Cry1Ac toxin or a *B. thuringiensis* var. *kurstaki* (Btk) formulation in larvae from five strains of *Plutella xylostella*.**

| Strains | Treatment | Slope (±SEM) | LC_50_^a^(95% FL^b^) | RR^c^ |
| --- | --- | --- | --- | --- |
|  |  |  |  |  |
| DBM1Ac-S | Cry1Ac | 1.72 (±0.28) | 0.86 (0.51-1.45) | 1 |
|  | Btk | 2.28 (±0.28) | 0.70 (0.50-0.96) | 1 |
| DBM1Ac-R | Cry1Ac | 1.30 (±0.22) | 3,052.33 (1,732.51-5,377.59) | 3,550 |
| NIL-R | Cry1Ac | 2.56 (±0.31) | 3,401.51 (2,266.76-5,329.60) | 3,928 |
|  | Btk | 2.34 (±0.34) | 2,009.26 (1377.60-2,930.57) | 2,870 |
| SZ-R | Cry1Ac | 2.36 (±0.38) | 563.06 (363.82-871.42) | 458 |
| SH-R | Btk | 1.77 (±0.30) | 1,323.18 (780.17-2,244.14) | 1,890 |

^a^In μg/ml of diet.

^b^FL: Fiducial limits (lower-upper) of the calculated LC_50_.

^c^RR: Relative resistance ratio calculated as LC_50_ of resistant divided by LC_50_ of DBM1Ac-S.
