# Supplementary material for: MAPK Signaling Pathway Alters Expression of Midgut ALP and ABCC Genes and Causes Resistance to Bacillus thuringiensis Cry1Ac Toxin in Diamondback Moth
Source: PLoS Genet. 2015 Apr 13;11(4):e1005124. doi: 10.1371/journal.pgen.1005124 (PMC4395465; doi:10.1371/journal.pgen.1005124)
Supplement: S2 Table — (DOC) [file pgen.1005124.s014.doc]

**S2 Table. List of primers used for PxmALP study.**

| Primers used to clone the full length PxmALP cDNA | | | | |  |
| --- | --- | --- | --- | --- | --- |
| Primer name | | Primer type^a^ | Primer sequence (5′-3′) | PCR product size (bp) |  |
| De-ALP-F | | D | GAMTACMTGHTSGGACTGTTCG | 536 |  |
| De-ALP-R | | D | ACGTCDTCKCCKCCGCCGTGCGTCTC |  |  |
| 3′-ALP Outer S | | N | CCTAAGGGATACTTCTTGTTTGTTG | 452 |  |
| 3′-ALP Inner S | | N | ATCAGTTACGCAAACGGGAAGG |  |  |
| 3′-RACE Outer Primer | | A | TACCGTCGTTCCACTAGTGATTT |  |  |
| 3′-RACE Inner Primer | | A | CGCGGATCCTCCACTAGTGATTTCACTATAGG |  |  |
| 5′-ALP Outer S | | N | GTATTCAACTGTCTCGTCTAAAGCG | 1048 |  |
| 5′-ALP Inner S | | N | GCCACTTCCACCATCTCCTCC |  |  |
| Universal Primer A Mix Short | | A | CTAATACGACTCACTATAGGGC |  |  |
| Nested Universal Primer A | | A | AAGCAGTGGTATCAACGCAGAGT |  |  |
| ALP-Full-F | | F | GAGTCAGTCATGTCTCGCGT | 1695 |  |
| ALP-Full-R | | F | CTATAATAAGCGTCTCAGATACG |  |  |
| Primers used for qPCR analysis, dsRNA synthesis and heterologous expression of PxmALP as indicated | | | | | |
| Purpose | Gene name | Primer name | Primer sequence (5′-3′) | PCR product size (bp) | Positions (bp)^d^ |
| qPCR analysis | PxmALP | qALP-F1 | GCACACACCATGACCGTAGCAG | 169 | 1207–1375 |
|  |  | qALP-R1 | GGCTCTTCGTGACATCG |  |  |
|  | L32 | qL32-F | CCAATTTACCGCCCTACC | 120 | — |
|  |  | qL32-R | TACCCTGTTGTCAATACCTCT |  |  |
| Heterologous expression | PxmALP | ALP-Exp-F^c^ | CG*GAATTC*AA**ATG**TCTCGCGTGGCGCGCCA | 1686 | 1–1686 |
|  |  | ALP-Exp-R^c^ | GC*TCTAGA***CTA**TAATAAGCGTCTCAGATACG |  |  |
| dsRNA synthesis^b^ | PxmALP | dsPxmALP-F | T7-CCTCGTGTCTTCTATTGG | 438 | 510–883 |
|  |  | dsPxmALP-R | T7-CCTTCATCAACTCATTCCT |  |  |
|  | HamALP1 | dsHamALP1-F | T7-TCGTATCACTCACGCGTCTC | 538 | — |
|  |  | dsHamALP1-R | T7-TCCACGAACAGGAAGAATCC |  |  |

^a^D: degenerate primer; N: nested primer; A: adapter primer; F: coding sequence specific primer.

^b^Forward and reverse primers to synthesize dsRNA template have the T7 RNA polymerase promoter sequence (5′-TAATACGACTCACTATAGGGAGA-3′) appended to their 5′ and 3′ ends, respectively.

^c^ALP-Exp-F included an *Eco*RΙ site (underlined and italicized) and an initiation codon (bold), ALP-Exp-R contained an *Xba*Ι site (underlined and italicized) and a stop codon (bold).

^d^Positions corresponding to the full-length cDNA coding sequence of *PxmALP* deposited in GenBank database (accession no. KC841472).
