# Supplementary material for: MAPK Signaling Pathway Alters Expression of Midgut ALP and ABCC Genes and Causes Resistance to Bacillus thuringiensis Cry1Ac Toxin in Diamondback Moth
Source: PLoS Genet. 2015 Apr 13;11(4):e1005124. doi: 10.1371/journal.pgen.1005124 (PMC4395465; doi:10.1371/journal.pgen.1005124)
Supplement: S3 Table — (DOC) [file pgen.1005124.s015.doc]

**S3 Table. Unigenes with high identity to PxmALP, five different PxABCC, and PxMAP4K4 genes identified in an RNA-seq experiment comparing gene expression in the DBM1Ac-S (MM), DBM1Ac-R (MK), and GZ-R (GK) strains of *P. xylostella*.**

| **Gene name** | **Sequence ID** | **Length (bp)** | **[FDR](http://blast.ncbi.nlm.nih.gov/Blast.cgi?CMD=Get&ALIGNMENTS=100&ALIGNMENT_VIEW=Pairwise&DATABASE_SORT=0&DESCRIPTIONS=100&FIRST_QUERY_NUM=0&FORMAT_OBJECT=Alignment&FORMAT_PAGE_TARGET=&FORMAT_TYPE=HTML&GET_SEQUENCE=yes&I_THRESH=&MASK_CHAR=2&MASK_COLOR=1&NEW_VIEW=yes&NUM_OVERVIEW=100&OLD_BLAST=false&PAGE=Translations&QUERY_INDEX=0&QUERY_NUMBER=0&RESULTS_PAGE_TARGET=&RID=K3U7CYHZ01S&SHOW_LINKOUT=yes&SHOW_OVERVIEW=yes&STEP_NUMBER=&WORD_SIZE=3&DISPLAY_SORT=0&HSP_SORT=0#sort_mark)-value^a^** | | **FC^b^** | | **Expression** |
| --- | --- | --- | --- | --- | --- | --- | --- |
|  |  |  | **MK** | **GK** | **Mk/mm** | **gk/mm** |  |
| **PxmALP** | Unigene51596_mk | 153 | 0.670 | 0.843 | **-1.53** | **-0.45** | **Down regulation** |
| **PxABCC1** | Unigene38785_mk | 1329 | 6.00e-04 | 6.81e-07 | **1.18** | **1.51** | **Up regulation** |
|  | Unigene50671_mk | 561 | 0.12328 | 0.64662 | **1.47** | **-0.96** |  |
|  | Unigene38174_mk | 495 | 7.62e-03 | 3.95e-04 | **1.27** | **1.52** |  |
|  | Unigene50672_mk | 561 | 0.017486 | 0.017487 | **1.43** | **2.31** |  |
|  | Unigene50740_mk | 363 | 0.089471 | 0.36288 | **1.06** | **0.72** |  |
|  | Unigene51522_mk | 415 | 0.017240 | 0.027931 | **1.78** | **1.69** |  |
|  | Unigene19571_mk | 353 | 1.48e-05 | 1.78e-07 | **1.74** | **2.28** |  |
|  | Unigene38768_mk | 329 | 1.05e-04 | 8.77e-05 | **2.81** | **2.62** |  |
|  | Unigene51050_mk | 436 | 2.4e-03 | 1.07e-07 | **1.06** | **1.54** |  |
|  | Unigene9256_mk | 249 | 0.36174 | 3.54e-04 | **1.06** | **2.45** |  |
| **PxABCC2** | Unigene9793_mk | 3097 | 1.03e-05 | 1.12e-09 | **0.40** | **-0.57** | **No difference** |
|  | Unigene4442_mk | 874 | 0.059696 | 0.05061 | **-0.40** | **-0.41** |  |
|  | Unigene38738_mk | 795 | 0.31338 | 2.00e-04 | **0.29** | **0.71** |  |
|  | Unigene37960_mk | 435 | 0.72622 | 0.64617 | **-0.19** | **0.22** |  |
|  | Unigene38673_mk | 297 | 0.49641 | 0.52315 | **0.37** | **0.38** |  |
|  | Unigene38550_mk | 168 | 0.82201 | 0.21650 | **-0.26** | **-1.28** |  |
|  | Unigene38740_mk | 157 | 0.31559 | 0.16534 | **-0.78** | **-1.02** |  |
|  | Unigene5536_mk | 295 | 0.86565 | 0.35892 | **0.23** | **0.95** |  |
| **PxABCC3** | Unigene21845_mk | 829 | 8.79e-10 | 1.62e-20 | **-1.39** | **-2.39** | **Down regulation** |
|  | Unigene10673_mk | 822 | 5.44e-14 | 6.62e-14 | **-1.67** | **-1.64** |  |
|  | Unigene16611_mk | 580 | 1.66e-08 | 5.51e-21 | **-1.50** | **-3.11** |  |
|  | Unigene38737_mk | 663 | 2.67e-11 | 3.03e-12 | **-1.39** | **-1.45** |  |
|  | Unigene38739_mk | 615 | 1.18e-09 | 6.25e-15 | **-1.78** | **-2.51** |  |
|  | Unigene19663_mk | 600 | 5.14e-37 | 1.21e-65 | **-1.97** | **-3.22** |  |
|  | Unigene13602_mk | 342 | 5.87e-04 | 2.90e-11 | **-1.84** | **-13.76** |  |
|  | Unigene28969_mk | 179 | 1.44e-05 | 2.75e-04 | **-4.58** | **-3.02** |  |
| **PxABCC4** | Unigene36134_mk | 1221 | 9.50e-04 | 1.95e-07 | **0.22** | **0.31** | **No difference** |
|  | Unigene34850_mk | 759 | 1.11e-04 | 7.36e-04 | **0.38** | **0.34** |  |
|  | Unigene3098_mk | 772 | 3.00e-03 | 0.52189 | **0.27** | **0.09** |  |
|  | Unigene35508_mk | 429 | 2.42e-05 | 5.15e-07 | **0.29** | **0.33** |  |
|  | Unigene34841_mk | 330 | 0.012568 | 0.33724 | **0.48** | **0.22** |  |
| **PxABCC5** | Unigene17190_mk | 595 | 0.82868 | 0.31398 | **0.07** | **-0.29** | **No difference** |
|  | Unigene39089_mk | 346 | 0.76116 | 0.87451 | **-0.18** | **-0.10** |  |
|  | Unigene8985_mk | 393 | 0.39782 | 0.42049 | **-0.39** | **-0.38** |  |
|  | Unigene16286_mk | 307 | 0.073775 | 0.17931 | **-1.08** | **-0.84** |  |
|  | Unigene19514_mk | 277 | 0.76048 | 0.64621 | **-0.21** | **-0.34** |  |
|  | Unigene12417_mk | 259 | 0.98918 | 0.67402 | **-0.04** | **0.31** |  |
|  | Unigene34953_mk | 522 | 0.74043 | 0.92957 | **0.13** | **0.04** |  |
|  | Unigene50976_mk | 344 | 0.40129 | 0.087358 | **-0.64** | **-1.20** |  |
|  | Unigene55691_mk | 157 | 0.87467 | 0.90645 | **0.13** | **0.11** |  |
| **PxMAP4K4** | Unigene388_mk | 1696 | 4.38e-11 | 2.86e-11 | **0.61** | **0.58** | **Up regulation** |
|  | Unigene56928_mk | 660 | 6.65e-02 | 7.23e-03 | **0.50** | **0.65** |  |
|  | Unigene41378_mk | 527 | 0.79005 | 0.75066 | **0.30** | **0.39** |  |
|  | Unigene57773_mk | 433 | 1.08e-07 | 2.43e-04 | **1.09** | **0.83** |  |
|  | Unigene37700_mk | 498 | 0.57469 | 0.75694 | **1.06** | **0.78** |  |
|  | Unigene37701_mk | 513 | 1.00236 | 0.85121 | **0.06** | **0.36** |  |
|  | Unigene32311_mk | 174 | 0.73702 | 0.65497 | **-9.38** | **1.62** |  |

The unigenes were identified by searching against a custom *P. xylostella* midgut transcriptome with the full-length cDNA sequence of the corresponding cDNA sequences as queries. The expression level of these unigenes was derived from RNA-Seq libraries before filtration under the arbitrary threshold (FDR value < 0.001) and the absolute expression value (log_2_ratio≧1) in a recent RNA-Seq study .

^a^FDR value, the False Discovery Rate value when performing Gene Ontology (GO) functional enrichment analysis using Blast2GO software with Fisher’s exact test.

^b^FC, Fold Change in gene expression [log_2_ Ratio (MK or GK-RPKM/MM-RPKM)]. MM: DBM1Ac-S; MK: DBM1Ac-R; GK: GZ-R.

1. Xie W, Lei Y, Fu W, Yang Z, Zhu X, Guo Z, et al. Tissue-specific transcriptome profiling of *Plutella xylostella* third instar larval midgut. Int J Biol Sci. 2012;8: 1142–1155.

2. Lei Y, Zhu X, Xie W, Wu Q, Wang S, Guo Z, et al. Midgut transcriptome response to a Cry toxin in the diamondback moth, *Plutella xylostella* (Lepidoptera: Plutellidae). Gene. 2014;533: 180–187.
