# Supplementary material for: MAPK Signaling Pathway Alters Expression of Midgut ALP and ABCC Genes and Causes Resistance to Bacillus thuringiensis Cry1Ac Toxin in Diamondback Moth
Source: PLoS Genet. 2015 Apr 13;11(4):e1005124. doi: 10.1371/journal.pgen.1005124 (PMC4395465; doi:10.1371/journal.pgen.1005124)
Supplement: S5 Table — (DOC) [file pgen.1005124.s017.doc]

**S5 Table. List of primers used for PxABCC1 study.**

| Purpose | Primer name | Primer sequence (5′-3′) | PCR product size (bp) | Positions (bp)^a^ |
| --- | --- | --- | --- | --- |
| **1.Length polymorphism analysis** |  |  |  |  |
| cDNA overlapping fragment 1 | C1-F1 | TGGTGAGTGGTAATAAGG | 1061 | 2–1062  Exon1–Exon6 |
|  | C1-R1 | AAGCAGCGAGTAGGAGAG |  |  |
| cDNA overlapping fragment 2 | C1-F2 | TGGACGAAATCATTTGCG | 916 | 863–1778  Exon5–Exon11 |
|  | C1-R2 | GTCTGGTCTCCGTGGGGG |  |  |
| cDNA overlapping fragment 3 | C1-F3 | AAGAAGTACAAGGAGGTG | 1018 | 1705–2722  Exon10–Exon16 |
|  | C1-R3 | AAGAGTTGGTGTCAAAGA |  |  |
| cDNA overlapping fragment 4 | C1-F4 | CTCTACGTGTATTCTGCC | 1016 | 2563–3578  Exon16–Exon22 |
|  | C1-R4 | TCAATGAGTATTTCTCCC |  |  |
| cDNA overlapping fragment 5 | C1-F5 | CTTCCCGTACTCAAGAAC | 914 | 3439–4353  Exon21–Exon25 |
|  | C1-R5 | CAGAAAGTCACCCAAAAT |  |  |
| **2. Whole PxABCC1 CDS amplification** | fC1-F | TCAAAAACACATTCAAAA | 4383 | -17–4367 |
|  | fC1-R | AAGAAAGTCCGTAACAGA |  |  |
| **3.qPCR analysis** | qC1-F | GGTGGTGCTCATCTGCTACCTCAT | 165 | 696–860 |
|  | qC1-R | ATCCTGACACGCTCATCGGTTTT |  |  |
|  | qL32-F | CCAATTTACCGCCCTACC | 120 | — |
|  | qL32-R | TACCCTGTTGTCAATACCTCT |  |  |

^a^Positions corresponding to the full-length cDNA sequence of *P. xylostella* *ABCC1* gene (GenBank accession no. KM245560).
