# Supplementary material for: MAPK Signaling Pathway Alters Expression of Midgut ALP and ABCC Genes and Causes Resistance to Bacillus thuringiensis Cry1Ac Toxin in Diamondback Moth
Source: PLoS Genet. 2015 Apr 13;11(4):e1005124. doi: 10.1371/journal.pgen.1005124 (PMC4395465; doi:10.1371/journal.pgen.1005124)
Supplement: S6 Table — (DOC) [file pgen.1005124.s018.doc]

**S6 Table. List of primers used for PxABCC2 study.**

| Purpose | Primer name | Primer sequence (5′-3′) | PCR product size (bp) | Positions (bp)^c^ |
| --- | --- | --- | --- | --- |
| **1. Transmembrane 7-12 amplification** | TM-F | TATTCCCAATGCAGCAAAG | 1234 | 2088–3321 |
|  | TM-R | GTCCTCCAACGAGTAAGTCAG |  |  |
| **2. Length polymorphism analysis^a^** |  |  |  |  |
| cDNA overlapping fragment 1 | C2-F1 | GGAAAGAGTCGGAAGAGAA | 978 | 22–999  Exon1–Exon6 |
|  | C2-R1 | CTCCGTGAACAGCATGAACC |  |  |
| cDNA overlapping fragment 2 | C2-F2 | CCTTCATGTTCCTGCACTA | 915 | 980–1548  Exon4–Exon10 |
|  | C2-R2 | GACACCGGAAGCTCTTTCA |  |  |
| cDNA overlapping fragment 3 | C2-F3 | TCCAAGAAGGAGGACGACAC | 953 | 1306–2258  Exon8–Exon14 |
|  | C2-R3 | GAGTCCACGGAGACCAGGTA |  |  |
| cDNA overlapping fragment 4 | C2-F4 | TATTCGATGACCCCTTATCG | 1157 | 1823–2979  Exon12–Exon19 |
|  | C2-R4 | CCCGATGTAAGTGTGGAAGG |  |  |
| cDNA overlapping fragment 5 | C2-F5 | TCCAGTGTTTGGAATGATCG | 1144 | 2844–3987  Exon18–Exon26 |
|  | C2-R5 | CATGTTGTCTCCGGTCTCCT |  |  |
| **3. Whole PxABCC2 CDS amplification** | fC2-F | CCATTGTATGGGTATTAGGTG | 4119 | -60–4059 |
|  | fC2-R | CTAAGGTACAAGCTATCATTGAG |  |  |
| **4.qPCR analysis** | qC2-F | AGTCTTGGCACGCAAACGG | 103 | 2401–2503 |
|  | qC2-R | CGAACAGACGCATGAAGGACAT |  |  |
|  | qL32-F | CCAATTTACCGCCCTACC | 120 | — |
|  | qL32-R | TACCCTGTTGTCAATACCTCT |  |  |
| **5.dsRNA synthesis^b^** | dsC2-F | T7-GGCGTCGTCCACTGACTACTG | 603 | 2310–2866 |
|  | dsC2-R | T7-AGCCGATCATTCCAAACACT |  |  |
|  | dsEGFP-F | T7-CCACAAGTTCAGCGTGTCCG | 469 | — |
|  | dsEGFP-R | T7-AAGTTCACCTTGATGCCGTTC |  |  |

^a^Nested PCR of overlapping cDNA fragments using primer combinations reported by Baxter et al. .

^b^Forward and reverse primers to synthetize dsRNA template have T7 RNA polymerase promoter sequence (5′-TAATACGACTCACTATAGGGAGA-3′) appended to their 5′ and 3′ ends, respectively.

^c^Positions corresponding to the corrected full-length cDNA sequence (GenBank: KM245561) or full-length gDNA (GenBank: JN030495) sequence of the *P. xylostella* *ABCC2* gene.

1. Baxter SW, Badenes-Pérez FR, Morrison A, Vogel H, Crickmore N, Kain W, et al. Parallel evolution of *Bacillus thuringiensis* toxin resistance in Lepidoptera. Genetics. 2011;189: 675–679.
