# Supplementary material for: MAPK Signaling Pathway Alters Expression of Midgut ALP and ABCC Genes and Causes Resistance to Bacillus thuringiensis Cry1Ac Toxin in Diamondback Moth
Source: PLoS Genet. 2015 Apr 13;11(4):e1005124. doi: 10.1371/journal.pgen.1005124 (PMC4395465; doi:10.1371/journal.pgen.1005124)
Supplement: S7 Table — (DOC) [file pgen.1005124.s019.doc]

**S7 Table. List of primers used for PxABCC3 study.**

| Purpose | Primer name | Primer sequence (5′-3′) | PCR product size (bp) | Positions (bp)^b^ |
| --- | --- | --- | --- | --- |
| **1.Length polymorphism analysis** |  |  |  |  |
| cDNA overlapping fragment 1 | C3-F1 | GTTGCGGAGGATGTGTTG | 1003 | 13–1015  Exon1–Exon6 |
|  | C3-R1 | CGTAGGTGACGATGGTGA |  |  |
| cDNA overlapping fragment 2 | C3-F2 | ACGCCCGGATCAAAATTA | 885 | 806–1690  Exon5–Exon11 |
|  | C3-R2 | TCTCCAGCGCACACACTC |  |  |
| cDNA overlapping fragment 3 | C3-F3 | GGGTCTGTCGAGTGTGTG | 970 | 1664–2633  Exon11–Exon17 |
|  | C3-R3 | GAGCCGATGTCCTTGGTG |  |  |
| cDNA overlapping fragment 4 | C3-F4 | GGCTTTGCTCTGTTCCTC | 1110 | 2254–3363  Exon14–Exon21 |
|  | C3-R4 | CACCTGCCATCCGCTTTC |  |  |
| cDNA overlapping fragment 5 | C3-F5 | ATACCGACTGAGGACAAC | 906 | 3202–4107  Exon20–Exon25 |
|  | C3-R5 | CCCCTTACACACATAACA |  |  |
| **2. Whole PxABCC3 CDS amplification** | fC3-F | AGGTTGCGGAGGATGTGT | 4167 | 11–4177 |
|  | fC3-R | TTTGTGGGAGGAGTGGGT |  |  |
| **3.qPCR analysis** | qC3-F | TCAACCGCTTCACCAAGGACAT | 111 | 2606–2716 |
|  | qC3-R | CGGCGTTCAGCACCAGGAT |  |  |
|  | qL32-F | CCAATTTACCGCCCTACC | 120 | — |
|  | qL32-R | TACCCTGTTGTCAATACCTCT |  |  |
| **4.dsRNA synthesis^a^** | dsC3-F | T7-GCATCCTCAACCGCTTCAC | 531 | 2600–3084 |
|  | dsC3-R | T7-CACGCTGCCCACTGCTACT |  |  |
|  | dsEGFP-F | T7-CCACAAGTTCAGCGTGTCCG | 469 | — |
|  | dsEGFP-R | T7-AAGTTCACCTTGATGCCGTTC |  |  |

^a^Forward and reverse primers to synthesize dsRNA template have T7 RNA polymerase promoter sequence (5′-TAATACGACTCACTATAGGGAGA-3′) appended to their 5′ and 3′ ends, respectively.

^b^Positions corresponding to the full-length cDNA sequence of *P. xylostella ABCC3* gene deposited in GenBank database (accession no. KM245562).
