# Supplementary material for: MAPK Signaling Pathway Alters Expression of Midgut ALP and ABCC Genes and Causes Resistance to Bacillus thuringiensis Cry1Ac Toxin in Diamondback Moth
Source: PLoS Genet. 2015 Apr 13;11(4):e1005124. doi: 10.1371/journal.pgen.1005124 (PMC4395465; doi:10.1371/journal.pgen.1005124)
Supplement: S8 Table — (DOC) [file pgen.1005124.s020.doc]

**Table S8. List of primers used for PxABCC4 study.**

| Purpose | Primer name | Primer sequence (5′-3′) | PCR product size (bp) | Positions (bp)^a^ |
| --- | --- | --- | --- | --- |
| **1.Length polymorphism analysis** |  |  |  |  |
| cDNA overlapping fragment 1 | C4-F1 | AAACCCACATGATAAGGC | 948 | 72–1019  Exon2–Exon7 |
|  | C4-R1 | AATACCGACAGGGCAGTC |  |  |
| cDNA overlapping fragment 2 | C4-F2 | TTTTATATTCCGCACTTC | 1162 | 966–2127  Exon7–Exon13 |
|  | C4-R2 | ATCATCATCTTCGTCGTC |  |  |
| cDNA overlapping fragment 3 | C4-F3 | GAGACGCAGGGCAAGTTC | 956 | 1942–2897  Exon12–Exon17 |
|  | C4-R3 | GCCGTGTTCAGGTAAAGC |  |  |
| cDNA overlapping fragment 4 | C4-F4 | GCTCAATCAACCTCCACA | 1359 | 2618–3976  Exon16–Exon25 |
|  | C4-R4 | GACCAGCATCCATCACAA |  |  |
| **2. Whole PxABCC4 CDS amplification** | fC4-F | CTTCTTTTATTATCTTTG | 4282 | -122–4160 |
|  | fC4-R | CTATTTTATTAGTTTTCT |  |  |
| **3.qPCR analysis** | qC4-F | TGAATTAACAACCGTTCTACCA | 141 | 2376–2516 |
|  | qC4-R | ATTTCTCCGTCGTCTCCC |  |  |
|  | qL32-F | CCAATTTACCGCCCTACC | 120 | — |
|  | qL32-R | TACCCTGTTGTCAATACCTCT |  |  |

^a^Positions corresponding to the full-length cDNA sequence of *P. xylostella ABCC4* gene deposited in GenBank database (accession no. KM245563).
