# Supplementary material for: MAPK Signaling Pathway Alters Expression of Midgut ALP and ABCC Genes and Causes Resistance to Bacillus thuringiensis Cry1Ac Toxin in Diamondback Moth
Source: PLoS Genet. 2015 Apr 13;11(4):e1005124. doi: 10.1371/journal.pgen.1005124 (PMC4395465; doi:10.1371/journal.pgen.1005124)
Supplement: S9 Table — (DOC) [file pgen.1005124.s021.doc]

**S9 Table. List of primers used for PxABCC5 study.**

| Purpose | Primer name | Primer sequence (5′-3′) | PCR product size (bp) | Positions (bp)^a^ |
| --- | --- | --- | --- | --- |
| **1.Length polymorphism analysis** |  |  |  |  |
| cDNA overlapping fragment 1 | C5-F1 | GACTCAAGTATGGATTCA | 1227 | -9–1218  Exon1–Exon7 |
|  | C5-R1 | TTTTCCGTTTGCTGTTAT |  |  |
| cDNA overlapping fragment 2 | C5-F2 | TAACAGCAAACGGAAAAG | 1057 | 1202–2258  Exon7–Exon13 |
|  | C5-R2 | ATCCAGTAAGTGAGCCAC |  |  |
| cDNA overlapping fragment 3 | C5-F3 | TCATCCTCGTCACGCACC | 1062 | 1832–2893  Exon10–Exon17 |
|  | C5-R3 | TCACACCCTCCACCCTCT |  |  |
| cDNA overlapping fragment 4 | C5-F4 | TGCAGGGGATCAAGAGGG | 1228 | 2864–4091  Exon16–Exon24 |
|  | C5-R4 | TCGGCGTAGCGGATGTGT |  |  |
| **2. Whole PxABCC5 CDS amplification** | fC5-F | ACATTGTCTCTCGTCTTT | 4265 | -158–4107 |
|  | fC5-R | GTACTAATTTTGTTCGTC |  |  |
| **3.qPCR analysis** | qC5-F | AGGGACAGGTGCTCATTGACG | 115 | 3488–3602 |
|  | qC5-R | TAGCGGAGGCTGGCGGAAA |  |  |
|  | qL32-F | CCAATTTACCGCCCTACC | 120 | — |
|  | qL32-R | TACCCTGTTGTCAATACCTCT |  |  |

^a^Positions corresponding to the full-length cDNA sequence of *P. xylostella ABCC5* gene deposited in GenBank database (accession no. KM245564).
