# Supplementary material for: MAPK Signaling Pathway Alters Expression of Midgut ALP and ABCC Genes and Causes Resistance to Bacillus thuringiensis Cry1Ac Toxin in Diamondback Moth
Source: PLoS Genet. 2015 Apr 13;11(4):e1005124. doi: 10.1371/journal.pgen.1005124 (PMC4395465; doi:10.1371/journal.pgen.1005124)
Supplement: S10 Table — (DOC) [file pgen.1005124.s022.doc]

**S10 Table. Genomic structure of three *P. xylostella* ABCC genes in the *BtR-1* locus.**

| **PxABCC1(Px002418+19)** | | | | **PxABCC2(Px002416, JN030495)** | | | | **PxABCC3(Px002414+15)** | | | |
| --- | --- | --- | --- | --- | --- | --- | --- | --- | --- | --- | --- |
| Exon number | Exon size | Intron phase | Intron size | Exon number | Exon size | Intron phase | Intron size | Exon number | Exon size | Intron phase | Intron size |
| 1 | **224** | **2** | 15108 | 1 | **206** | **2** | 2163 | 1 | **188** | **2** | 1161 |
| 2 | **135** | **2** | 500 | 2 | **132** | **2** | 736 | 2 | **132** | **2** | 429 |
| 3 | **205** | **0** | 255 | 3 | **205** | **0** | 3564 | 3 | **205** | **0** | 332 |
| 4 | **226** | **1** | 213 | 4 | **226** | **1** | 587 | 4 | **226** | **1** | 957 |
| 5 | **98** | **0** | 335 | 5 | **98** | **0** | 779 | 5 | **98** | **0** | 1416 |
| 6 | **198** | **0** | 196 | 6 | **198** | **0** | 393 | 6 | **198** | **0** | 2334 |
| 7 | **127**^a^ | **1** | 1186 | 7 | **127** | **1** | 455 | 7 | **127** | **1** | 344 |
| 8 | **147** | **1** | 186 | 8 | **147** | **1** | 818 | 8 | **144** | **1** | 513 |
| 9 | **194** | **0** | 84 | 9 | **167** | **0** | 935 | 9 | **182** | **0** | 275 |
| 10 | **165** | **0** | 81 | 10 | **165** | **0** | 332 | 10 | **165** | **0** | 284 |
| 11 | **138** | **0** | 365 | 11 | **138** | **0** | 305 | 11 | **138** | **0** | 911 |
| 12 | **168** | **0** | 825 | 12 | **168** | **0** | 75 | 12 | **168** | **0** | 759 |
| 13 | **141** | **0** | 276 | 13 | **120** | **0** | 77 | 13 | **117** | **0** | 3723^‡^ |
| 14 | **218** | **2** | 275 | 14 | **245** | **2** | 85 | 14 | **248** | **2** | 578 |
| 15 | **131** | **1** | 893 | 15 | **53** | **1** | 268 | 15 | **50** | **1** | 466 |
| 16 | **210** | **1** | 318^b^ | 16 | **210** | **1** | 330 | 16 | **210** | **1** | 2424 |
| 17 | **231** | **1** | 268 | 17 | **231** | **1** | 277 | 17 | **231** | **1** | 235 |
| 18 | **107** | **0** | 958 | 18 | **107** | **0** | 170 | 18 | **107** | **0** | 219 |
| 19 | **124** | **1** | 612 | 19 | **124** | **1** | 2468 | 19 | **124** | **1** | 353 |
| 20 | **171** | **1** | 348 | 20 | **174** | **1** | 331 | 20 | **174** | **1** | 261 |
| 21 | **128** | **0** | 361 | 21 | **128** | **0** | 278 | 21 | **128** | **0** | 357 |
| 22 | **123** | **0** | 254 | 22 | **123** | **0** | 687 | 22 | **123** | **0** | 358 |
| 23 | **123** | **0** | 244 | 23 | **123** | **0** | 295 | 23 | **123** | **0** | 589 |
| 24 | **161** | **2** | 295 | 24 | **161** | **2** | 775 | 24 | **161** | **2** | 388 |
| 25 | **274** |  |  | 25 | **118** | **0** | 287 | 25 | **280** |  |  |
|  |  |  |  | 26 | **150** |  |  |  |  |  |  |

The genomic sequences of *PxABCC1* and *PxABCC3* is derived from the Diamondback moth Genome Database (DBM-DB: http://iae.fafu.edu.cn/DBM/search.php) and combined under the Gene ID number Px002418+Px002419 and Px002414+Px002415, the genomic sequences of ABCC2 is according to the sequences in GenBank database (accession no. JN030495) and DBM-DB database under the Gene ID number Px002416.

^a^There are two alternative splicing exons of Exon7 of *PxABCC1*, the Exon7a is listed here, Exon7b (position within the combined genomic sequence of Px002418+Px002419: 18456-18582bp) has the same size (127bp) as Exon7a.

^b^The sequences of these introns are not completed in the corresponding genomic sequences.
