# Supplementary material for: MAPK Signaling Pathway Alters Expression of Midgut ALP and ABCC Genes and Causes Resistance to Bacillus thuringiensis Cry1Ac Toxin in Diamondback Moth
Source: PLoS Genet. 2015 Apr 13;11(4):e1005124. doi: 10.1371/journal.pgen.1005124 (PMC4395465; doi:10.1371/journal.pgen.1005124)
Supplement: S11 Table — (DOC) [file pgen.1005124.s023.doc]

**S11 Table. Effect of silencing PxABCC2, PxABCC3 and multigenes on biological parameters of *P. xylostella* strain DBM1Ac-S.**

| Treatment | Pupation (%) | Pupal weight (mg) | Pupation duration (days) | Eclosion (%) |
| --- | --- | --- | --- | --- |
| Buffer-R^a^ | 96.67±3.33**a** | 5.34±0.12**a** | 4.53±0.15**a** | 89.63±0.37**a** |
| Buffer-S | 96.67±3.33**a** | 5.58±0.10**a** | 4.61±0.16**a** | 96.30±3.70**a** |
| dsEGFP | 93.33±3.33**a** | 5.47±0.26**a** | 4.57±0.12**a** | 92.96±3.53**a** |
| dsPxABCC2 | 73.33±8.82**b** | 4.06±0.29**b** | 4.01±0.23**b** | 68.25±1.59**b** |
| dsPxABCC3 | 73.33±3.33**b** | 4.18±0.59**b** | 4.11±0.20**ab** | 76.79±5.36**b** |
| dsMultigenes | 70.00±5.77**b** | 3.88±0.51**b** | 3.88±0.13**b** | 66.87±2.58**b** |

Mean values ± SEM followed by different letters in each column denote significant difference by LSD test (P < 0.05; n = 3).

^a^This represents buffer-injected larvae from NIL-R strain as a negative control.
