# Supplementary material for: MAPK Signaling Pathway Alters Expression of Midgut ALP and ABCC Genes and Causes Resistance to Bacillus thuringiensis Cry1Ac Toxin in Diamondback Moth
Source: PLoS Genet. 2015 Apr 13;11(4):e1005124. doi: 10.1371/journal.pgen.1005124 (PMC4395465; doi:10.1371/journal.pgen.1005124)
Supplement: S12 Table — (DOC) [file pgen.1005124.s024.doc]

**S12 Table. List of primers used for PxMAP4K4 study.**

| Purpose | Primer name | Primer sequence (5′-3′) | PCR product size (bp) | Positions (bp)^b^ |
| --- | --- | --- | --- | --- |
| **1.Length polymorphism analysis** |  |  |  |  |
| cDNA overlapping fragment 1 | MA-F1 | GCATCAACTGGCTCCGTCTG | 1148 | 6–1153  Exon1–Exon8 |
|  | MA-R1 | CTCGCTTGCTGTTCCTCTTT |  |  |
| cDNA overlapping fragment 2 | MA-F2 | AAAGTCAAAGAAATGGGCAAAG | 2018 | 768–2785  Exon6–Exon15 |
|  | MA-R2 | ACCCGAACGTCAGGAACG |  |  |
| cDNA overlapping fragment 3 | MA-F3 | CGTTCCTGACGTTCGGGTTC | 1104 | 2768–3871  Exon15–Exon20 |
|  | MA-R3 | CCATGCCGGGCTTGTTGA |  |  |
| **2. Whole** Px**MAP4K4 CDS amplification** | fMA-F | GCATCAACTGGCTCCGTCTG | 4018 | 6–4023 |
|  | fMA-R | ATCGCTAACAATCTCAGGTCACTA |  |  |
| **3.qPCR analysis** | qMA-F | CATCAACTGGCTCCGTCTG | 188 | 7–215 |
|  | qMA-R | TCATCTTCGGTGACATCCATC |  |  |
|  | qL32-F | CCAATTTACCGCCCTACC | 120 | — |
|  | qL32-R | TACCCTGTTGTCAATACCTCT |  |  |
| **4.dsRNA synthesis^a^** | dsMA-F | T7-GCCCGAGATACGCAAATACA | 582 | 2739–3274 |
|  | dsMA-R | T7-CCGAGCCATAGATCACTTTCA |  |  |
|  | dsEGFP-F | T7-CCACAAGTTCAGCGTGTCCG | 469 | — |
|  | dsEGFP-R | T7-AAGTTCACCTTGATGCCGTTC |  |  |

^a^Forward and reverse primers to synthetize dsRNA template have T7 RNA polymerase promoter sequence (5′-TAATACGACTCACTATAGGGAGA-3′) appended to their 5′ and 3′ ends, respectively.

^b^Positions corresponding to the corrected full-length CDS sequence of *P. xylostella MAP4K4* gene (GenBank accession no. KM507871).
